# Structural analysis of mitochondrial rRNA gene variants identified in patients with deafness

**DOI:** 10.3389/fphys.2023.1163496

**Published:** 2023-06-08

**Authors:** Antón Vila-Sanjurjo, Natalia Mallo, Joanna L. Elson, Paul M. Smith, Emma L. Blakely, Robert W. Taylor

**Affiliations:** ^1^ Grupo GIBE. Departamento de Bioloxía e Centro Interdisciplinar de Química e Bioloxía (CICA), Universidade da Coruña (UDC), A Coruña, Spain; ^2^ The Bioscience Institute, Newcastle University, Newcastle upon Tyne, United Kingdom; ^3^ Human Metabolomics, North-West University, Potchefstroom, South Africa; ^4^ Department of Paediatrics, Raigmore Hospital, Inverness, United Kingdom; ^5^ Wellcome Centre for Mitochondrial Research, Translational and Clinical Research Institute, Faculty of Medical Sciences, Newcastle University, Newcastle upon Tyne, United Kingdom; ^6^ NHS Highly Specialised Service for Rare Mitochondrial Disorders, Newcastle upon Tyne Hospitals NHS Foundation Trust, Newcastle upon Tyne, United Kingdom

**Keywords:** mito-ribosome, mtDNA, mitochondrial rRNA mutations, mito-ribosomal fidelity, mtDNA diseases, deafness (hearing loss), haplotype

## Abstract

The last few years have witnessed dramatic advances in our understanding of the structure and function of the mammalian mito-ribosome. At the same time, the first attempts to elucidate the effects of mito-ribosomal fidelity (decoding accuracy) in disease have been made. Hence, the time is right to push an important frontier in our understanding of mitochondrial genetics, that is, the elucidation of the phenotypic effects of mtDNA variants affecting the functioning of the mito-ribosome. Here, we have assessed the structural and functional role of 93 mitochondrial (mt-) rRNA variants thought to be associated with deafness, including those located at non-conserved positions. Our analysis has used the structural description of the human mito-ribosome of the highest quality currently available, together with a new understanding of the phenotypic manifestation of mito-ribosomal-associated variants. Basically, any base change capable of inducing a fidelity phenotype may be considered non-silent. Under this light, out of 92 previously reported mt-rRNA variants thought to be associated with deafness, we found that 49 were potentially non-silent. We also dismissed a large number of reportedly pathogenic mtDNA variants, 41, as polymorphisms. These results drastically update our view on the implication of the primary sequence of mt-rRNA in the etiology of deafness and mitochondrial disease in general. Our data sheds much-needed light on the question of how mt-rRNA variants located at non-conserved positions may lead to mitochondrial disease and, most notably, provide evidence of the effect of haplotype context in the manifestation of some mt-rRNA variants.

## Introduction

**FIGURE 1:**
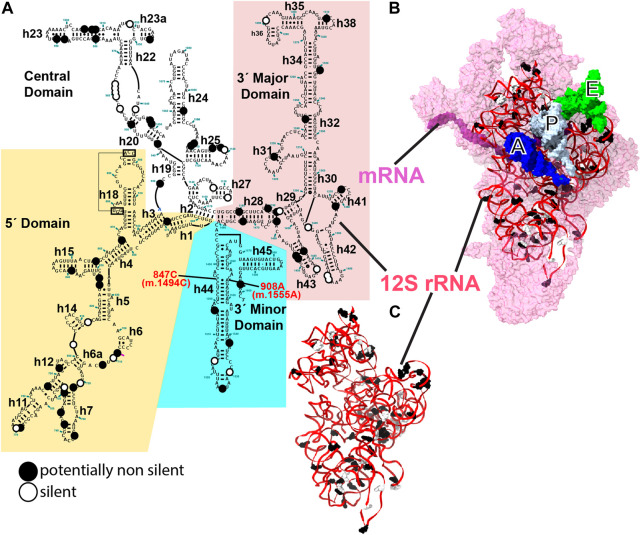
12S mt-rRNA and mito-ribosomal SSU. **(A)** Secondary-structure map of the human 12S mt-rRNA with potentially pathogenic variants indicated with large dots. Black dots, putatively non-silent variants, white dots, variants deemed as silent. 12S mt-rRNA domains are indicated with background colors. Helix numbers are indicated. The position of the two known ototoxic variants is indicated with red, bold font. **(B)** Inter-subunit face of the SSU as observed in the 2.2-Å cryo-EM structure of the human mito-ribosome (Itoh et al., 2021; Itoh et al., 2022). 12S mt-rRNA, red ribbon; SSU r-proteins, pink surface. Surface representations of the A-, P-, and E-site tRNAs are shown in blue, white, and green, respectively. **(B)** Three-dimensional structure of 12S mt-rRNA as seen in **(B)**. Sites of variation are shown in **(B, C)**. The secondary structure map of the human mt-SSU was kindly provided by Dr. Alan Brown.

Aminoglycoside antibiotics (AGs) are some of the most commonly prescribed antibacterials, despite their well-known capacity to cause toxic side effects to the kidneys and inner ear (Huth et al., 2011). Although their use in the industrialized world is usually limited to severe infections, they remain very popular in the developing world due to their low cost and potent antibacterial activities (Huth et al., 2011). Maternally inherited susceptibility was identified as a major cause of AG ototoxicity, with ∼1/3 of patients carrying a single mitochondrial variant, the 908A>G (m.1555A>G) base change in mt-12S rRNA (Figure 1A) (Hu et al., 1991a; Prezant et al., 1993; Fischel-Ghodsian, 2005). Note that mt-rRNA variants will be cited in the text by both their gene and genomic position, with the latter in brackets and preceded by “m”. Genomic positions are defined according to the Cambridge reference sequence for human mitochondrial DNA (Andrews et al., 1999). In the case of 908A>G (m.1555A>G), the presence of the mutation was not sufficient to induce a clinical phenotype, requiring the presence of other factors, such as AGs or nuclear modifier genes (Guan et al., 1996; Guan et al., 2000; Fu et al., 2021). A second variant at position 847C>U (m.1494C>U) of mt-12S rRNA (Figure 1A) has also been unambiguously identified as a causative agent of deafness and an AG susceptibility marker (Zhao H. et al., 2004). As of now, 847C>U (m.1494C>U) and 908A>G (m.1555A>G) remain the only proven mt-rRNA ototoxic variants, although many reports have claimed the identification of additional mt-rRNA variants associated with hearing loss. Unfortunately, no accompanying structural and/or biochemical characterization is available for these suspect variants, which can be used to ascertain their pathogenic potential, leaving the issue of whether they can be considered causative agents of deafness unresolved.

Our understanding of the mito-ribosome has advanced much more slowly than that of other ribosomal systems. In particular, the issue of the maintenance of mito-ribosomal fidelity, defined as the accuracy of decoding during mitochondrial translation, could not be tackled until very recently. Despite this, evidence obtained mostly from studies with chimeric mito-bacterial ribosomes, as well as in yeast, has been successfully used to ascertain that decreased mito-ribosomal fidelity was behind the deleterious effect caused by the two known ototoxic variants (Weiss-Brummer and Huttenhofer., 1989; Hobbie et al., 2008a; Hobbie et al., 2008b; He et al., 2013). The recent introduction of mutations in the nuclear genes encoding mito-ribosomal proteins has finally allowed us to take a direct glance at the phenotypes associated with impaired mito-ribosomal fidelity (Akbergenov et al., 2018; Suhm et al., 2018; Ferreira et al., 2019) (reviewed Vila-Sanjurjo et al., 2023). As a result, the theoretical framework for the evaluation of the pathogenic potential of mt-rRNA variants has shifted. Basically, any base change capable of inducing a fidelity phenotype may be considered non-silent under this new framework (Vila-Sanjurjo et al., 2023). At the same time, the availability of near-atomic resolution structures of mammalian mito-ribosome structures caught in the process of translation finally permits an assessment of their disruptive potential on mt-RNA structure. In particular, the recent 2.2-Å cryo-EM structure of the human mito-ribosome, generated by the Amunts laboratory, has the highest resolution ever achieved for a mito-ribosome and resulted in an extremely well-refined model (Itoh et al., 2021; Itoh et al., 2022), which permits the structural dissection of mt-rRNA variants with unprecedented detail. The new theoretical framework, together with the latest structural tools, was used here to structurally dissect the large collection of mt-rRNA variants reportedly associated with deafness, aiming at ascertaining which base changes could lead to non-silent phenotypes. In addition to underscoring the crucial role of the primary sequence of mt-rRNA in the etiology of deafness, our work provides a new paradigm for the role of mt-rRNA variants in mitochondrial disease, highlighting the potential pathogenicity of variants located at non-conserved positions of mt-rRNA and even of haplotype markers.

## Results

In this analysis, we used a total of 83 and 9 variants, identified in deafness patients, that mapped to the mito-ribosomal small and large subunit (SSU and LSU) mt-rRNAs, respectively. The variants originated from 35 studies reported in the literature (80 variants), MITOMAP (2 variants), and this work (1 variant) (Supplementary Table S1) (Ruiz-Pesini et al., 2007). Figure 1 shows the positions of the variants displayed on the secondary-structure map of the human 12S mt-rRNA (Figure 1A), the three-dimensional structure of the SSU (Figure 1B), and the three-dimensional structure of 12S mt-rRNA (Figure 1C). In contrast to previous studies in which we focused on extremely rare variants (Elson et al., 2015a; Vila-Sanjurjo et al., 2021), here, we included variants with medium-to-no structural conservation and with moderate to high abundance in the population (Figure 1). Even haplotype markers circumstantially associated with deafness (33 in total) were investigated in this study (Supplementary Table S1). Mt-rRNA variants are shown in bold font while all other positions mentioned in the text are shown in regular font.

The low conservation of most variants made the comparison to heterologous structures largely useless, implying that the type of evidence used in our previous heterologous inferential analysis (HIA) studies with exceptionally rare mt-rRNA variants could not be raised in this case (Smith et al., 2014; Elson et al., 2015a; Elson et al., 2015b; Vila-Sanjurjo et al., 2021). In the absence of such evidence, our goal was merely to establish, from the structural point of view, which base changes could lead to non-silent variants, particularly considering their potential to alter mito-ribosomal fidelity. In all cases, we checked the validity of our analysis by confirming the adjustment of the mito-ribosomal model to the cryo-EM density map.

More than half of the 92 variants (i.e., 49) could cause structural distortions, possibly resulting in some degree of defective mito-ribosomal function and constituting valid candidates for deafness-inducing variants under the new theoretical framework for the evaluation of the pathogenic potential of mt-rRNA variants. Forty-one variants, including the nine mapping to 16S mt-rRNA, were deemed likely silent and two unclear (Supplementary Table S1, see also Supplementary Information). Putatively non-silent and silent SSU variants are shown in Figure 1C. A total of 27, 16, and 26 of the potentially non-silent variants were likely involved in the maintenance of the secondary, tertiary, and quaternary structures of mito-ribosomal SSU, respectively (Supplementary Table S1). Tertiary interactions were defined as those RNA-RNA contacts not present in the secondary structure maps of mt-rRNA (Figure 1A). Additionally, 8 variants potentially affected the interaction with mito-ribosomal ligands, 8 possibly interfered with the function of inter-subunit bridges, and 28 variants lay in close proximity to early binding mito-ribosomal proteins or mito-ribosomal proteins known to harbor pathogenic mutations (Supplementary Table S1). Note that all these categories may overlap (Supplementary Table S1). Strikingly, out of 33 haplotype markers included in the analysis, according to MITOMAP (Ruiz-Pesini et al., 2007), 17 were considered potentially non-silent.

This section contains the structural description of all 49 variants regarded as potentially non-silent. Given the number of variants analyzed, most accompanying figures are provided as Supplementary Material.

### Variants in the proximity of mito-ribosomal proteins harboring pathogenic mutations and/or early binding proteins

The existence of pathogenic mutations in several mt-SSU mito-ribosomal proteins is a good indication that variants in mt-rRNA residues interacting with these proteins may also lead to deleterious phenotypes. Early binding proteins are also of particular interest, as they constitute key nucleation sites for the assembly of the mt-SSU. Hence, mt-rRNA variants that interfere with the binding of these proteins could lead to defective mito-ribosomal assembly and other functional defects. All these variants will be discussed in this section.

#### Variants in the proximity of MRPS12/uS12m

**FIGURE 2:**
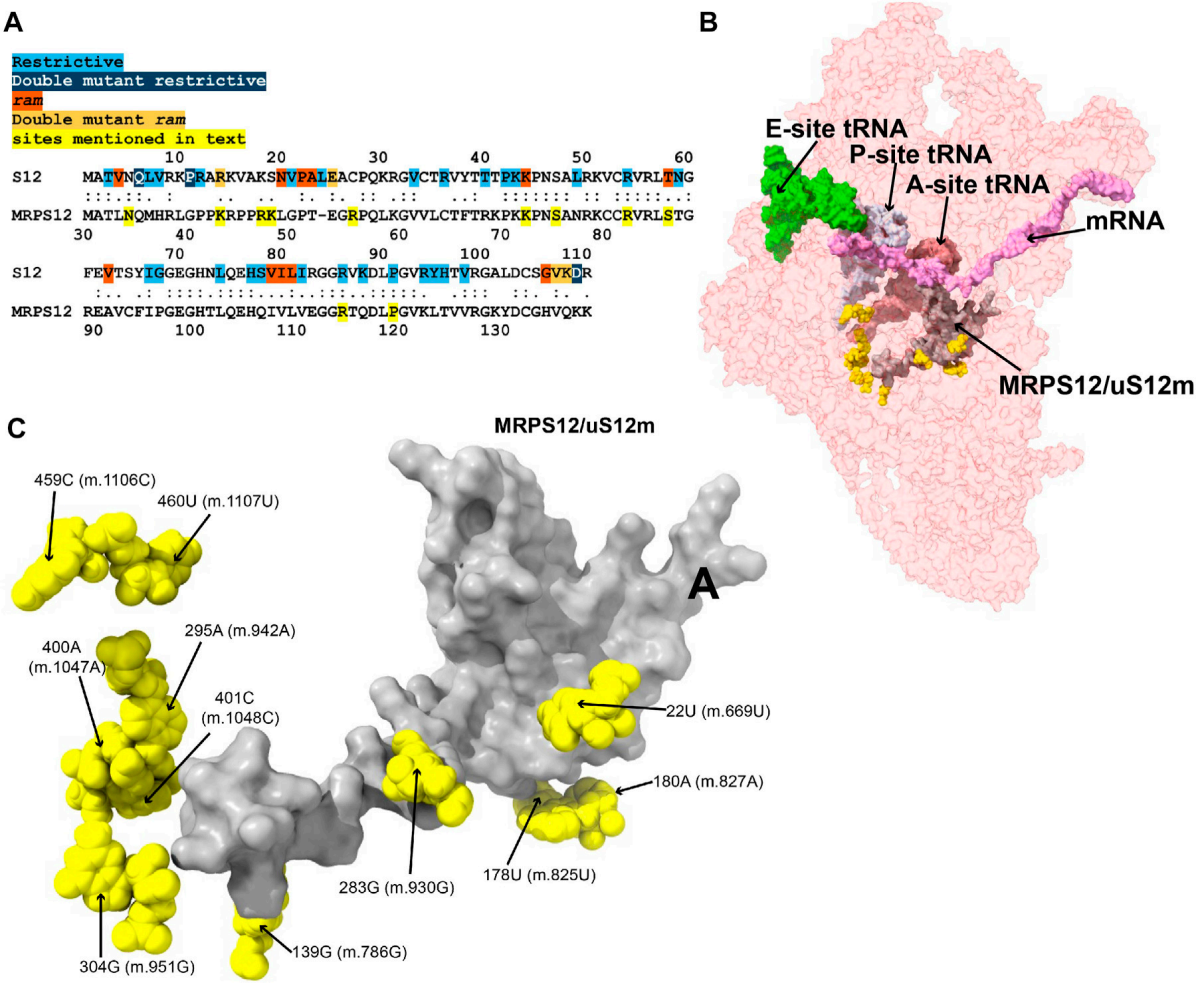
Variants in the neighborhood of MRPS12/uS12m. **(A)** Alignment between the *E. coli* and human mitochondrial versions of ribosomal protein S12. Sites of S12 fidelity mutations are color coded as indicated in color key above the alignment (Gregory et al., 2001; Agarwal et al., 2011; Datta et al., 2021). Note that the bacterial numbering is offset by one residue relative to the aforementioned references due to the inclusion of an M at position 1. **(B)** Sites of potential deafness-associated variations (yellow) in the neighborhood of MRPS12/uS12m (gray) are shown in the context of the mt-SSU (semi-transparent, red surface). The following mito-ribosomal ligands are shown to provide perspective: A- (dark pink), P- (light gray), and E-site (light green) mt-tRNAs, and mRNA (pink). **(C)** Blown-up version of B showing the 11 sites of variation (yellow) in the neighborhood of MRPS12/uS12m (gray), with their positions indicated.

MRPS12/uS12m is an early-binding mito-ribosomal protein that is structurally almost identical to its bacterial counterpart, ribosomal protein S12 (Amunts et al., 2015, Lopez; Greber et al., 2015; Sanchez, Maria Isabel G. et al., 2021). For a thorough review of the role of S12 in the maintenance of decoding fidelity during protein synthesis, we refer the reader to our accompanying study (Vila-Sanjurjo et al., 2023). For the scope of this work, we will mention that the bacterial S12 has been shown to contain many sites, sparsely distributed over its primary sequence, where mutations give rise to fidelity phenotypes. These fidelity mutations can act by increasing the error rate during decoding at the A site (ram or ribosomal ambiguity mutations) or decreasing the error rate (hyper-accurate mutations) (Gregory et al., 2001; Agarwal et al., 2011; Datta et al., 2021). The primary sequence alignment in Figure 2A attests to the high homology between *Escherichia coli* S12 and mitochondrial MRPS12/uS12m (see also Figure 2 in our accompanying study; Vila-Sanjurjo et al., 2023). The structural analysis places MRPS12/uS12m near the A site of the ribosome, being the only protein capable of directly participating in decoding (Carter et al., 2000; Amunts et al., 2015; Greber et al., 2015). Given the implication of S12 in the maintenance of genetic decoding during protein synthesis, we paid particular attention to the eleven 12S mt-rRNA variants lying in close proximity to its human mitochondrial ortholog (code: M in Supplementary Table S1 and Figures 2B,C).

##### −22U>C (m.669U>C) and 283G>A (m.930G>A)

**FIGURE 3:**
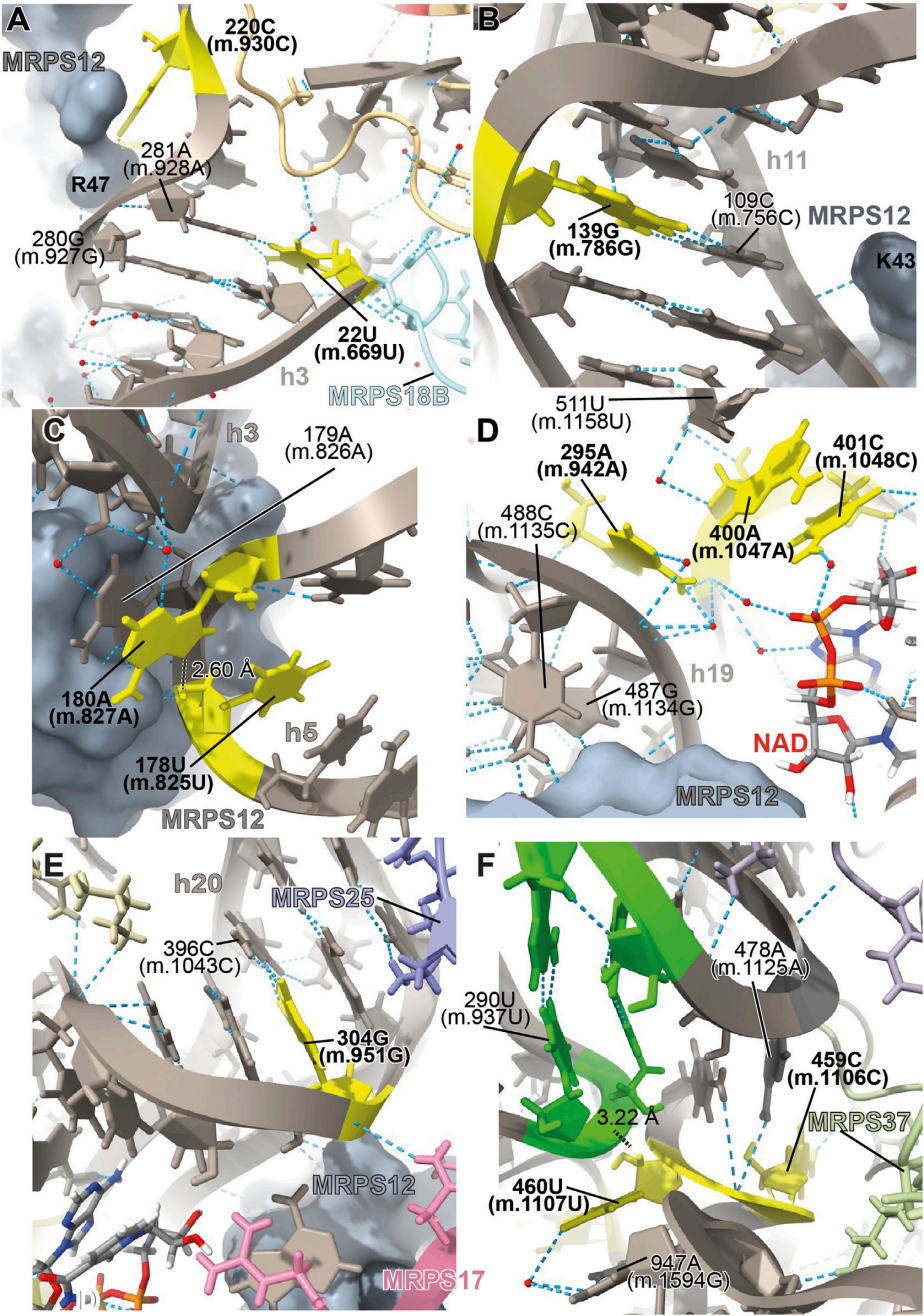
Structural inspection of the variants in the neighborhood of MRPS12/uS12m. **(A–F)** Annotated view of the regions containing the following variants: 22U>C (m.669U>C) and 283G>A (m.930G>A) **(A)**; 139G>A (m.786G>A) **(B)**; 178U>A (m.825U>A) and 180A>G (m.827A>G) **(C)**; 295A>G (m.942A>G), 400A>G (m.1047A>G), and 401C>U (m. 1048C>U) **(D)**; 304G>A (m.951G>A) **(E)**; and 459C>U (m.1106C>U) and 460U>C (m.1107U>C) **(F)**. Variants are shown in yellow and labeled in bold, black font. Other rRNA residues are labeled in regular font. 12S mt-rRNA is shown in gray with helix numbers indicated. Central domain pseudoknot connecting helices h19 and h25, green. MRPS12/uS12m is shown as a grey surface in **(A–E)**. Other components of the mito-ribosome are labeled and color coded to their molecular model. Water molecules, red spheres; hydrogen bonds, blue, dashed lines; distances, black, dashed lines with distances indicated.

The **22U>C (m.669U>C)** variant has been detected in several studies targeting hearing-impaired patients (Leveque et al., 2007; Elstner et al., 2008; Rydzanicz et al., 2009; Rydzanicz et al., 2010). Position **22U (m.669U)** base pairs with 281A (m.928A) in h3 of the SSU (Figure 3A and Supplementary Figure S1A). A hydrogen bond between the RNA backbone at the adjacent position 280G (m.927G) and Arg 47 of MRPS12/uS12m is visible in the structure (Figure 3A), in close proximity to known sites of fidelity mutations in the bacterial protein (Figure 2A). Positions 21–23 (m.668–670) pack against protein MRPS18B/bS18b (cyan in Figure 3A and Supplementary Figure S1D), with two hydrogen bonds established between the protein and the rRNA backbone at positions 21 (m.668) and 23 (m.670). Protein MRPS5/uS5m (beige in Figure 3A and Supplementary Figure S1D) is also in the vicinity of these residues. The 22U:281A (m.669U:m.928A) base pair stabilizes the junction between helices h1 and h3. A contact between residue V336 of MRPS5/uS5m (red in Supplementary Figure S1C), one of the two known *ram* mutations in mammalian mitochondria, and the backbone between positions 3 (m.650) and 4 (m.651) of h1 is visible (Akbergenov et al., 2018). As the **22U>C (m.669U>C)** variant would replace a Watson:Crick U:A base pair with a C•A mismatch at the junction between helices h1 and h3 and in an rRNA region that is surrounded by proteins MRPS12/uS12m and MRPS5/uS5m, the potential for a fidelity mutation is high.

The haplotype marker **283G>A (m.930G>A)** has been found to be associated with deafness in three studies (Konings et al., 2008; Rydzanicz et al., 2010; Guaran et al., 2013). In the secondary structure map of 12S mt-rRNA, position **283G (m.930G)** lies in the single-stranded stretch linking h3 and the functionally important central pseudoknot (helices h1 and h2) to h19 (Supplementary Figure S1A) (Brink et al., 1993; Poot et al., 1996; Poot et al., 1998). In the 2.2-Å mito-ribosomal structure (Itoh et al., 2021; Itoh et al., 2022), the O6 of **283G (m.930G)** has been modeled 2.39 Å away from Arg 47 of MRPS12/uS12m (Figure 3A and Supplementary Figure S1A), whose equivalent position in the bacterial ribosome would be near sites of fidelity mutations (Figure 2A). However, the two residues are likely quite disordered, as judged by the lack of clear density covering them (Supplementary Figure S1C). Despite this, the proximity of position **283G (m.930G)** to MRPS12/uS12m makes the **283G>A (m.930G>A)** variant constitute a good candidate for a pathogenic residue. However, in light of its abundance in the population (1,045 appearances in GenBank, Supplementary Table S1) (Ruiz-Pesini et al., 2007; Konings et al., 2008; Rydzanicz et al., 2010), the defect caused by this variant must be small, albeit possibly sufficient to cause a fidelity phenotype.

##### −139G>A (m.786G>A)

The **139G>A (m.786G>A)** rare variant was detected by Guaran et al. (2013). This residue is base-paired to 109C (m.756C) in helix h11 (Figure 3B and Supplementary Figure S2A). A hydrogen bond is visible between Lys 43 of MRPS12/uS12m, a heterologous equivalent of a fidelity mutation in bacteria (Figure 2A) (Agarwal et al., 2011) and the RNA backbone at position 109C (m.756C) (Figure 3B and Supplementary Figure S2D). The G>A base change would replace a wild-type C:G base pair with a C•A mismatch in the vicinity of MRPS12/uS12m. Thus, the potential for a fidelity mutation is high, a conclusion that is in strong agreement with the patient and familial diagnosis performed by the authors (Guaran et al., 2013).

##### −178U>A (m.825U>A) and 180A>G (m.827A>G)

The variants **178U>A (m.825U>A)** and **180A>G (m.827A>G)** are both haplotype markers that were found in association with deafness in several studies (Li et al., 2004; Li et al., 2005; Xing et al., 2006; Chaig et al., 2008; Konings et al., 2008; Human et al., 2010; Rydzanicz et al., 2010; Yano et al., 2014). In the 12S mt-rRNA secondary structure, both **178U (m.825U)** and **180A (m.827A)** are located in the single-stranded RNA stretch linking helices h5 and h15 (Supplementary Figure S3A), a region that is in close proximity of MRPS12/uS12m (Figure 3C). Several hydrogen bonds stabilize the quaternary structure in this region. First, MRPS12/uS12m Arg 55 is within hydrogen bonding distance of the base of 179A (m.826A) (Supplementary Figure S3A). Additionally, Arg 55, together with the side chain of Ser 86, establishes hydrogen bonds with the RNA backbone at positions **178U (m.825U)** and 179A (m.826A) (Supplementary Figure S3C). These MRPS12/uS12m residues are either the heterologous equivalent (Ser 86) or adjacent to a heterologous equivalent (Arg 55) of bacterial fidelity mutations (Figure 2A) (Agarwal et al., 2011). Notably, a conserved tertiary interaction is established between the 2′-hydroxyl of **178U (m.825U)** and the N7 of **180A (m.827A)** (Figure 3C and Supplementary Figure S3C). The base at position 180A (m.827A) stacks onto that of the adjacent 179A (m826A), which forms an A-minor interaction with the distant helix h3, at position 27U (m.674U) (Supplementary Figure S3A, 3C), thus physically connecting helices h3 and h11 (Nissen et al., 2001). Position 180A (m.827A) itself contributes to this stabilization via a water-mediated hydrogen bond (Figure 3C). Since the interaction between h11 and h3 in this region forms an important contact surface between 12S mt-rRNA and MRPS12/uS12m, its overall structure is thought to be important for mito-ribosomal function. Additionally, this region is very close to the binding site for mitochondrial elongation factor G1 (EFG1) (Supplementary Figure S3E), as judged from a structure of the human mitochondrial ribosome bound to this factor (Koripella et al., 2020). Specifically, the rRNA backbone at position **178U (m.825U)** is located ∼3 Å away from the factor, indicating that this region might have an important role during translocation. In addition to this, **180A (m.827A)** is an almost universally conserved residue (position 364 in *E. coli* 16S rRNA) (Cannone et al., 2002), although the reason for this conservation might not be completely obvious, considering its status as a haplotype marker. Even though an A>G variant can be tolerated at this position, the introduction of a bulky NH_2_ group at position N3 due to the **180A>G (m.827A>G)** base change might result in a slight structural distortion of the region, perhaps by interfering with its interaction with helix h3. A similar structural argument could be made for the **178U>A (m.825U>A)** variant in that the insertion of a bulkier adenosine at this position could also slightly induce the distortion of the local structure. Hence, the potential for a fidelity variant exists in both cases.

##### −295A>G (m.942A>G), 400A>G (m.1047A>G), and 401C>U (m. 1048C>U)

The **295A>G (m.942A>G)** variant was found in two patients with hearing loss (Lu et al., 2010; Guaran et al., 2013). In the secondary structure of 12S mt-rRNA, position **295A (m.942A)** is located at the 5′ end of h20 (Supplementary Figure S4A). Two more deafness-associated sites of variation map to the neighborhood of **295A>G (m.942A>G)**, namely, **400A (m.1047A)** and **401C (m.1048C)**, where an A>G and a C>U transition were, respectively, identified in patients with hearing loss (Konings et al., 2008; Rydzanicz et al., 2010). In the secondary structure map of the SSU rRNA, these two residues are located in the unpaired stretch separating the two base-paired segments of h20 (Figure 3D and Supplementary Figure S4A). The conformation of this region of the SSU has been substantially remodeled in the new 2.2-Å structure of the mito-ribosome due to the new assignment of density to two somewhat unexpected mito-ribosomal ligands that were clearly identifiable at this resolution, a molecule of NAD (Figure 3D) and a polyamine molecule modeled as spermine (not shown) (Itoh et al., 2021; Itoh et al., 2022). In fact, the N4 of **401C (m.1048C)** is modeled as part of a water-mediated, hydrogen bonding interaction with one of the phosphate oxygens of NAD (Figure 3D). Additional contacts to these ligands are a hydrogen bond from **401C (m.1048C)** O2′ to NAD O2′ and a hydrogen bond between spermine N1 and the RNA backbone at position **400A (m.1047A)** (not shown). **400A (m.1047A)** is unpaired and its base stacks onto that of **401C (m.1048C)** (Figure 3D). A hydrogen bond was detected between the N6 of **295A (m.942A)** and **400A (m.1047A)** OP1 (Figure 3D). The same moiety of **295A (m.942A)** also forms water-mediated hydrogen bonds with the backbone of h19 at positions 487–88 (m.1134–35). Notably, position 487G (m.1134G) is within 3 Å of MRPS12/uS12m (Figure 3D). Adjacent to the variation site **295A (m.942A**) is the Watson:Crick base pairing formed by positions 294G (m.941G) and 486C (m.1133C) (indicated with a thick, black line in Supplementary Figure S4A), which further strengthens the structural connection between helices h19 and h20, thus bringing the proximal end of h20 in close proximity to protein MRPS12/uS12m.

Of the three variant bases, only **295 (m.942)** is involved in the stabilization of mito-ribosomal structure via direct (and water-mediated) hydrogen bonds, namely, in the interaction between helices h19, h20 in the neighborhood of protein MRPS12/uS12m. Guaran et al. found the **295A>G (m.942A>G)** variant together with the 35delG/35delG allele in the gene GJB2, encoding the protein connexin 26, which is the most common cause of genetic sensorineural deafness in Caucasian patients (Estivill et al., 1998b; Guaran et al., 2013). Hence, the potential contribution of the **295A>G (m.942A>G)** variant to the observed symptoms is unclear in this patient. No GJB2 mutations were reported together with **295A>G (m.942A>G)** in the other study (Lu et al., 2010). As for the **400A>G (m.1047A>G)** and **401C>U (m. 1048C>U)** variants, the situation is uncertain. The base of **401C (m.1048C)** only interacts via a water-mediated hydrogen bond with NAD (Figure 3D). Whether the water-mediated interaction between **401C (m.1048C)** and NAD is of any relevance to mitochondrial translation is unknown. The abundance of the **401C>U (m. 1048C>U)** variant, itself a haplotype marker, clearly indicates that the C>U base change is well-tolerated at this position. In the case of **400A (m.1047A)**, its base is not involved in any hydrogen-bonding interaction, prompting us to regard it as a silent polymorphism. Despite all this, due to the proximity of **401C (m.1048C)** and **400A (m.1047A)** to MRPS12, a potentially pathogenic role cannot be completely ruled out for these residues.

##### −304G>A (m.951G>A)

The **304G>A (m.951G>A)** variant is a haplotype marker that is associated with deafness in several studies (Elstner et al., 2008; Konings et al., 2008; Lu et al., 2010; Rydzanicz et al., 2010; Guaran et al., 2013; Igumnova et al., 2019). Position **304G (m.951G)** is base-paired to 396C (m.1043C) in the distal part of h20 (Figure 3E and Supplementary Figure S5A), a region that is very well resolved in the structure. The backbone at position 304G (m.951G) packs against MRPS12/uS12 (less than 4 Å away from Asn 34) (Figure 3E), near the location of fidelity mutations in bacteria (Figure 2A) (Agarwal et al., 2011). Additionally, a hydrogen bond is formed between Asn 44 of MRPS17/uS17m (pink in Figure 3E and Supplementary Figure S5C) and the RNA backbone at position 304G (m.951G). Additional RNA:protein contacts involving MRPS25/mS25 (light purple in Figure 3E and Supplementary Figure S5C) are also visible in the neighborhood of **304G (m.951G)**. As the G>A base change at position **304 (m.951)** would disrupt a base pair interaction next to sites of MRPS12/uS12 that are heterologous equivalents of known fidelity mutations, the potential of the variant to cause a fidelity phenotype is considered high.

##### −459C>U (m.1106C>U) and 460U>C (m.1107U>C)

The **459C>U (m.1106C>U)** variant was found in heteroplasmy in a patient with non-syndromic hearing loss while the haplotype marker **460U>C (m.1107U>C)** was identified as a potential deafness-causing variant in three subjects from a cohort of aminoglycoside-induced and non-syndromic hearing-impaired Chinese pediatric patients (Li et al., 2005; Konings et al., 2008). In the secondary structure map of 12S mt-rRNA, these residues map to the single-stranded stretch between helices h24 and h25 in the central domain (Supplementary Figure S6A). The base of **459C (m.1106C)** displays a potential contact with the backbone of protein MRPS37/mS37 (Figure 3F), which has been implicated in restricting the rotation of the SSU during initiation (Khawaja et al., 2020). Although this contact is thought to be lost in mito-ribosomes carrying the **459C>U (m.1106C>U)** variant, the results of this loss are uncertain.

A tertiary interaction is observed between the backbone at position **460U (m.1107U)** with the N6 of 478A (m.1125A), two nucleotides away from the central domain pseudoknot that connects helices h19 and h25 of 12S mt-rRNA (shown in red in Supplementary Figure S6A; green residues in Figure 3F) (Vila et al., 1994). Additionally, the distance from **460U (m.1107U)** O2′ to 290U (m.937U) OP1 is 3.217 Å, possibly indicating a direct tertiary interaction with the central domain pseudoknot (Figure 3F). Mutations at the central domain pseudoknot have been made in *E. coli* and were shown to be deleterious (Vila et al., 1994). Despite this, the base of **460U (m.1107U)** is not involved in any direct interactions with other residues except for a water-mediated hydrogen bond with position 947A (m.1594G) near the 3′end of the molecule (Figure 3F and Supplementary Figure S6D). The importance of water-mediated hydrogen bonds in the maintenance of the local structure is difficult to evaluate. However, the fact that the *S. scrofa* mito-ribosome contains a C at a position equivalent to **460U (m.1107U)** suggests that the **460U>C (m.1107U>C)** variant could be a simple polymorphism (but see below).

Considering all of this, as the 5’ side of the central domain pseudoknot is adjacent to h19, which, in turn, wraps around MRPS12/uS12m, it is possible that even small distortions in this region could be transmitted through this protein and result in fidelity defects. As a result, the potential for the creation of slight fidelity phenotypes by the variants **459C>U (m.1106C>U)** and **460U>C (m.1107U>C)** cannot be ruled out.

#### Variants in the proximity of MRPs involved in the assembling of the 5’ domain of 12S mt-rRNA

**FIGURE 4:**
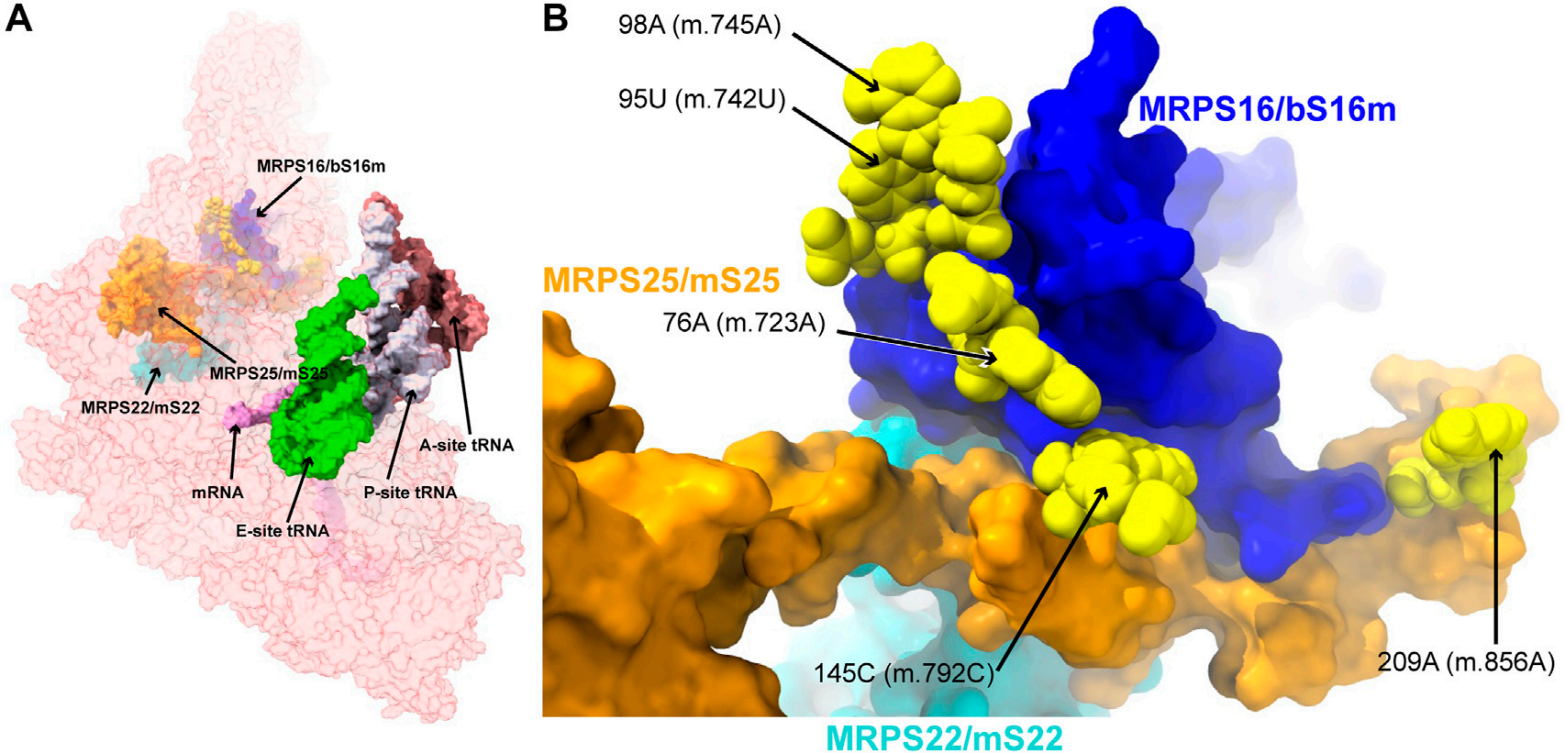
Variants in the proximity of the MRPs involved in the assembling of the 5′ domain of 12S mt-rRNA. **(A)** Sites of potential deafness-associated variations (yellow) in the neighborhood of MRPS16/bS16m (blue), MRPS22/mS22 (cyan), and MRPS25/mS25 (orange), shown in the context of the mt-SSU (semi-transparent, red surface). Mito-ribosomal ligands color coded as in Figure 2B. **(B)** Blown-up version of **(A)** showing the 5 sites of variation associated to this region of the mt-SSU (yellow), with their positions indicated. Mito-ribosomal proteins color-coded as in **(A)**.

During the early assembly, mito-ribosomal proteins MRPS16/bS16m and MRPS18B/mS40 interact with the nascent 12S mt-rRNA transcript (5′-domain, Figure 1A), forming the lower body of the mt-SSU (Bogenhagen et al., 2018). MRPS22/mS22 is also part of this group of early-binding proteins, albeit without direct contact with 12S mt-rRNA (Bogenhagen et al., 2018). Late-assembly proteins MRPS15/uS15m, MRPS25/mS25, and MRPS26/mS26 bind to the SSU body near MRPS16/bS16m and MRPS22/mS22 (Bogenhagen et al., 2018). Deleterious mutations have been identified in human MRPS16/bS16, MRPS22/mS22, and MRPS25/mS25 (Miller et al., 2004; Emdadul Haque et al., 2008; Smits et al., 2011; Bugiardini et al., 2019; Huang et al., 2020; Sanchez et al., 2021). Subsequently, we analyzed five variants potentially affecting the binding of these three proteins (Figure 4).

##### −76A>C (m.723A>C) and 145C>U (m.792C>U)

**FIGURE 5:**
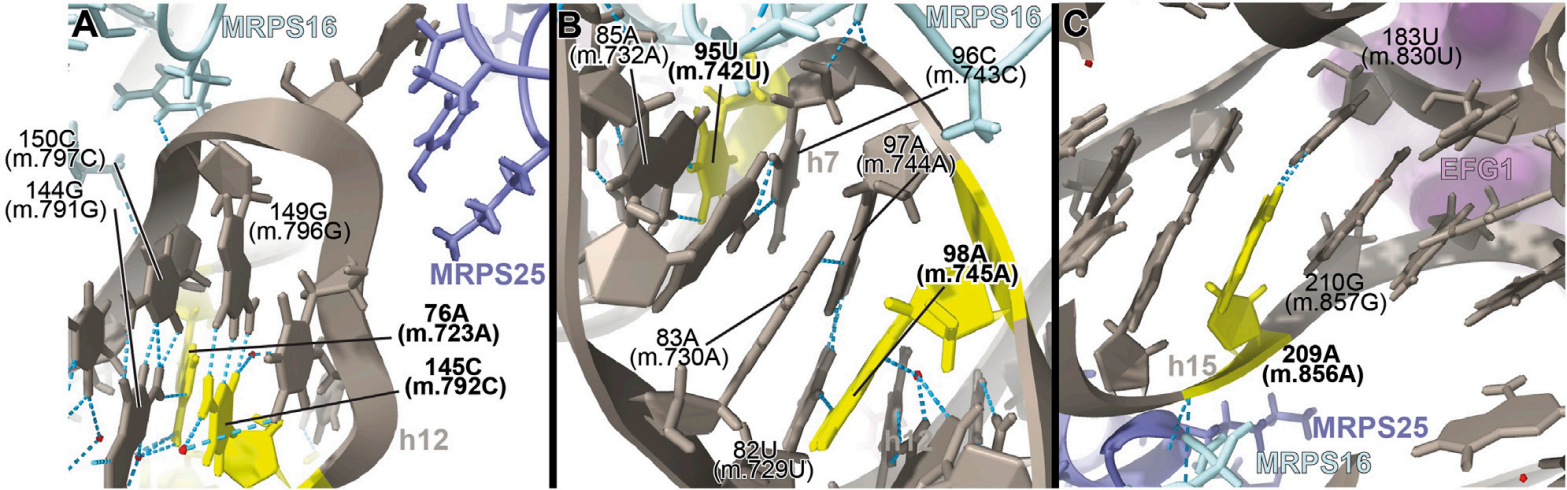
Structural inspection of the variants in the proximity of MRPs involved in the assembling of the 5′ domain of 12S mt-rRNA. **(A–C)** Annotated view of the regions containing the following variants: 76A (m.723A) and 145C (m.792C) **(A)**; 95U (m.742U) and 98A (m.745A) **(B)**; and 209A (m.856A) **(C)**. Color coding and labeling as in Figure 3.

The **76A>C (m.723A>C)** base change was found in two patients with non-syndromic hearing loss (Konings et al., 2008; Rydzanicz et al., 2010). Position **76A (m.723A)** is involved in a triple base interaction with the 144G:150C (m.791G:797C) base pair, thus bringing together helices h7 and h12 (Supplementary Figure S7A). A second variant was found adjacent to 144G (m.791G) at position **145C (m.792C)** of h12, where a C>U base change was identified in two patients with non-syndromic hearing loss (Lu et al., 2010; Yano et al., 2014). Position **145C (m.792C)** is canonically base-paired to 149G (m.796G), closing the terminal loop of h12 (Figure 5A and Supplementary Figure S7A).

The interactions established by both residues contribute to the creation of a structural motif that is recognized by proteins MRPS16/bS16 and MRPS25/mS25 (Figure 5A). In the case of the **76A>C (m.723A>C)** variant, the base change would prevent the base triple interaction, while the C>U base change at position **145C (m.792C)** would result in a structurally disfavored C:G/U•G exchange at a terminal base pair (Ananth et al., 2013). Given the location of both residues near the recognition sites for MRPS16/bS16 and MRPS25/mS25, the expected disruption of the secondary and tertiary structure of 12S mt-rRNA brought about by the variants could also result in quaternary structure defects affecting ribosomal proteins known to harbor pathogenic mutations.

##### −95U>C (m.742U>C) and 98A>G (m.745A>G)

The **95U>C (m.742U>C)** and the **98A>G (m.745A>G)** variants were identified in a patient with idiopathic, sensorineural hearing loss and a patient with profound hearing impairment, respectively (Lu et al., 2010; Guaran et al., 2013). Position **95U (m.742U)** base-pairs to 85A (m.732A) in h7 (Figure 5B and Supplementary Figure S8A). Position **98A (m.745A)** is involved in a base quadruple interaction involving the 82U:97A (m.729U:744A) base pair and position 83A (m.730A), both of them in h7 (Figure 5B and Supplementary Figure S8A). Although this region of h7 is located toward the back of the subunit, which, in principle, argues against a disruptive phenotype for the variants, it is in close contact with protein MRPS16/bS16 (pale blue in Figure 5B), known to harbor pathogenic mutations. Indeed, MRPS16/bS16 makes two contacts to the rRNA backbone at position 96C (m.743C) (Figure 5B). Given the fact that both **95U (m.742U)** and **98A (m.745A)** are structurally important in the context of h7 and that this helix is part of the MRPS16/bS16 binding site, a disruptive effect beyond the local structure of h7 cannot be ruled out.

##### −209A>G (m.856A>G)

The **209A>G (m.856A>G)** variant was identified in four patients with non-syndromic hearing loss (Lu et al., 2010; Yano et al., 2014). The variant was also identified as potentially associated with Alzheimer’s (Tanaka et al., 2010) and Leber’s diseases (Sawano et al., 1996). Position **209A (m.856A)** is base-paired to 183U (m.830U) at the proximal end of helix h15 (Figure 5C and Supplementary Figure S9A). Hydrogen bonds from MRPS16/bS16 (light green in the structure) and MRPS25/mS25 (light purple in the structure) to the RNA backbone at position 209A (m.856A) are visible in the structure (Figure 5C). In addition, the distance to EFG1 measured from the adjacent 210G (m.857G) is ∼5Å (Figure 5C). The 209A>G (m.856A>G) variant would result in the substitution of an A:U Watson:Crick by a G•U wobble. Given the structural and functional importance of the region, the potential for a deleterious effect associated with the slight distortion introduced by the **209A>G (m.856A>G)** variant is considerable.

#### Variants in the proximity of the MRPs involved in the assembling of the 3’ domain of 12S mt-rRNA

**FIGURE 6:**
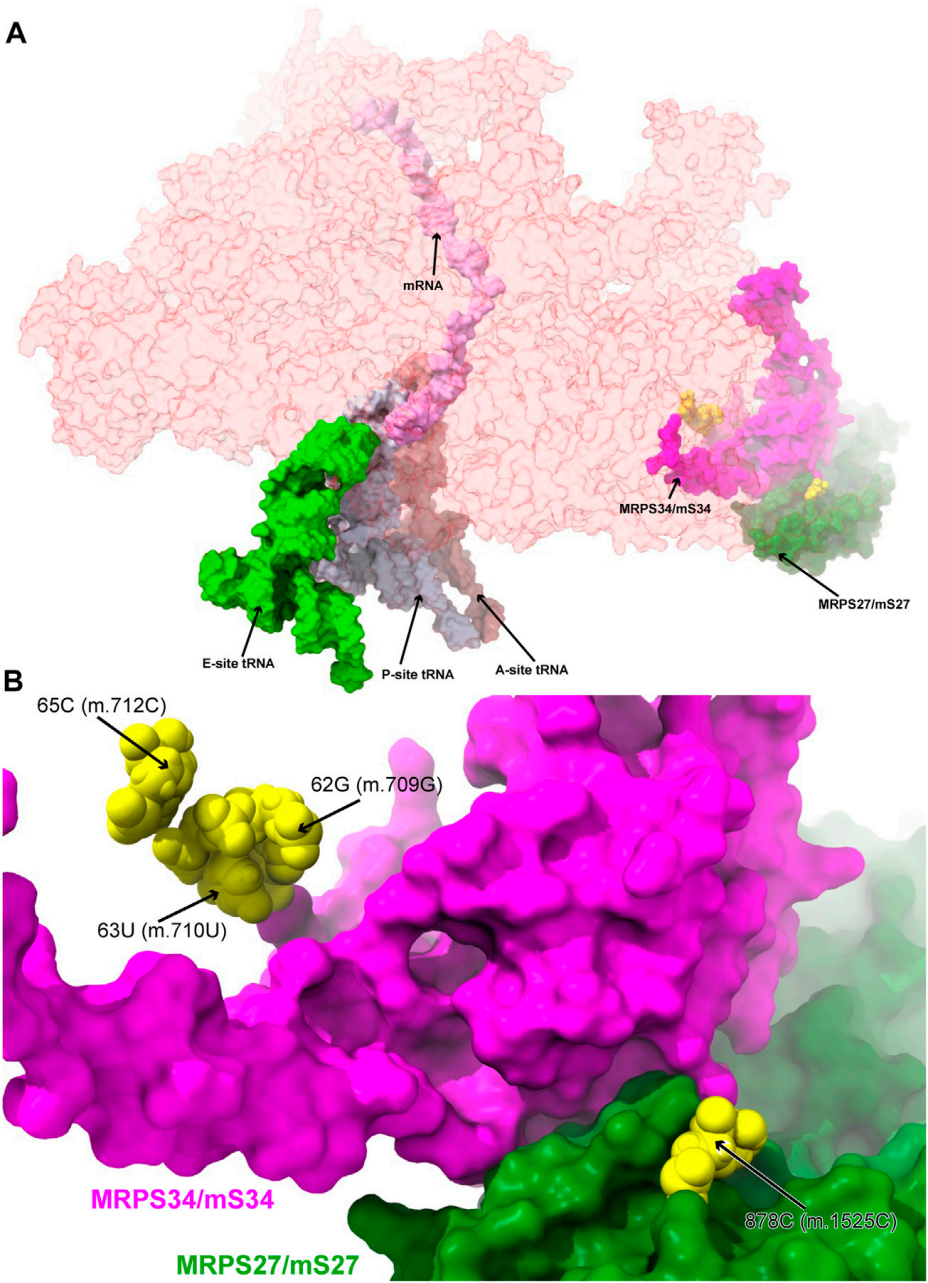
Variants in the proximity of the MRPs involved in the assembling of the 3′ domain of 12S mt-rRNA. **(A)** Sites of potential deafness-associated variations (yellow) in the neighborhood of MRPS27/mS27 (green) and MRPS34/mS34 (magenta), shown in the context of the mt-SSU (semi-transparent, red surface). Mito-ribosomal ligands color coded as in Figure 2B. **(B)** Blown-up version of **(A)** showing the 4 sites of variation associated to this region of the mt-SSU (yellow), with their positions indicated. Mito-ribosomal proteins color coded as in **(A)**.

During assembly, the 5′ and 3′ domains are brought together by the strong interactions established by the 5′-domain binders MRPS16/bS16m, MRPS18B/mS40, and MRPS22/mS22 and the 3′-domain binders MRPS27/mS27 and MRPS34/mS34 (Bogenhagen et al., 2018). The important role of protein MRPS27/mS27 is further underscored by its involvement in LSU assembly and in the formation of inter-subunit bridge mB6 (Kim and Barrientos, 2018). In addition, pathogenic mutations in MRPS34/mS34 have been identified (Wang et al., 2021). Then, we analyzed four variants potentially affecting the binding of MRPS27/mS27 and MRPS34/mS34 (Figure 6).

##### −62G>A (m.709G>A), 63U>C (m.710U>C), and 65C>A (m.712C>A)

**FIGURE 7:**
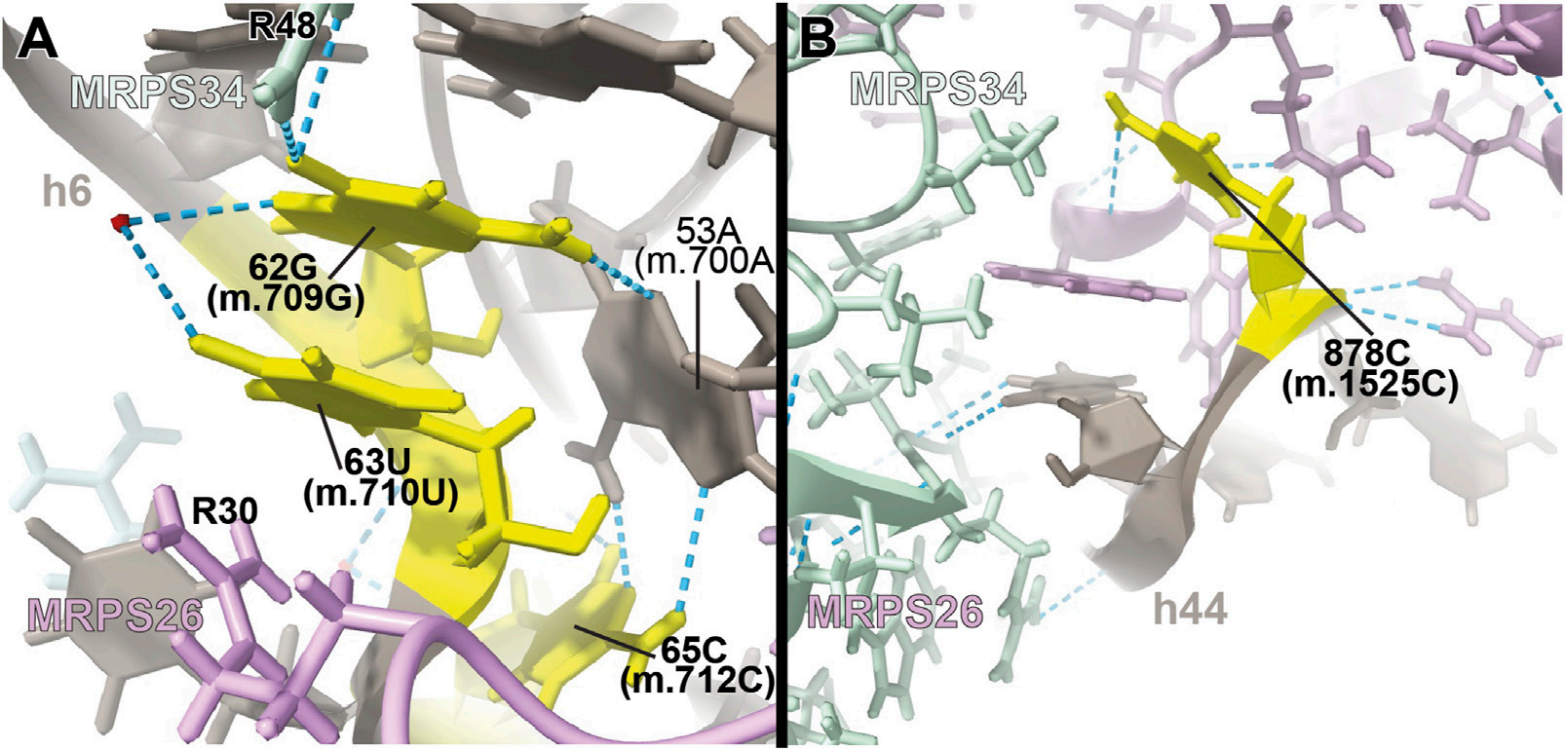
Structural inspection of the variants in the proximity of MRPs involved in the assembling of the 3′ domain of 12S mt-rRNA. **(A,B)** Annotated view of the regions containing the following variants: 62G>A (m.709G>A), 63U>C (m.710U>C), and 65C>A (m.712C>A) (A) and 878C>G (m.1525C>G) **(B)**. Color coding and labeling as in Figure 3.

Three deafness-associated variants have been identified in the single-stranded stretch connecting helices h6 and h6a in the secondary structure map of the mt-SSU, namely, the haplotype defining variant **62G>A (m.709G>A)**, which has been frequently associated with hearing impairment (Li et al., 2005; Elstner et al., 2008; Konings et al., 2008; Rydzanicz et al., 2010; Guaran et al., 2013), as well as the **63U>C (m.710U>C)** and **65C>A (m.712C>A)** variants (Figure 7A and Supplementary Figure S10A) (Konings et al., 2008; Guaran et al., 2013). The base of **62G (m.709G)** is modeled, making two hydrogen bonds to Arg 48 of MRPS34/mS34 (pale green in Figure Fig7A and Supplementary Figure S10D) and one to the N3 of 53A (m.700A), which, in turn, positions its Hoogsteen face within a hydrogen bonding distance of 65C (m.712C) (Figure Fig7A). The quaternary interaction involving MRPS34/mS34 and the base triple formed between **62G (m.709G)**, 53A (m.700A), and **65C (m.712C)** stabilizes the structure of the RNA stretch separating helices h6 and h6a and provides a recognition surface for the late-binding protein MRPS26/mS26 (pink in Figure 7A) (Bogenhagen et al., 2018). The abundance of the haplotype marker **62G>A (m.709G>A)** clearly implies that the variant must be easily accommodated in the SSU structure. Despite this, the **G>A** base change is expected to disrupt the hydrogen bonding network observed in the structure, involving tertiary and quaternary contacts and likely leading to some degree of distortion. Similarly, the pyrimidine-to-purine replacement caused by the **65C>A (m.712C>A)** variant is expected to disrupt the non-canonical base pair between **65C (m.712C)** and 53A (m.700A). With this in mind, both variants must be regarded as potentially pathogenic.

Adjacent to **62G (m.709G)** is **63U (m.710U)**, whose base is stacked between **62G (m.709G)** and Arg 30 of MRPS26/mS26 (Figure 7A). **63U (m.710U)** is involved in a water-mediated hydrogen bonding interaction with **62G (m.709G)** N7 (Figure 7A). Despite this, the **U>C** base change at position **63 (m.710)** is not expected to bring about any disruptive effect, as shown by the fact that a C is found at the equivalent position in the *S. scrofa* mito-ribosome (not shown) (Greber et al., 2015).

##### −878C>G (m.1525C>G)

The highly rare **878C>G (m.1525C>G)** variation was found by our group in a patient with profound hearing loss (Smith et al., 2014). This variant was described as part of our original HIA work, albeit without a meaningful diagnostic due to its low conservation (Smith et al., 2014). Position **878C (m.1525C)** is located at the very distal end of h44 (Supplementary Figure S11A), which serves as a recognition site for mito-ribosomal proteins MRPS27/mS27 (dark pink in Figure 7B) and MRPS34/mS34 (pale green in Figure 7B). The quality of the density of this peripheral region of the mito-ribosome is low, probably reflecting some degree of disorder in the structure. Despite this, the base of **878C (m.1525C)** has been modeled, making a hydrogen bond to the backbone of protein MRPS27/mS27 (Figure 7B), which would be lost in the variant. Although the distal location of **878C (m.1525C)** would, in principle, argue for a silent phenotype, the rarity of the C>G variant, together with the existence of pathogenic mutations in MRPS34/mS34 (Wang et al., 2021), possibly indicates the existence of strong selective pressure acting at this position.

#### Variants in the proximity of MRPS7/uS7m and MRPS14/uS14m

**FIGURE 8:**
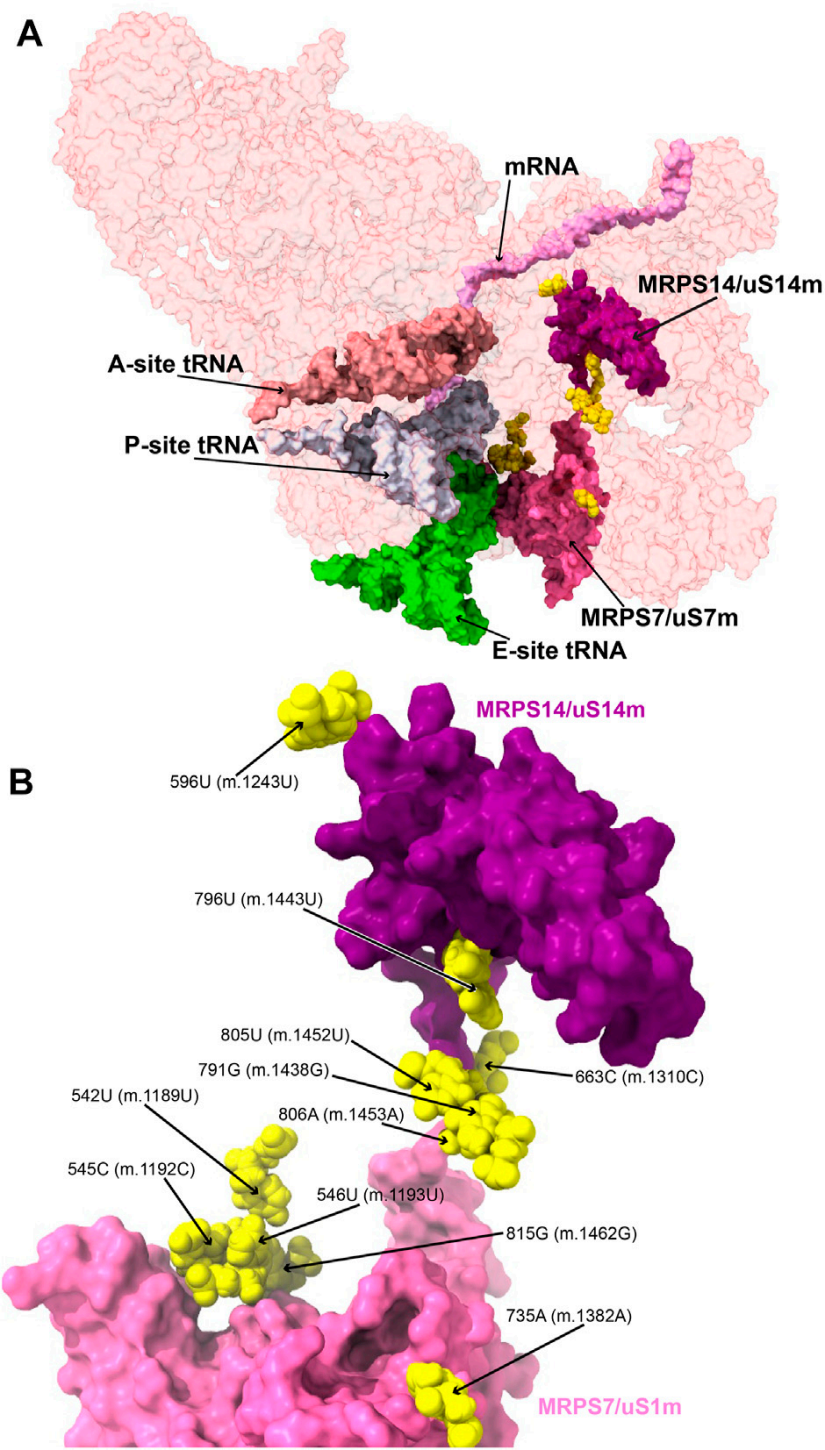
Variants in the proximity of MRPS7/uS7m and MRPS14/uS14m. **(A)** Sites of potential deafness-associated variations (yellow) in the neighborhood of MRPS7/uS7m (pink) and MRPS14/uS14 (purple), shown in the context of the mt-SSU (semi-transparent, red surface). Mito-ribosomal ligands color coded as in Figure 2B. **(B)** Blown-up version of A showing the 11 sites of variation associated with this region of the mt-SSU (yellow), with their positions indicated. Mito-ribosomal proteins color coded as in **(A)**.

MRPS7/uS7m is an early binding protein, which, together with MRPS9/uS9m and MRPS29/mS29, recognizes the major 3’ domain of 12S rRNA (De Silva et al., 2015; Bogenhagen et al., 2018; Sanchez et al., 2021). The binding of these proteins creates binding sites for late proteins MRPS14/uS14m, MRPS10/uS10m, MRPS24/uS3m, and MRPS33/mS33 (De Silva et al., 2015; Bogenhagen et al., 2018; Sanchez et al., 2021). Pathogenic mutations have been found in MRPS7/uS7m and MRPS14/uS14m (Menezes et al., 2015; Jackson et al., 2019). Then, we analyzed the potential role of eleven variants mapping to the neighborhood of MRPS7/uS7m and MRPS14/uS14m (Figure 8).

##### −542U>C (m.1189U>C), 545C>A (m.1192C>A), 546U>C (m.1193U>C), and 815G>A (m.1462G>A)

**FIGURE 9:**
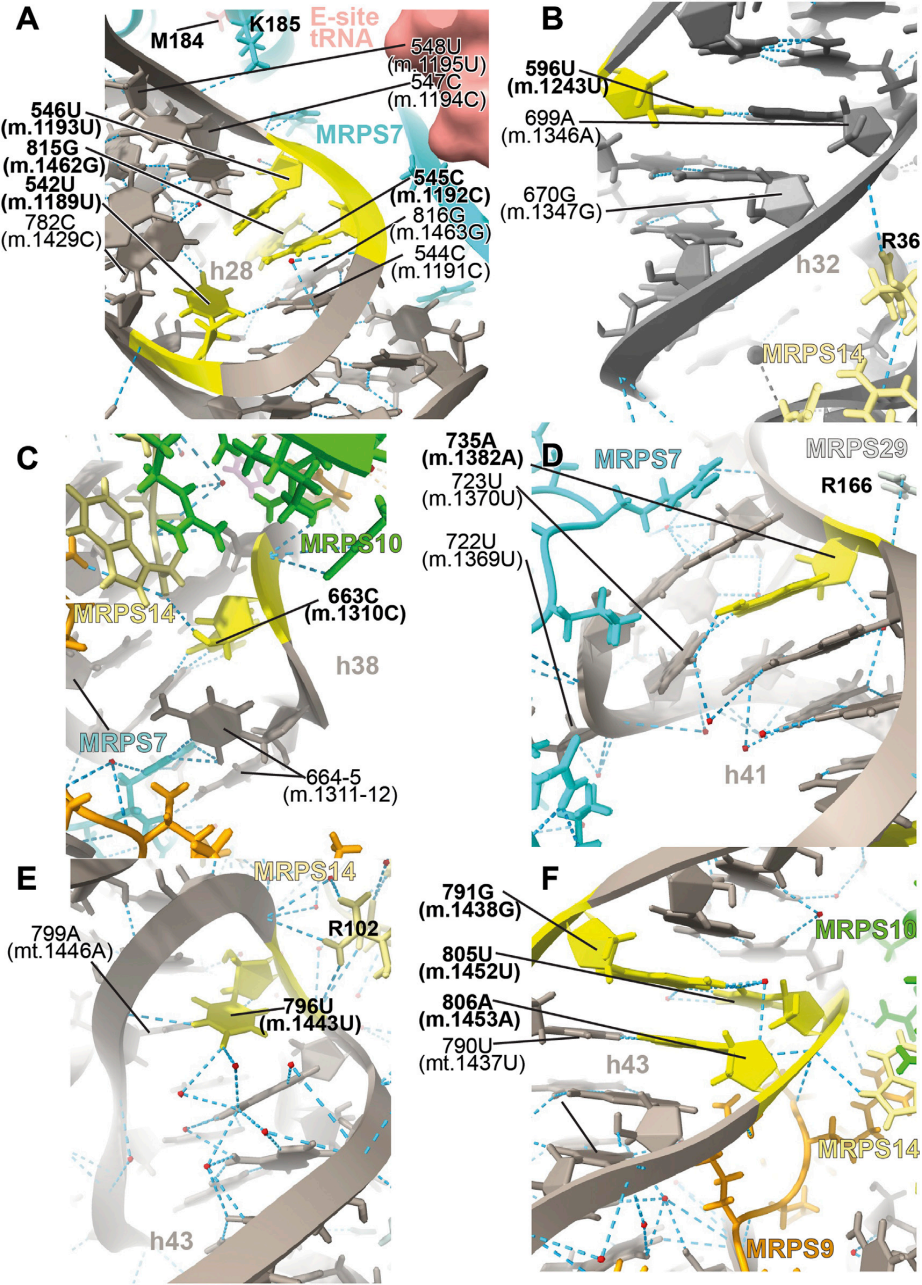
Structural inspection of the variants in the proximity of MRPS7/uS7m and MRPS14/uS14m. **(A–F)** Annotated view of the regions containing the following variants: 542U>C (m.1189U>C), 545C>A (m.1192C>A), 546U>C (m.1193U>C), and 815G>A (m.1462G>A) **(A)**; 596U>C (m.1243U>C) **(B)**; 663C>U (m.1310C>U) **(C)**; 735A (m.1382A) **(D)**; 796U>C (m.1443U>C) **(E)**; and 791G>A (m.1438G>A), 805U>C (m.1452U>C), and 806A>G (m.1453A>G) **(F)**. Color coding and labeling as in Figure 3.

Several deafness-associated variations mapping to the E site of the mt-SSU have been identified in hearing-impaired patients, namely, **542U>C (m.1189U>C)** (Konings et al., 2008; Lu et al., 2010; Rydzanicz et al., 2010; Meza et al., 2011; Guaran et al., 2013), **545C>A/U (m.1192C>A/U)** (Lu et al., 2010), **546U>C (m.1193U>C)** (Guaran et al., 2013), and **815G>A (m.1462G>A)** (Elstner et al., 2008; Konings et al., 2008; Padma and Ramchander., 2008; Lu et al., 2010). Positions **545C (m.1192C)** and **815G (m.1462G)** form a Watson:Crick base pair at the distal end of h28 (Figure 9A and Supplementary Figure S12A). This region packs tightly against the primary RNA-binding protein MRPS7/uS7m (Figure 9A), which is a conserved component of the head of the mt-SSU (Cavdar Koc et al., 2001). Contacts between MRPS7/uS7m and the RNA backbone are observed at positions 547C (m.1194C) and 548U (m.1195U) adjacent to the site of variation **546U (m.1193U)** (Figure 9A). Notably, one of these contacts is established with MRPS7/uS7m Lys 185 (Figure 9A), adjacent to Met 184, where a pathogenic mutation has been identified (Menezes et al., 2015). Additionally, the distance between the backbone at position **545C (m.1192C)** and the functionally important *β*-hairpin of protein MRPS7/uS7m is less than 3 Å (not shown) (Devaraj et al., 2009).

The **542U>C (m.1189U>C)** variant was identified in several independent studies (Konings et al., 2008; Lu et al., 2010; Rydzanicz et al., 2010; Meza et al., 2011; Guaran et al., 2013). In the secondary structure map of the SSU rRNA, position **542U (m.1189U)** maps to the single-stranded stretch of h28 (Supplementary Figure S12A). In the 2.2-Å cryo-EM structure position, **542U (m.1189U)** is nearly on the same plane as the **545C:815G (m.1192C:1462G)** base pair but slightly tilted, allowing it to use its O2 to form a hydrogen bond with the neighboring 544C:816G (m.1191C:1463G) base pair (Figure 9A). The tilting also allows a second hydrogen bond from **542U (m.1189U)** O4 to the NH_2_ of 782C (m.1429C) (Figure 9A), a contact that would be lost because of the **542U>C (m.1189 U>C)** variant. **542U>C (m.1189U>C)** was found together with the **141A>G (m.1811A>G)** variant in 16S mt-rRNA and the R127H allele in gene GJB2 (present in heterozygosity) (Guaran et al., 2013), both of which are likely polymorphisms (see below) (Thonnissen et al., 2002).

In addition to elongation, this region of the mito-ribosome is also implicated in translation initiation. Khawaja et al. (2020) showed that h28 rotates during initiation, resulting in the swiveling of the SSU head. Helix h28 becomes compressed in going from the so-called open to the closed state (not to be confused with the “open-closed” transition observed in the elongation phase during decoding) (Khawaja et al., 2020). Inspection of EM density from both the “open” and “closed” states shows that the distal end of h28, harboring the aforementioned sites of variation, is implicated in SSU swiveling (not shown) (Khawaja et al., 2020).

All this evidence strongly suggests that the **545C:815G (m.1192C:1462G)** base pair and the tertiary interactions of **542U (m.1189U)** are important to stabilize the structurally complex and dynamic junction between helices h28 and h29. Of the three variants affecting the **545C:815G (m.1192C:m.1462G)** base pair, **545C>A (m.1192C>A)** and **815G>A (m.1462G>A)** would replace a Watson:Crick base pair with either an A•G or C•A mismatch, while **545C>U (m.1192C>U)** would result in a disruptive U•G wobble at the end of a helical segment (Ananth et al., 2013). For all these reasons, these variants constitute potential candidates for pathogenic mutations possibly affecting the initiation and elongation phases of mitochondrial translation. In contrast to these residues, the base of **546U (m.1193U)** makes no hydrogen bonds which, together with the fact that a C is found in the *S. scrofa* structure at this position (position 544 in structure 5AJ4), strongly argues for a silent phenotype associated with the **546U>C (m.1193U>C)** variant.

##### −596U>C (m.1243U>C)

The haplotype marker **596U>C (m.1243U>C)** was found in association with deafness in several studies (Ballana et al., 2006; Konings et al., 2008; Rydzanicz et al., 2010; Guaran et al., 2013). Position **596U (m.1243U)** maps to helix h32 of 12S mt-rRNA, where it base pairs to 699A (m.1346A) (Figure 9B and Supplementary Figure S13A). Arg 36 of MRPS14/uS14m (beige in Figure 9B) forms a hydrogen bond to the backbone at position 700G (m.1347G). Hence, the disruption of the **596U:**699A **(m.1243U:**1346A**)** base pair by the U>C base change at position **596U (m.1243T)** could affect the interaction of h32 and MRPS14/uS14m in this region. Although the abundance of the **596U>C (m.1243U>C)** variation implies that the base change is well-tolerated, whether it could lead to slight phenotypic effects remains uncertain.

##### −663C>U (m.1310C>U)

The **663C>U (m.1310C>U)** variant has been identified in a Japanese patient with inherited sensorineural hearing loss (Yano et al., 2014). Position **663C (m.1310C)** maps to h38 of 12S mt-rRNA, where it base pairs with 668G (m.1315G) (Figure 9C and Supplementary Figure S14A). Its O2′ makes a hydrogen bond with the terminal carboxyl group of protein MRPS14/uS14m (beige in Figure 9C). Late-binding protein MRPS10/uS10m (lime in Figure 9C) also contacts the backbone of 663C (m.1310C). Additional protein:RNA contacts are observed next to 663C (m.1310C) involving MRPS7/uS7m (cyan in Figure 9C) and the adjacent positions 664–5 (m.1311–12). The C>U base change at position **663C (m.1310C)** would create a disfavored U•G wobble base pair at the end of a helical segment (Ananth et al., 2013) with the potential to distort the local structure.

##### −735A>C (m.1382A>C)

The **735A>C (m.1382A>C)** variant was identified in two families with hearing loss (Mkaouar-Rebai et al., 2010). Position **735A (m.1382A)** is base-paired to 723U (m.1370U) at the proximal end of h41 in the head of the mt-SSU (Figure 9D and Supplementary Figure S15A). A contact from Arg 166 of MRPS29/mS29 (light gray in Figure 9D) to the RNA backbone at 735A (m.1382A) is visible. Protein MRPS7/uS7m (cyan in Figure 9D) establishes several hydrogen bonds with the surrounding RNA residues. Notably, the adjacent 722U (m.1369U) is less than 10 Å away from Met 184 of MRPS7/uS7m, where a pathogenic mutation has been identified (not shown) (Menezes et al., 2015). As the A>C base change would result in the loss of the 723U:**735A (**m.1370U:**m.1382A)** base pair, the pathogenic potential of this variant must be considered.

##### −796U>C (m.1443U>C)

The highly rare **796U>C (m.1443U>C)** variant was identified in a non-syndromic hearing-impaired Chinese pediatric subject (Li et al., 2005). Position **796U (m.1443U)** maps to the loop that caps h43 of 12S mt-rRNA (Figure 9E and Supplementary Figure S16A). The N3 of **796U (m.1443U)** establishes a hydrogen bond with the RNA backbone at position 799 (m.1446) (Figure 9E), an interaction that would be precluded by the U>C base change. Contacts from Arg 102 of MRPS14/uS14m (beige in Figure 9E) and the RNA backbone at positions 796-**7 (**m.**1443-4)** are visible in the structure. The adjacent position 797A (m.1444A) makes a tertiary interaction by forming a triple base interaction with 585A (m.1232A) of h31 and 753G (m.1401G) of h42 (Supplementary Figure S16C). This and additional tertiary interactions established by adjacent residues dock this capping loop of h43 into the minor groove of h42 and, at the same time, provide structure to the single-stranded stretch located at the distal end of the functionally important helix h31. The surface created by this interaction is recognized by the protein MRPS14/uS14m, which places Arg 108, a position where pathogenic mutations have been identified, only ∼12-Å away from **796U (m.1443U)** (not shown) (Jackson et al., 2019). Given all this evidence, the disruptive potential **796U>C (m.1443U>C)** must be considered significant.

##### −791G>A (m.1438G>A), 805U>C (m.1452U>C), and 806A>G (m.1453A>G)

The sites of variation **805U (m.1452U)** and **806A (m.1453A)**, presumably associated with hearing impairment, map to h43 of 12S mt-rRNA (Figure 9F and Supplementary Figure S17A) (Padma and Ramchander, 2008; Lu et al., 2010; Rydzanicz et al., 2010). Proteins MRPS9/bS9 and MRPS10/uS10m (orange and lime, respectively, in Figure 9F) contact the RNA backbone at position **805U (m.1452U),** while MRPS9/bS9 and MRPS14/uS14m (beige in Figure 9F) do the same at position **806A (m.1453A)**. Position **805U (m.1452U)** forms a wobble base pair with **791G (m.1438G**) (Figure 9F and Supplementary Figure S17A), another variant site that has been repeatedly associated with hearing impairment (Li et al., 2005; Konings et al., 2008; Rydzanicz et al., 2010; Guaran et al., 2013). In fact, the Cambridge reference sequence (RefSeq ID: NC_012920.1) carries an A at **791 (m.1438)**, which is present in only 5% of the GenBank sequences (Andrews et al., 1999; Ruiz-Pesini et al., 2007). These data indicate that both the Watson:Crick and wobble geometry are tolerated at the **791/805 (m.1438/1452)** base pair. As a result, both the **805U>C (m.1452U>C)** and the **791G>A (m.1438G>A)** variants should be considered silent, as they would result in the exchange of the wobble configuration seen in the structure for a Watson:Crick base pair. Preceding the **791/805 (m.1438/1452)** base pair lies position **806A (m.1453A),** which forms a Watson:Crick base pair with 790U (m.1437U) (Figure 9F and Supplementary Figure S17A). A deafness-associated A>G base change has been detected at **806A (m.1453A)** (Rydzanicz et al., 2010) that would replace the Watson:Crick base pair with a U•G wobble. The effects of this variant could be haplotype sensitive, as argued in the discussion section.

#### Variants in the proximity of the early binding proteins MRPS2/uS2m, MRPS23/uS23m, and MRPS17/uS17m

**FIGURE 10:**
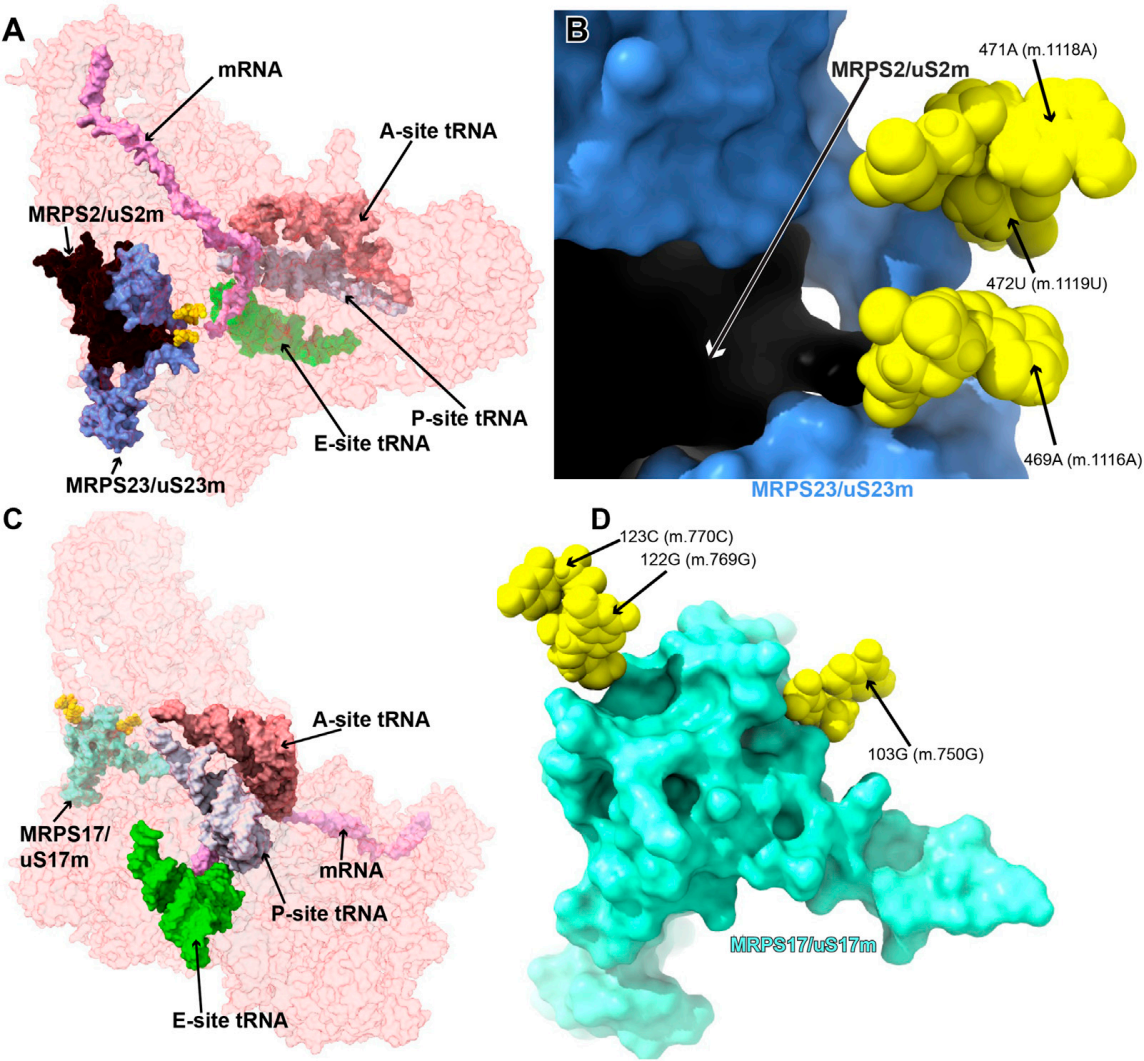
Variants in the proximity of MRPS2/uS2m, MRPS23/uS23m, and MRPS17/uS17m. A. Sites of potential deafness-associated variations (yellow) in the neighborhood of MRPS2/uS2m (black) and MRPS23/uS23m (light blue), shown in the context of the mt-SSU (semi-transparent, red surface). **(B)** Blown-up version of A showing the three sites of variation associated with this region of the mt-SSU (yellow), with their positions indicated. Mito-ribosomal proteins color-coded as in **(A)**. **(C)** Sites of potential deafness-associated variations (yellow) in the neighborhood of MRPS17/uS17m (aquamarine), shown in the context of the mt-SSU (semi-transparent, red surface). Mito-ribosomal ligands color coded as in Figure 2B. **(D)** Blown-up version of **(C)** showing the three sites of variation associated with this region of the mt-SSU (yellow), with their positions indicated. Mito-ribosomal proteins color-coded as in **(C)**.

The early binding proteins MRPS2/uS2m and MRPS23/uS23, together with MRPS28/bS1m, bind as a cluster to the solvent side of the 12S mt-rRNA molecule (Bogenhagen et al., 2018). Pathogenic mutations have been identified in MRPS2/uS2m and MRPS23/uS23m (Kohda et al., 2016; Gardeitchik et al., 2018). While no pathogenic mutations mapping to MRPS17/uS17m have been so far identified (Sanchez et al., 2021), its role as a primary binding protein makes it a central component of the mito-ribosome. For this reason, 12S mt-rRNA variants that affect the binding of MRPS17/uS17m must be considered potentially disruptive. In this section, we assessed the potential pathogenicity of six variants mapping to the vicinity of these proteins (Figure 10).

##### –469A>G (m.1116A>G), 471A>G (m.1118A>G), and 472U>C (m.1119U>C)

**FIGURE 11:**
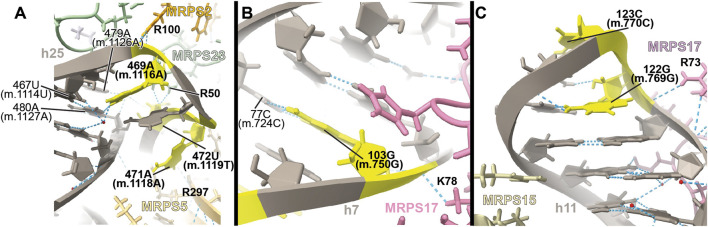
Structural inspection of the variants in the proximity of MRPS2/uS2m, MRPS23/uS23m, and MRPS17/uS17m. **(A–C)** Annotated view of the regions containing the following variants: 469A>G (m.1116A>G), 471A>G (m.1118A>G), and 472U>C (m.1119U>C). **(B)** 103G>A (m.750G>A). **(C)** 122G>A (m.769G>A) and 123C>U (m.770C>U). Color coding and labeling as in Figure 3.

The **469A>G (m.1116A>G)**, **471A>G (m.1118A>G)**, and **472U>C (m.1119U>C)** variants were found in four hearing-impaired patients (Li et al., 2005; Guaran et al., 2013). Positions **471A (m.1118A)** and **472U (m.1119U)** are located in the loop capping h25 (Supplementary Figure S18A) toward the back of the subunit. The base of **471A (m.1118A)** has been modeled within 3 Å of Arg 297 of protein MRPS5/uS5m (beige in Figure 11A) and ∼13-Å away from the *ram* residue V336 within the same protein (not shown) (Akbergenov et al., 2018). Despite this, the low quality of the density attributed to 471A (m.1118A) (Figure 11A and Supplementary Figures S18C, D) suggests a disordered conformation for this residue. Hence, its disruptive role cannot be possibly established with the structural data alone. As for **472U (m.1119U)**, its base has been modeled, making a water-mediated hydrogen bond, through its O2, to the base and the sugar of 479A (m.1126A) and 480A (m.1127A), respectively (Figure 11A). In addition, its ribose O4′ establishes two direct hydrogen bonds to Arg 50 of MRPS23/uS23 (light green in Figure 11A). All the observed interactions involving **472U (m.1119U)** would be unaffected by a U>C base change, thus making **472U>C (m.1119U>C)** a good candidate for a silent variant. In this particular patient, the presence of **62G>A (m.709G>A)** together with **472U (m.1119U)** could offer a better explanation for the observed deafness, as explained previously (Guaran et al., 2013). The situation of the **469A>G (m.1116A>G)** variant is different in that its base is part of a base triple with the 467U:480A (m.1114U:1127A) that would be likely affected by the base change. Additionally, Arg 100 of MRPS2/uS2m (orange in the structure) is within hydrogen bonding distance of the RNA backbone at position **469A (m.1116A)**. The density around **469A (m.1116A)** supports these interactions. Hence, despite the peripheral location of the **469A (m.1116A)** variant, the structural evidence does not permit its scoring as a silent mutation.

##### −103G>A (m.750G>A)

Position **103 (m.750)** is an A in the Cambridge mtDNA reference sequence (Andrews et al., 1999). However, the A>G polymorphism is highly abundant, present in 53,668 out of 54,594 GenBank sequences, according to MITOMAP (Ruiz-Pesini et al., 2007). Base changes in this position have been frequently implicated in hearing loss (Li et al., 2005; Konings et al., 2008; Lu et al., 2010; Rydzanicz et al., 2010; Guaran et al., 2013). In the 2.2-Å structure of the mito-ribosome, position **103 (m.750)** is a G that forms a Watson:Crick base pair to 77C (m.724C) in h7 (Figure 11B and Supplementary Figure S19A). A contact from MRPS17/uS17m Lys 78 (pink in the structure) to the backbone at positions **103G (m.750G)** is visible (Figure 11B). Although the G>A base change is clearly tolerated at position **103G (m.750G)**, the introduction of an A•C mismatch at this position in h7 is expected to cause some level of structural distortion whose overall consequences cannot be predicted. Hence, the **103G>A (m.750G>A)** base change is another example of a haplotype marker with a potentially pathogenic role.

##### −122G>A (m.769G>A) and 123C>U (m.770C>U)

The haplotype marker **122G>A (m.769G>A)** and the **123C>U (m.770C>U)** variant were described in the context of deafness in two studies (Elstner et al., 2008; Konings et al., 2008). Positions **122G (m.769G)** and **123C (m.770C)** map to the GNRA tetraloop capping h11 (Figure 11C and Supplementary Figure S20A) (Correll and Swinger., 2003). This tetraloop is part of the binding site of protein MRPS17/uS17m (pink in Figure 11C), which contacts the 12S mt-rRNA backbone at position 122G (m.769G). Protein MRPS15/uS15 (beige in Figure 11C) is also in the neighborhood. Although the **123C>U (m.770C>U)** variant would not change the GNRA consensus, **122G>A (m.769G>A)** would do so, likely disrupting the structure of the tetraloop and, perhaps, the binding of MRPS17/uS17m. Given the role of MRPS17/uS17m as an early binding protein, the possibility of disruptive potential in the case of the **122G>A (m.769G>A)** variant must be considered.

#### Variants mapping to mito-ribosomal inter-subunit bridges

**FIGURE 12:**
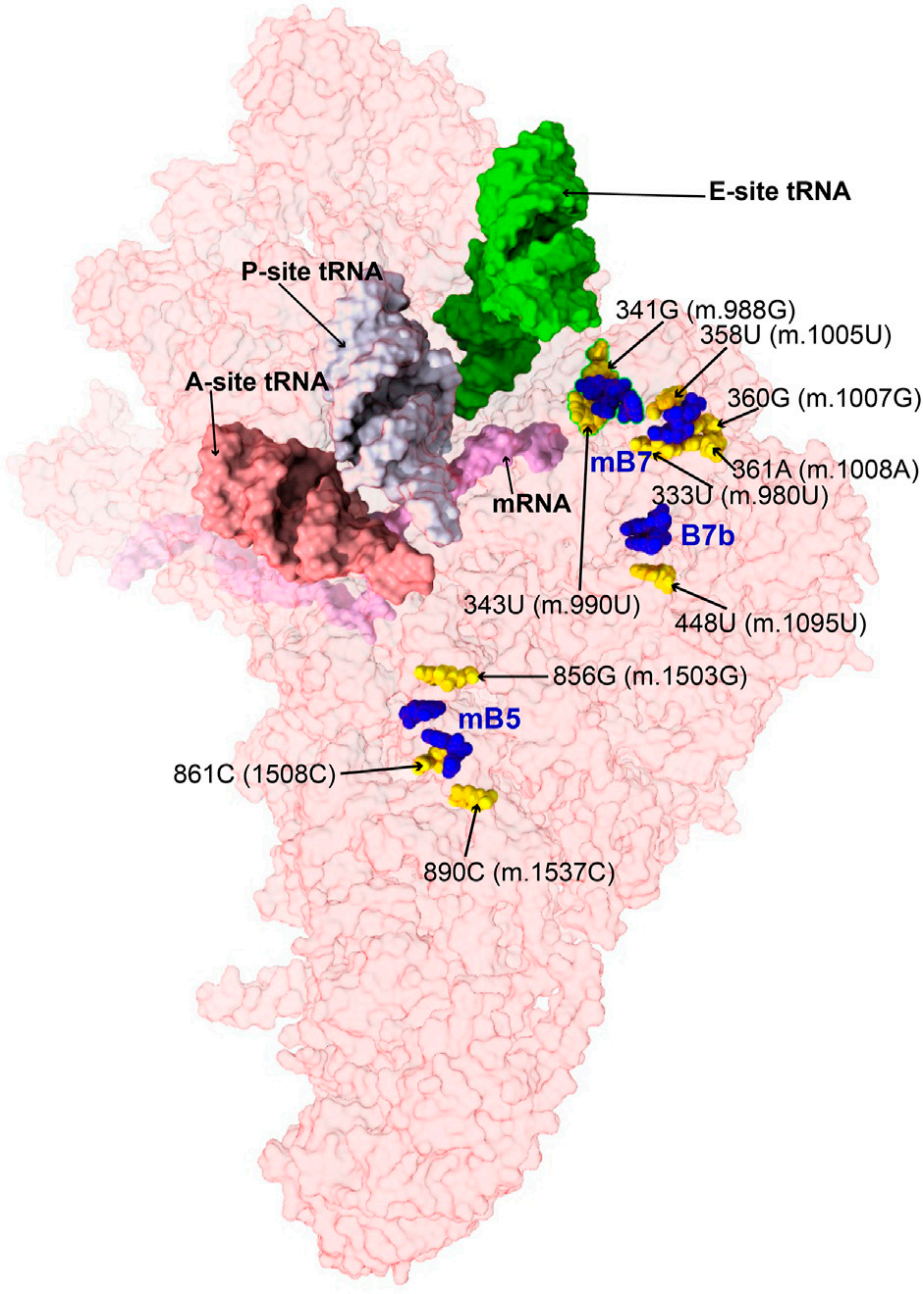
Variant mapping to mito-ribosomal inter-subunit bridges. Sites of potential deafness-associated variations (yellow) in the neighborhood of mito-ribosomal inter-subunit bridges (blue), shown in the context of the mt-SSU (semi-transparent, red surface). Mito-ribosomal ligands color coded as in Figure 2B.

In contrast to bacterial and cytoplasmic ribosomes, where bridges are mostly formed by RNA-RNA interactions, protein-mediated bridges are abundant in mammalian mito-ribosomes (Amunts et al., 2015; Greber et al., 2015). Studies on bacteria have shown that mutation of bridge residues can affect growth to a variable degree, often resulting in fidelity phenotypes (Sun et al., 2011; Hoffer et al., 2019), prompting the study of eight variants mapping to the vicinity of mito-ribosomal bridges (Figure 12).

##### −333U>C (m.980U>C), 358U>C (m.1005U>C), 360G>A (m.1007G>A), and 361A>G (m.1008A>G)

**FIGURE 13:**
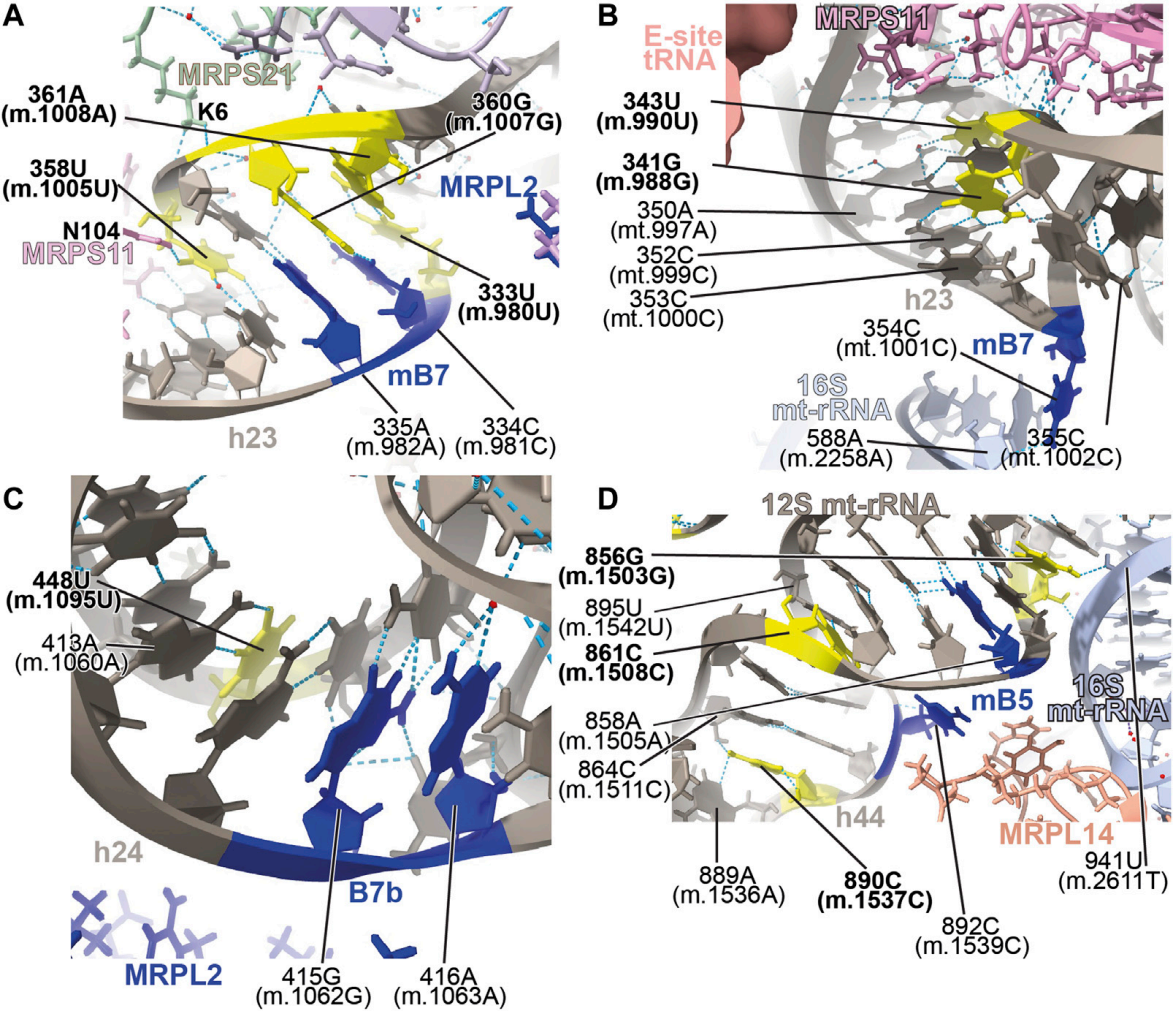
Structural inspection of the variants in the proximity of mito-ribosomal bridges. **(A–D)** Annotated view of the regions containing the following variants: **333U>C (m.980U>C)**, **358U>C (m.1005U>C)**, **360G>A (m.1007G>A)**, and **361A>G (m.1008A>G)** **(A)**; **341G>A (m.988G>A)** and **343U>C (m.990U>C)** **(B)**; **448U>C (m.1095U>C)** **(C)**; **856G>A (m.1503G>A)**, **861C>U (m.1508C>U)** and **890C>U (m.1537C>U)** **(D)**. E-site tRNA shown as salmon surface in **(B)**. Bridge residues are shown in blue. Additional color coding and labeling as in Figure 3.

Positions **333U (m.980U)**, **358U (m.1005U)**, **360G (m.1007G)**, and **361A (m.1008A)** map to h23 of 12S mt-rRNA (Supplementary Figure S21A) (Li et al., 2005; Guaran et al., 2013; Yano et al., 2014). The base of **358U (m.1005U)** is involved in a water-mediated, non-canonical base pair with 336C (m.983C) and a quaternary contact with Asn 104 of MRPS11/uS11m (pink in Figure 13A). Positions **360G (m.1007G)** and **361A (m.1008A)** form Watson:Crick base pairs with residues 334C (m.981C) and **333U (m.980U)**, respectively. Lys 6 of MRPS21/bS21 contacts the RNA backbone at positions 359U (m.1006U) and **360G (m.1007G)** (light green in Figure 13A). This region of h23 harbors the mitochondrial-specific inter-subunit bridge mB7, formed in the ratcheted state of the mito-ribosome between positions 334-5 (m.981-982) of 12S mt-rRNA and mt-LSU protein MRPL2/uL2m (blue RNA and protein residues in Figure 13A) (Amunts et al., 2015). Given the functional importance of the region, variants **333U>C (m.980U>C)** and **360G>A (m.1007G>A)** would both result in a disruptive C•A mismatch, while the **361A>G (m.1008A>G)** would give rise to a less damaging U•G wobble. In the case of **358U>C (m.1005U>C)**, the hydrogen bonding pattern observed in the structure would not be maintained, possibly causing some degree of structural disruption. All variants have been identified in MITOMAP with moderate-to-high frequency (two are haplotype markers) (Supplementary Table S1) (Ruiz-Pesini et al., 2007), indicating that they all can be tolerated. Despite this, due to the involvement of this region of h23 in the functioning of bridge mB7, even slight structural distortions could generate measurable phenotypic effects.

##### −341G>A (m.988G>A) and 343U>C (m.990U>C)

Variants at positions **341G (m.988G)** and **343U (m.990U)** of h23 were found in the studies of Konings et al. (2008) and Rydzanicz et al. (2010). These residues are base-paired to 352C (m.999C) and 350A (m.997A), respectively (Figure 13B and Supplementary Figure S22A). However, although the 341G:352C (m.988G:999C) base pair adopts a Watson:Crick configuration, positions 343U (m.990T) and 350A (m.997A) form a non-canonical base pair with two hydrogen bonds (one of them water-mediated). Contacts from MRPS11/uS11m to the RNA backbone at positions 989–991 and to the base of 344G (m.991G) are visible (Figure 13B). During elongation, this region of h23 is in close proximity to E-site tRNA (salmon density in Figure 13B). A sharp turn of the RNA backbone between positions 353 (m.1000) and 355 (m.1002) extrudes 354C (m.1001C) out of the rRNA helix (blue in Figure 13B), pointing its base toward the LSU, where it makes contact with the ribose of 588A (m.2258A) of H68 in 16S mt-rRNA, thus defining bridge B7a (Figure 13B) (Amunts et al., 2015). During translation initiation, both MRPS11/uS11m and the major groove of h23 are contacted by mitochondrial initiation factor 3 (MTIF3), underscoring the structural and functional importance of this region of the SSU in both bridge formation and translation initiation (Supplementary Figure S22E). As both variants, **341G>A (m.988G>A)** and **343U>C (m.990U>C)** would disrupt the base hydrogen bonding pattern observed in the two high-resolution structures used in this analysis (Khawaja et al., 2020; Itoh et al., 2021; Itoh et al., 2022), they are considered good candidates for pathogenic mutations potentially affecting the initiation and/or the elongation phases of mitochondrial translation.

##### −448U>C (m.1095U>C)

The **448U>C (m.1095U>C)** variant was found in several patients with sensorineural and non-syndromic hearing loss (Tessa et al., 2001; Zhao L. et al., 2004; Muyderman et al., 2012; Yano et al., 2014). Position **448U (m.1095U)** base-pairs to 413A (m.1060A) in h24 of 12S mt-rRNA (Figure 13C and Supplementary Figure S23A). This base pair is very close to the highly dynamic inter-subunit bridge B7b, formed between residues 415–6 (m.1062–1063) of h24 and LSU protein MRPL2/uL2m (blue 12S mt-rRNA and protein residues in Figure 13C) (Amunts et al., 2015). Our group mutagenized the heterologous equivalent bridge in bacteria, resulting in impaired growth and decreased fidelity during initiation (Sun et al., 2011). Hence, the disruption of the 413A:**448U (m.**1060A:**1095U)** base pair by the U>C transition has the potential to affect the functioning of bridge B7b and cause translational defects. Results of biochemical analyses with cybrids carrying this variant in heteroplasmy were consistent with decreased mitochondrial function and increased AG susceptibility (Muyderman et al., 2012). Hence, our structural interpretation provides a rationale for previous reports claiming a pathogenic role for this variant (Tessa et al., 2001; Muyderman et al., 2012).

##### −856G>A (m.1503G>A), 861C>U (m.1508C>U), and 890C>U (m.1537C>U)

The **856G>A (m.1503G>A)** variant was identified by Rydzanicz et al. in a patient with non-syndromic and aminoglycoside-induced hearing loss and by Chen et al. in a Uyghur patient with non-syndromic deafness (Rydzanicz et al., 2010; Chen et al., 2011). Position **856G (m.1503G)** is near the highly conserved bridge mB5, which according to lower-resolution structures of the human mito-ribosome, involves positions 858A (m.1505A) and 892C (m.1539C) in h44 of 12S mt-rRNA and MRPL14/uL14m in the LSU (Figure 13D and Supplementary Figure S22A) (Amunts et al., 2015). The 2.2-Å structure clearly shows the amino group of 856G (m.1503G) within the hydrogen bonding distance of the O2′ of 941U (m.2611T) of 16S mt-rRNA (Figure 13D), thus prompting the inclusion of both positions among the group of mB5 residues.

Variants **861C>U (m.1508C>U)** and **890C>U (m.1537C>U)** also map to h44 of 12S mt-rRNA and the neighborhood of mB5 (Figure 13D and Supplementary Figure S23A). Position **861C>U (m.1508C>U)** has been identified in a patient with profound hearing loss without previous exposure to AGs (Padma et al., 2012). This residue forms a single-hydrogen-bond base pair with 895U (m.1542U) (Figure 13D). As for the **890C>U (m.1537C>U)** variant, it has been identified in two patients with non-syndromic hearing impairment (Estivill et al., 1998a; Leveque et al., 2007; Konings et al., 2008). The amino group of **890C (m.1537C)** establishes two hydrogen bonds, one with the N3 of the adjacent 889A (m.1536A) and the other with the O2′ of 864C (m.1511C) (Figure 13D).

Our group has performed mutagenesis of this bridge in bacteria, showing that mutations in mB5 affect both growth and fidelity (Sun et al., 2011; Liiv and O'Connor., 2006). With this in mind, it seems reasonable to think that the loss of one or two hydrogen bonds around the bridge residue 892C (m.1539C) due to the **861C>U (m.1508C>U)** or **890C>U (m.1537C>U)** variants could affect the proper functioning of mB5. Regarding **856G>A (m.1503G>A)**, its high frequency in the population clearly indicates that an A must be tolerated at this position. However, the loss of a quaternary interaction involving **856G (m.1503G)** and 941U (m.2611T) of 16S mt-rRNA observed in the 2.2-Å cryo-EM human mito-ribosomal structure (Itoh et al., 2021; Itoh et al., 2022) is expected to affect the function of bridge mB5, thus making this variant a good candidate for a pathogenic base change.

#### Variants near the mRNA channel and A-site tRNA

**FIGURE 14:**
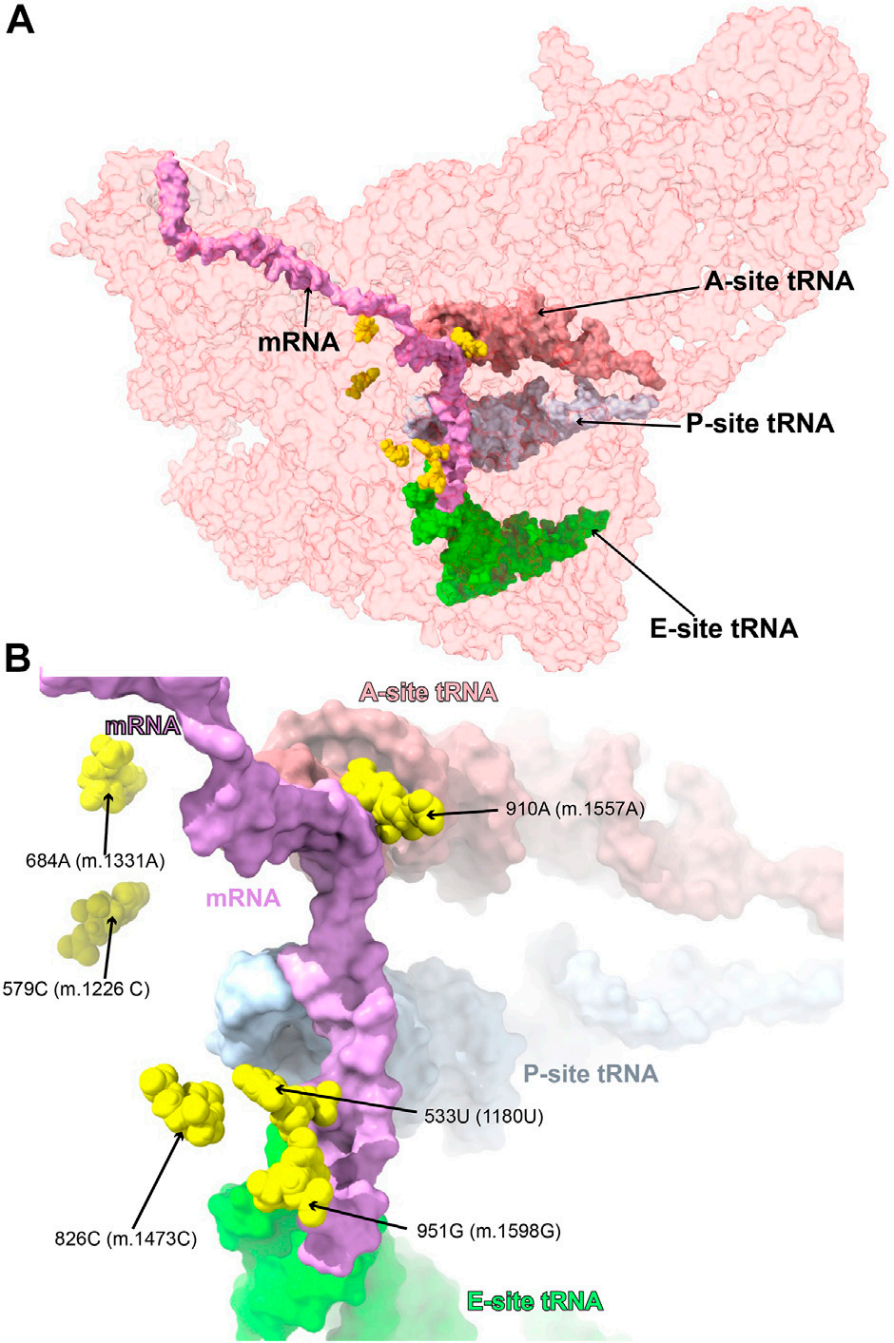
Variants near the mRNA channel and A-site tRNA. Sites of potential deafness-associated variations (yellow) in the neighborhood of the mRNA channel and A-site tRNA, shown in the context of the mt-SSU (semi-transparent, red surface). Mito-ribosomal ligands color coded as in Figure 2B. **(B)** Blown-up version of **(A)** showing the six sites of variation associated with this region of the mt-SSU (yellow), with their positions indicated.

Six variants mapped to 12S mt-rRNA positions in the neighborhood of the mRNA channel and the A-site tRNA binding sites (Figure 14), possibly interfering with the binding of these mito-ribosomal ligands and the process of mt-translation.

##### −533U>G (1180U>G) and 826C>U (m.1473C>U)

**FIGURE 15:**
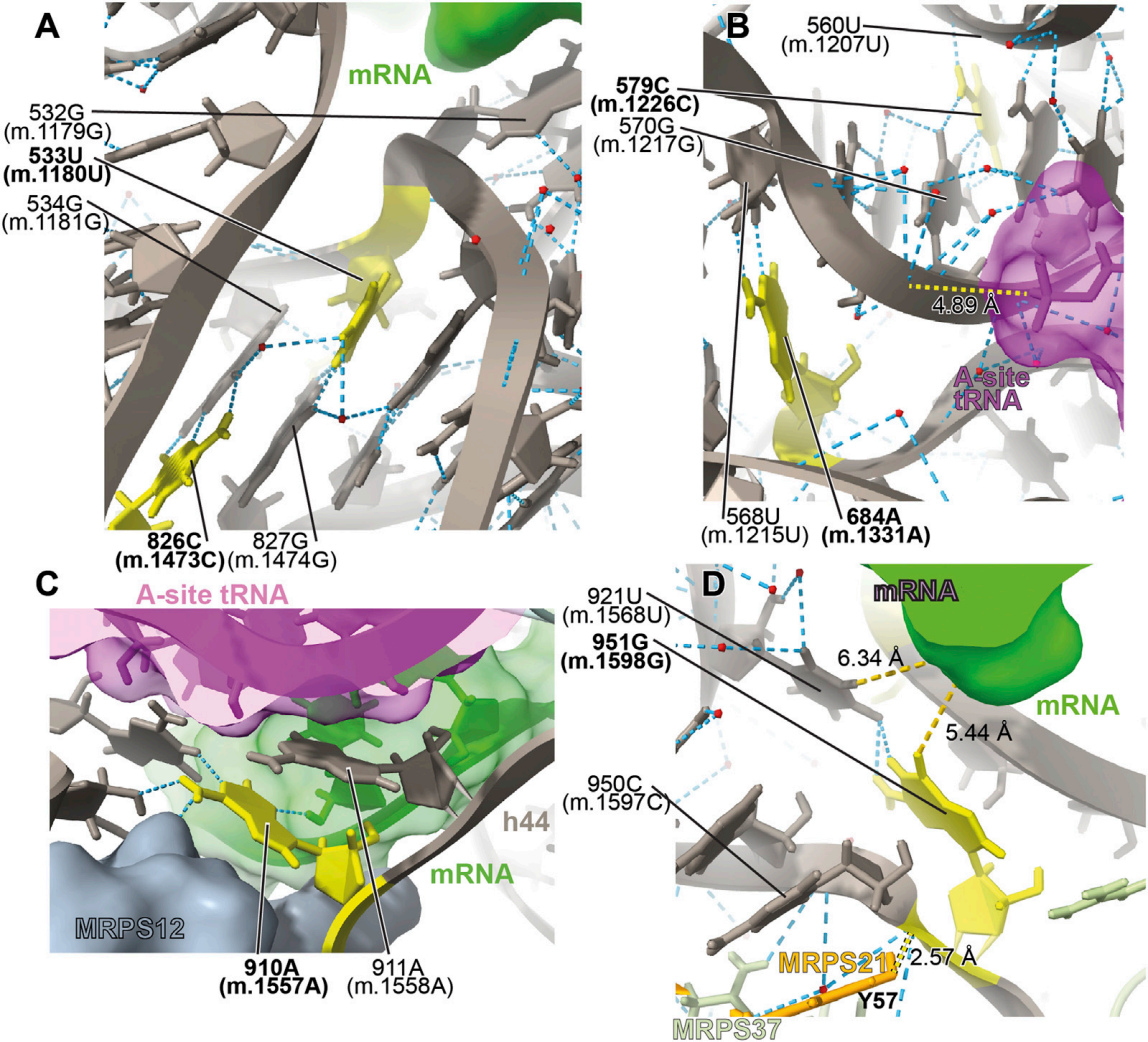
Structural inspection of the variants in the proximity of the mRNA channel and the A-site tRNA. **(A–D)** Annotated view of the regions containing the following variants: **533U>G (m.1180U>G)** and **826C>U (m.1473C>U)** **(A)**; **579C>G (m.1226C>G)** and **684A>G (m.1331A>G)** **(B)**; **910A>C (m.1557A>C)** **(C)**; and **951G>A (m.1598G>A)** **(D)**. MRNA shown as a green surface in **(A, C, D)**. A-site tRNA shown as a magenta surface in **(B)** and **(C)**. Additional color coding and labeling as in Figure 3.

The very rare **533U>G (m.1180U>G)** variant was found in homoplasmy in pediatric subjects with non-syndromic hearing loss (Li et al., 2004). We have analyzed this base change as part of our earlier implementation of the HIA method (Smith et al., 2014) and described it as an expectedly disruptive mutation. The availability of high-resolution structures of the human mito-ribosome prompted to revisit our initial assignment. In the mito-ribosome, positions **533U>G (m.1180U>G)** and 827G (m.1474G) of h28 form a wobble base pair (Figure 15A and Supplementary Figure S25A). Next to the **533U**•827G **(m.1180U**•m.1474G**)** wobble lies position **826C (m.1473C)**, where a **C>U** transition was found in a patient with severe hearing loss (Chen et al., 2018). Position **826C (m.1473C)** forms a Watson:Crick base pair with 534G (m.1181G) (Figure 15A and Supplementary Figure S25A). On the 3’ side of **533U (m.1180U)** lies the universally conserved 532G (m.1179G) (*E. coli* G926, conservation between 98% and 100%) (Cannone et al., 2002), which is hydrogen bonded to the phosphate linking the P- and E-site codons on the mRNA (Carter et al., 2000) (Figure 15A). Strikingly, in *E. coli*, all three base changes are tolerated at position 926 of 16S rRNA, with moderate-to-severe effects on cell growth (Vila-Sanjurjo et al., 1999), but with an important reduction of ribosomal activity *in vitro* (Abdi and Fredrick., 2005). Mutagenesis studies performed in yeast have shown that several base changes introduced at the equivalents of positions 532G (m.1179G), **533U>G (m.1180U>G)**, and 534G (m.1181G) (bacterial 926, 927, and 928) in the cytoplasmic ribosome confer lethal phenotypes (Dong et al., 2008). In the case of the heterologous equivalents of **533U (m.1180U)** and 534G (m.1181G) (bacterial 927 and 928, respectively), compensatory mutations restoring base pairing potential also restored growth (Dong et al., 2008). As the **533U>G (m.1180U>G)** variant would replace a U•G wobble with a G•G mismatch, its disruptive potential must be considered high. Hence, the use of the new structural data corroborates our previous assignment as an expectedly disruptive mutation. In the case of **826C>U (m.1473C>U)**, the transition would replace a Watson:Crick G:C with a G•U wobble, creating a highly non-isosteric U•G/G•U tandem wobble combination (Ananth et al., 2013). Although the **826C>U (m.1473C>U)** variant is well tolerated, as judged by its high frequency (Supplementary Table S1), subtle defects, such as altered fidelity, cannot be dismissed.

##### −579C>G (m.1226C>G) and 684A>G (m.1331A>G)

The **579C>G (m.1226C>G)** variant was found in a pediatric subject with non-syndromic hearing loss (Li et al., 2004). Position **579C (m.1226C)** is universally conserved (Cannone et al., 2002). In the secondary structure of 12S mt-rRNA, position **579C (m.1226C)** is located at the single-stranded junction connecting helices h31 and h32 (Figure 15B and Supplementary Figure S26A). In the three dimensional structure of the mito-ribosome, position **579C (m.1226C)** canonically base-pairs with 570G (m.1217G), also universally conserved (Cannone et al., 2002), and places its amino group within hydrogen bonding distance of the phosphate oxygen of 560U (m.1207U) of h30 (Figure 15B and Supplementary Figure S26A). On the other strand, 570G (m.1217G) is within 5Å of the backbone of A-site tRNA (magenta in Figure 15B). Mutagenesis studies in *E. coli* have targeted h31. When the heterologous equivalents of **579C (m.1226C)** and adjacent residues were mutated, they resulted in moderate-to-strong growth defects and elicited fidelity phenotypes (Yassin et al., 2005; Kubarenko et al., 2006). In agreement with our previous assignment for this **579C (m.1226C)** (Smith et al., 2014), we consider the C>G variant at this position as a good candidate for a pathogenic variant.

A second variant maps to the neighborhood of position **579C (m.1226C).** The **684A>G (m.1331A>G)** base change was identified in one Han Chinese pediatric subject with aminoglycoside-induced and non-syndromic hearing loss (Lu et al., 2010). Although **684A (m.1331A)** maps to h34, it is brought into the neighborhood of **579C (m.1226C)** by a tertiary interaction in the form of a trans-Watson:Crick base pair, with the base of 568U (m.1215U) (Figure 15B and Supplementary Figure S26A) (Leontis et al., 2002). This interaction would be disrupted by the A>G base change. Given the aforementioned importance of this region, in the neighborhood of A-site tRNA, **684A>G (m.1331A>G)** must be considered a potentially pathogenic variant.

##### −910A>C (m.1557A>C)

The **910A>C (m.1557A>C)** variation was identified in a Russian individual suffering from deafness (Tazetdinov and Dzhemileva, 2007). Position **910A (m.1557A)** maps to a single-stranded stretch of h44 (Supplementary Figure S27A). Its bacterial equivalent, the universally conserved A1492, is, together with A1493, one of the most important residues of the ribosome. The functional role of these two adenosines is to monitor the geometry of the codon-anticodon interaction at the A-site during decoding (Carter et al., 2000; Ogle et al., 2001; Ogle et al., 2002; Demeshkina et al., 2012). Given their crucial role in decoding, it has been shown in bacteria that mutations at A1492 or A1493 result in dominant lethal phenotypes (Yoshizawa et al., 1999; Abdi and Fredrick., 2005; Cochella et al., 2007). We discussed the disruptive role of the **910A>C (m.1557A>C)** variant in our previous analysis of highly rare variants mapping to 12S mt-rRNA, performed in the absence of high-resolution mito-ribosomal structures (Smith et al., 2014). The implication of **910A (m.1557A)** and 911A (m.1558A) in monitoring the geometry of A-site decoding is evident from the 2.2-Å structure of the mito-ribosome (Itoh et al., 2021; Itoh et al., 2022, see also Vila-Sanjurjo et al., 2023), which is in perfect agreement with the overall mechanism proposed in bacteria (Figure 15C) (Carter et al., 2000;Ogle et al., 2002; Ogle et al., 2001; Demeshkina et al., 2012). Hence, our previous prediction regarding the potential disruptive potential of this variant remains expectedly disruptive.

##### −951G>A (m.1598G>A)

The haplotype variant **951G>A (m.1598G>A)** was found in two aminoglycoside-induced hearing-impaired subjects (Li et al., 2005). In the secondary structure map of 12S mt-rRNA, **951G (m.1598G)** maps to the single-stranded stretch near the 3′end of the molecule (Supplementary Figure S28A). In the 2.2-Å structure of the 55S mito-ribosome, 951G (m.1598G) contacts the base of 921U (m.1568U) (Figure 15D and Supplementary Figure S28A). Contacts from protein MRPS37/mS37 to the RNA backbone at positions 950C (m.1597C) and 951G (m.1598G) are visible in the structure (lime green in Figure 15D). Protein MRPS37/mS37 has been implicated in restricting the rotation of the SSU during initiation (Khawaja et al., 2020). Additionally, protein MRPS21/bS21 (Tyr 57) is within ∼3 Å from the RNA backbone at position 951G (m.1598G) (Figure 15D). Finally, it should also be mentioned that the base of 951G (m.1598G) is within 5–6 Å of the mRNA upstream of the E site (green in Figure 15D). These data suggest that the G>A transition at **951G (m.1598G)** would impact its interaction with 921U (m.1568U), possibly resulting in a distortion of the mRNA channel and leading to phenotypic effects. These effects, however, must not be drastic, as the **951G>A (m.1598G>A)** base change has been found frequently in the population (Supplementary Table S1).

## Discussion

Here, we have undertaken the task of contextualizing a large collection of mt-rRNA variants thought to be associated with deafness. Out of the 92 mt-rRNA variants studied, 49 constituted valid candidates for deafness-inducing variants under the new framework. Although the lack of biochemical evidence prevents us from unambiguously assigning them a pathogenic role, the sheer number of potentially non-silent variants, 49, makes a strong argument for updating the evidence supporting their candidacy and clearly indicates that, indeed, many more deafness inducing variant sites exist in mt-rRNA in addition to the two ototoxic variants.

Initially, we attempted to introduce a scoring system, as in our previous HIA studies, in which comparative analysis of heterologous ribosomal structures was used to dissect the disruptive potential of extremely rare mt-rRNA mutations (Smith et al., 2014; Elson et al., 2015a; Elson et al., 2015b; Vila-Sanjurjo et al., 2021). In those early studies, the main criterion to establish a disruptive role for a particular variant was the existence of heterologous mutagenesis data supporting such a role. Under those conditions, we felt that a meaningful scoring system could be constructed based on predictions made using heterologous evidence. However, the fact that almost the majority of the variants thought to be associated with deafness studied here mapped to non-conserved regions of the mito-ribosome made the task of assigning a score highly subjective. In the absence of a scoring system, our only criterion to accept a variant as potentially pathogenic was to ask whether it could constitute a non-silent base change, in other words, whether it had the potential to induce, at the very least, a low-profile, fidelity phenotype consistent with the type of phenotypic manifestation observed in cases of altered mito-ribosomal fidelity (Vila-Sanjurjo et al., 2023).

To understand the pathogenic phenotypes induced by altered mito-ribosomal fidelity, we first must take a look at the lessons provided by the two ototoxic mutations, 847C>U (m.1494C>U) and 908A>G (m.1555A>G) of 12S mt-rRNA. Positions 847C (m.1494C) and 908A (m.1555A) normally form a C•A mismatch at the penultimate helix of human mt-12S rRNA (Figure 1A), which is replaced by a Watson:Crick canonical base pair as a result of either variant. This area of the mito-ribosome is equivalent to the bacterial AG-binding site, a region of the ribosome closely implicated in the maintenance of translational fidelity. Not surprisingly, the pathogenic effects elicited by these variants have been largely interpreted in terms of altered mito-ribosomal fidelity (Weiss-Brummer and Huttenhofer., 1989; Hobbie et al., 2008a; Hobbie et al., 2008b; He et al., 2013). Notably, despite their pathogenicity, both variants are well-tolerated and may go unnoticed in the absence of external stimuli, such as AG exposure (Fischel-Ghodsian., 1999; Bitner-Glindzicz et al., 2009; Vandebona et al., 2009). Recent work has shown that this might very well be the case for other deafness-associated mutations affecting the mito-ribosome. When a predicted error-prone mutation mapping to protein MRPS5/uS5m was introduced in a human cell line, it conferred mito-ribosomal misreading in an organello translation system (Akbergenov et al., 2018). Homozygous knock-in mutant Mrps5 mice carrying this mutation had impaired mitochondrial function in post-mitotic cells *in vivo* and showed heightened susceptibility to noise trauma (Akbergenov et al., 2018). However, much like the ototoxic mt-rRNA variants, no overt non-cochlear pathology was apparent in MRPS5/uS5m mutant mice *in vivo* (Akbergenov et al., 2018). Additionally, the authors observed the coordinated upregulation of cytosolic ribosomal proteins, an effect that had also been seen as a manifestation of the 908A>G (m.1555A>G) variant (Bykhovskaya et al., 2009; Akbergenov et al., 2018). Thus, the two known mito-ribosomal error-prone mutations for which biochemical data are available, the error-prone MRPS5/uS5m mutation and the 908A>G (m.1555A>G) variant, share several phenotypic manifestations: acute cochlear pathology in the presence of an external stimulus, decreased mito-ribosomal fidelity, coordinated upregulation of cytosolic ribosomal proteins, and a lack of overt non-cochlear pathology *in vivo* (Hu et al., 1991b; Prezant et al., 1993; Guan et al., 2000; Hobbie et al., 2008a; Hobbie et al., 2008b; Akbergenov et al., 2011; Akbergenov et al., 2018). The fact that the two 12S mt-rRNA ototoxic variants and the MRPS5/uS5m mutation display similar, low-profile fidelity phenotypes strongly suggests that the ear-damaging mechanism must be the same. Notably, low-profile phenotypes were also observed when error-prone mutations mapping to MRPS12/uS12m were generated in mice, albeit no deafness-related symptoms were reported in this case (Ferreira et al., 2019).

Despite the differences between bacterial and mitochondrial ribosomes, many lessons can be learned from studies performed with the former particles. For example, studies with bacterial and yeast ribosomes showed that impaired ribosomal fidelity could be induced by mutations mapping to many different regions of both ribosomal subunits, making altered ribosomal fidelity the most common phenotype elicited by ribosomal mutations (Agarwal et al., 2011; Datta et al., 2021; Gregory et al., 2001; Sun et al., 2011; Liiv and O'Connor., 2006; Gupta et al., 2013; Tran et al., 2011, O'Connor., 2007; O’Connor et al., 1997; O’Connor, 2007 McClory et al., 2010; Maisnier-Patin et al., 2002; Synetos et al., 1996). The existence of low-profile phenotypes associated with fidelity defects has also been observed in bacterial and yeast ribosomes, where the existence of fidelity mutations that are otherwise silent has been well-documented (O’Connor et al., 1997; Agarwal et al., 2011; Sun et al., 2011; Tran et al., 2011). This is in agreement with the idea that altered ribosomal fidelity can often be well tolerated. In addition to these low-profile phenotypes, more dramatic cases exist in which altered fidelity is just one of the molecular defects induced by rRNA mutations in bacteria and yeast (Agarwal et al., 2011; Datta et al., 2021; Gregory et al., 2001; Sun et al., 2011; Liiv and O'Connor., 2006; Gupta et al., 2013; Tran et al., 2011, O'Connor., 2007; O’Connor et al., 1997; O’Connor and Dahlberg, 1995; McClory et al., 2010; Maisnier-Patin et al., 2002).

Although the link between mt-rRNA variants and mito-ribosomal fidelity is somewhat clear, understanding how the variants lead to disease is a much more complex matter. Some recent studies in which mito-ribosomal fidelity mutants were generated by altering mito-ribosomal proteins MRPS5/uS5m and MRPS12/uS12m have just provided a first glimpse into the complex phenotypes elicited by mitochondrial mistranslation (Ferreira et al., 2019; Scherbakov et al., 2020; Richman et al., 2021; Shcherbakov et al., 2021). We have extensively reviewed this issue in an accompanying study by Vila-Sanjurjo et al. (2023).

This new appreciation of the phenotypic manifestation of mito-ribosomal fidelity mutations and its relationship to deafness provides a new theoretical framework to analyze the pathogenicity of mt-rRNA variants (reviewed by Vila-Sanjurjo et al., 2023). At the same time, the realization that low-profile phenotypes can be responsible for mitochondrially induced deafness leads to the inescapable conclusion that there must exist an important number of unknown mt-rRNA sites capable of harboring additional ototoxic variants. Our finding that 49 mt-rRNA variants were identified in patients with hearing loss is in good agreement with this idea.

Out of the 49 potentially non-silent variants, 28 possibly affected quaternary structure clearly reflecting the much larger implication of proteins in maintaining mito-ribosomal structure relative to bacterial ribosomes (Amunts et al., 2015; Greber et al., 2015). Although the phenotypic effects of disrupting the quaternary mito-ribosomal interactions cannot be further assessed, the detection of pathogenic mutations in up to 16 mito-ribosomal proteins attests to the importance of such interactions in maintaining proper mito-ribosomal function (Sanchez et al., 2021). Within the group of variants possibly affecting the quaternary structure, we were surprised by the number of variants mapping to the vicinity of protein MRPS12/uS12m, a total of 11, 10 of which are potentially disruptive (Supplementary Table S1, code: M; see also Figures 2B, C). Ribosomal protein S12 has been intimately linked to ribosomal fidelity since the classical studies in bacteria of GORINI and KATAJA (1964). More recent work uncovered the existence of a large number of fidelity mutations, sparsely distributed throughout the primary sequence of bacterial S12 (Gregory et al., 2001; Agarwal et al., 2011; Datta et al., 2021). The high structural homology existing between *E. coli* S12 and MRPS12/uS12m (Figure 2A, and Figure 2 in Vila-Sanjurjo et al., 2023) permits the extrapolation to mitochondria of the general conclusions regarding the involvement of MRPS12/uS12m in mito-ribosomal fidelity obtained in heterologous systems. Taken at first sight, the bacterial results indicate that small changes in the structure of S12 orthologues have a high probability of resulting in fidelity phenotypes, many of them well-tolerated. Direct demonstration for such a phenotype in the mammalian mitochondrial context has been obtained in a mice model of mito-ribosomal fidelity, in which both error-prone and hyper-accurate mutations were introduced in MRPS12/uS12m (Ferreira et al., 2019). Another implication of the large number of fidelity mutation sites spread throughout bacterial S12 (Gregory et al., 2001; Agarwal et al., 2011; Datta et al., 2021) is that similar phenotypes should, in principle, be induced by altering the SSU rRNA residues in direct contact with S12 orthologues. Hence, the mapping of 11 potentially disruptive variants to the neighborhood of protein MRPS12/uS12m could be tentatively interpreted as further proof of the involvement of mito-ribosomal fidelity in the etiology of mt-rRNA-induced deafness. Notwithstanding the high structural homology between *E. coli* S12 and MRPS12/uS12m, one should not necessarily expect that the exact location of bacterial fidelity sites can be directly extrapolated to the mitochondrial context, as small positional differences might exist. Evidence to support this view has been recently provided by Juskeviciene et al. (2022) by showing the existence of positional differences between the relevant *E. coli* and mitochondrial mutants mapping to MRPS5/uS5m.

This work also highlights the potential involvement of non-conserved and even haplotype-associated mt-rRNA variants in human diseases. It can be argued that the well-known deafness variants 847C>U (m.1494C>U) and 908A>G (m.1555A>G) affect structurally non-conserved positions, forming a mismatch in the human mito-ribosome *versus* a canonical base pair in the bacterial ribosome. Despite this, these two variants map to a region whose overall structure and function are well-defined. The results presented here underscore, however, the potential deleterious effect of variants mapping to mitochondria-exclusive regions that have no equivalent in non-mitochondrial ribosomes. The degree of conservation of mt-rRNA variants has been frequently used as a proxy of their pathogenicity, even when such conservation was estimated by considering extremely narrow slices of the phylogenetic tree (*i.e.*, mammalian or vertebrate mt-rRNAs) (Li et al., 2004; Chaig et al., 2008; Zhu et al., 2009; Lu et al., 2010; Rydzanicz et al., 2010; Mutai et al., 2011; Shen et al., 2011; Padma et al., 2012; Smith et al., 2014). This type of inference might lead to error, as shown by the case of the **76A>C (m.723A>C)** variant. Position **76A (m.723A)** is involved in a triple base interaction that brings together helices h7 and h12 near the recognition sites for MRPS16/bS16 and MRPS25/mS25, both known to harbor pathogenic mutations (Figure 5A and Supplementary Figure S7) (Miller et al., 2004; Bugiardini et al., 2019; Sanchez et al., 2021). The potential for pathogenicity is clear, as **76A>C (m.723A>C)** is expected to disrupt this triple base pair interaction, possibly affecting the helical arrangement of part of 12S mt-rRNA and the binding of MRPS16/bS16 and MRPS25/mS25. However, the triple base interaction is not conserved, as it is absent in the *S. scrofa* mito-ribosome (Greber et al., 2015). This example highlights the potentially disruptive role of base changes at non-conserved residues.

The possibility that haplotype context could affect the manifestation of mt-rRNA variants was recently proposed by our group (Vila-Sanjurjo et al., 2021) and has received an important degree of support from this work. A total of 33 haplotype-defining variants were analyzed, of which 17 were deemed potentially non-silent (Supplementary Table S1). The case of the 806A>G (m.1453A>G), itself a non-haplotype marker, is of particular interest (Rydzanicz et al., 2010; Padma et al., 2012). Position 806A (m.1453A) forms a U:A Watson:Crick base pair with 790U (m.1437U) in helix h43 of 12S mt-rRNA (Figure 9F and Supplementary Figure S17A). The A>G base change at this position would replace the Watson:Crick base pair with a U•G wobble. Immediately preceding the 790U:806A (m.1437U:m.1453A) in the 2.2-Å cryo-EM structure lies the G•U wobble base pair formed by the haplotype marker 791G (m.1438G) and position 805U (m.1452U). The Cambridge reference sequence carries an A at position 791 (m.1438), which is present in only 5% of the GenBank sequences and would replace the G•U wobble with an A:U Watson:Crick base pair (Supplementary Table S1) (Andrews et al., 1999; Ruiz-Pesini et al., 2007). These data indicate that the wobble and Watson:Crick geometries must be tolerated at the base pair formed by 791 (m.1438) and 805 (m.1452). In the case of the 806A>G (m.1453A>G) variant, if preceded by the 791G•805U (m.1438G•1452U) wobble observed in the structure and present in 95% of the available GenBank sequences (Ruiz-Pesini et al., 2007), a highly non-isosteric U•G/G•U tandem wobble would be created (Ananth et al., 2013). In contrast, in the context of a Watson:Crick 791A:805U (m.1438A:1452U) base pair (present in 5% of the available GenBank sequences), an intra-helical 790U•806G (m.1437U•m.1453G) wobble would cause little disruption (Ananth et al., 2013). As the patient belonged to haplogroup V, their mtDNA harbored the 791G (m.1438G) variant (Rydzanicz et al., 2010), thus giving rise to the more disruptive configuration. The distance from 805U (m.1452U) to the pathogenic site at position 108 of MRPS14/uS14m is only ∼15-Å (not shown) (Jackson et al., 2019), hence demonstrating the potential for this region to harbor disruptive base changes. For all these reasons, we believe that the case of the 806A>G (m.1453A>G) variant constitutes a good example of how the haplotype context might lead to a non-silent phenotype and would, if confirmed, constitute the first example of such an effect in mt-rRNA.

As mentioned in the introduction, AGs antibiotics are an important class of external agents capable of affecting the penetrance of mt-rRNA variants. HIA studies conducted by our group with highly rare mt-rRNA variants reportedly in association with diseases showed that an important number of them mapped to the vicinity of the binding sites of known ribosomal antibiotics (Smith et al., 2014; Elson et al., 2015a). As a result, we proposed that antibiotic hyper-susceptibility, as reported for the 908A>G (m.1555A>G) and 847C>U (m.1494C>U) variants, could be responsible for the phenotype elicited by an unknown number of such potentially pathogenic mt-rRNA mutations. Although we did not identify any variant mapping to the vicinity of any known antibiotic binding site in this work, the drastically different architecture of the mito-ribosome, relative to its bacterial and cytoplasmic counterparts, provides ample opportunities for the binding of yet unidentified small molecules. As an example of this, a molecule of NAD has been modeled in the SSU of the 2.2-Å cryo-EM human mito-ribosomal structure, not too far from MRPS12/uS12m. This NAD molecule establishes a water-mediated interaction with the site of variation **401C (m.1048C)** (Figure 3D) (Itoh et al., 2021; Itoh et al., 2022). The presence of co-factors associated with the respiratory complexes in mito-ribosomes, such as NAD, has been previously reported in fungal mito-ribosomes, leading to the proposal that the binding of NAD could have a regulatory role by linking mt-rRNA assembly to local NAD levels (Itoh et al., 2020). Despite this possibly important role, whether the water-mediated interaction between **401C (m.1048C)** and the NAD molecule is of any relevance to mitochondrial translation remains unknown.

The growing realization in the field that mtDNA disease and, more specifically, deafness can be brought about by variants with low-profile phenotypes, such as those affecting mito-ribosomal fidelity (reviewed in accompanying study by Vila-Sanjurjo et al., 2023), effectively sets up a low phenotypic threshold for pathogenic mt-rRNA variants, as argued here. This does not rule out, however, that variants with stronger disruptive power were present in the collection analyzed in this work. Indeed, we have characterized several variants capable of disrupting the interaction of the mito-ribosome and its ligands, such as translation factors, tRNAs, and mRNA, as well as others affecting the function of inter-subunit bridges. These variants are expected to exert a negative effect on translation that can go well beyond fidelity. In these cases, there usually exists relevant information from heterologous sources to score them as likely pathogenic. According to our predictions, variant **910A>C (m.1557A>C)** is a good example, as it has the strongest disruptive potential. Base changes at position **910A (m.1557A)** are expected to completely abolish mito-ribosomal translation by directly interfering with genetic decoding (Yoshizawa et al., 1999; Abdi and Fredrick., 2005; Cochella et al., 2007). Unfortunately, no details were provided on the isolation of this variant, leaving us unable to determine how mitochondrial translation could be supported in the proband (Tazetdinov and Dzhemileva, 2007).

In summary, we have provided a much-needed assessment of the structural and functional role of suspect mt-rRNA variants identified in the context of deafness. Besides highlighting the key role of the primary sequence of mt-rRNA in the etiology of deafness, this research provides a new framework to understand how mt-rRNA variants, particularly those located at non-conserved positions, may lead to deafness. Hence, the results presented here constitute an important push toward the final elucidation of the role of mt-rRNA in mitochondrial deafness and mitochondrial disease. Such elucidation will eventually require biochemical evidence obtained with mutant mitochondria carrying mito-ribosomes with altered mt-rRNAs.

## Materials and methods

Structural analysis was performed with Chimera X (Pettersen et al., 2021), essentially as described (Elson et al., 2015b) using the 2.2-Å cryo-EM human mito-ribosomal structure (RCSB Protein Data Bank ID: 8ANY) (Itoh et al., 2021; Itoh et al., 2022). All other ribosomal structures used in this work (RCSB Protein Data Bank IDs: 5AJ4, 7NSH, 6VMI, 6ZM6, and 6RW4) were previously superposed onto the 2.2-Å mito-ribosomal structure with the Matchmaker utility of Chimera X (Pettersen et al., 2021), using protein S12 as a reference chain. Hydrogen bonds were identified with the H-bonds utility of Chimera X with distance and angle tolerances of 0.400Å and 20°, respectively (Pettersen et al., 2021). The following datasets were used in this work: 8ANY (Itoh et al., 2021, Itoh et al., 2022), 6VMI (Koripella et al., 2020), 5AJ4 (Greber et al., 2015), 6WR5 (Khawaja et al., 2020), and 4YBB (Noeske et al. 2015).

## Data Availability

The datasets presented in this study can be found in online repositories. The names of the repository/repositories and accession number(s) are as follows: RCSB Protein Data Bank: 8ANY, 6VMI, 5AJ4, 6WR5, and 4YBB.
