# Supplementary material for: Structural analysis of mitochondrial rRNA gene variants identified in patients with deafness
Source: Front Physiol. 2023 Jun 8;14:1163496. doi: 10.3389/fphys.2023.1163496 (PMC10285412; doi:10.3389/fphys.2023.1163496)
Supplement: Supplementary file 1 [file DataSheet1.pdf]

Structural analysis of deafness-related mtDNA variants mapping to mitochondrial rRNA genes

Antón Vila-Sanjurjo[1], Natalia Mallo[1], Joanna L. Elson[2,3], Paul M. Smith[4], Emma L. Blakely[5,6], & Robert W. Taylor[5,6]

Supplementary Information

Table of Contents

Supplementary information........6
Silent variants........6
SSU mt-rRNA........6
-74U>C (721U>C) and 105C>U (m.752C>U):........6
-88A>G (m.735A>G):........6
-154A>G (m.801A>G):........6
166A>G (m.813A>G):........7
-192A>G (m.839A>G):........7
-309delC (m.956delC), 312C>U (m.959C>U), 313delC (m.960delC), 313Ins (m.960ins), 314delT+insC(n) (m.961delT+insC(n)), 314insC (m.961insC), 314U>G (m.961U>G), 314U>C (m.961U>C), 314U>A n(m.961U>A), 315delC (m.962delC), and 394A>G (m.1041A>G):.......7
-371G>A (m.1018G>A):........8
-500G>A (m.1147G>A):........8
-507A>C (m.1154A>C):........8
-644U>C (m.1291U>C):........9
-727A>G (m.1374A>G):........9
-758C>U (m.1405C>U) and 759U>C (m.1406U>C):........9
-773U>C (m.1420 U>C):........9
-870A>C (m.1517A>C) 883A>G (m.1530A>G):........10
LSU mt-rRNA........10
-30U>C (m.1700U>C), 38A>U (m.1708A>U), and 49G>A (m.1719G>A):........10
-(m.1811A>G) 141A>G:........11
-399U>C (m.2069U>C):........11
-568A>G (m.2238A>G):........11
-615U>C/G (m.2285U>C/G):........11
-964U>C (m.2634U>C):........12
-1543A>G (m.3213A>G):........12
Unclear variants........13

-220C>U (m.867C>U): 13
-380A>G (m.1027A>G): 13
Supplementary Figures 14
Supplementary Figure S1 15
Supplementary Figure S2 17
Supplementary Figure S3 18
Supplementary Figure S4 20
Supplementary Figure S5 21
Supplementary Figure S6 23
Supplementary Figure S7 25
Supplementary Figure S8 26
Supplementary Figure S9 27
Supplementary Figure S10 28
Supplementary Figure S11 29
Supplementary Figure S12 30
Supplementary Figure S13 32
Supplementary Figure S14 34
Supplementary Figure S15 36
Supplementary Figure S16 38
Supplementary Figure S17 40
Supplementary Figure S18 41
Supplementary Figure S19 43
Supplementary Figure S20 44
Supplementary Figure S21 45
Supplementary Figure S22 47
Supplementary Figure S23 48
Supplementary Figure S24 49
Supplementary Figure S25 50
Supplementary Figure S26 52
Supplementary Figure S27 54
Supplementary Figure S28 55
Original description of putatively non-silent variants in the literature 56
References 66

Small Subunit					
Genomic	Gene	Base change		Haplogrpoup marker	Codes $
669	22	T-C	(Rydzanicz et al., 2010, Leveque et al., 2007, Rydzanicz et al., 2009, Elstner et al., 2008)	N	2,4,M,P
709	62	G-A	(Elstner et al., 2008, Guaran et al. 2013, Konings et al., 2008, Li et al., 2005, Rydzanicz et al., 2010)	Y	3,4,P
710	63	T-C	(Konings et al., 2008)	Y	S
712	65	C-A	(Guaran et al., 2013)	N	3,4,P
721	74	T-C	(Guaran et al., 2013, Elstner et al., 2008, Rydzanicz et al., 2010, Konings et al., 2008)	N	S
723	76	A-C	(Konings et al., 2008, Rydzanicz et al., 2010)	N	3,4,P
735	88	A-G	(Mkaouar-Rebai et al., 2010)	N	2,4
742	95	T-C	(Guaran et al., 2013)	N	2,P
745	98	A-G	(Lu et al., 2010)	N	3,P
750	103	G-A	(Guaran et al., 2013, Konings et al., 2008, Li et al., 2005, Lu et al., 2010, Rydzanicz et al., 2010)	Y	2,4,P
752	105	C-T	(Li et al., 2005)	N	S
769	122	G-A	(Elstner et al., 2008, Konings et al., 2008)	Y	3,P
770	123	C-T	(Konings et al., 2008)	N	S
786	139	G-A	(Guaran et al., 2013)	N	2,4,M,P
792	145	C-T	(Yano et al., 2014, Lu et al., 2010)	N	2,4,P
801	154	A-G	(Lu et al., 2010)	N	S
813	166	A-G	(Guaran et al., 2013, Rydzanicz et al., 2010)	Y	S
825	178	T-A	(Konings et al., 2008)	Y	3,4,M,P
827	180	A-G	(Rydzanicz et al., 2010, Konings et al., 2008, Human et al., 2010, Yano et al., 2014, Li et al., 2004, Xing et al., 2006, Li et al., 2005, Chaig et al., 2008)	Y	3,4,M,P
839	192	A-G	(Lu et al., 2010)	N	2,4
856	209	A-G	(Yano et al., 2014, Lu et al., 2010)	N	2,4,P
867	220	C-T	(Guaran et al., 2013)	N	U
930	283	G-A	(Guaran et al., 2013, Konings et al., 2008,Rydzanicz et al., 2010)	Y	4,M,P
942	295	A-G	(Guaran et al., 2013, Lu et al., 2010)	N	3,M,P
951	304	G-A	(Elstner et al., 2008, Guaran et al., 2013, Konings et al., 2008, Rydzanicz et al., 2010, , Lu et al., 2010, Igumnova et al., 2019)	Y	2,4,M,P
956	309	delC	(Konings et al., 2008)	N	S
959	312	C-T	(Guaran et al., 2013)	N	S
960	313	delC	(Elstner et al., 2008, Lu et al., 2010)	N	S
960	313	Ins	(Elstner et al., 2008)	Y	S
961	314	delT+insC(n)	(Human et al., 2010)	N	S

961	314	ins(C)	(Mkaouar-Rebai et al., 2008, Rydzanicz et al., 2009, Igumnova et al., 2019)	N	S
961	314	T-G	(Human et al., 2010, Elstner et al., 2008, Igumnova et al., 2019)	Y	S
961	314	T-C	(Rydzanicz et al., 2009, Yano et al., 2014, Li et al., 2005, Lu et al., 2010)	Y	S
961	314	T-A	(Igumnova et al., 2019)	N	S
962	315	delC	(Human et al., 2010)	N	S
980	333	T-C	(Guaran et al., 2013)	Y	2,B
988	341	G-A	(Konings et al., 2008, Rydzanicz et al., 2010)	N	2,4,L,I
990	343	T-C	(Konings et al., 2008)	N	2,4,L,I
1005	358	T-C	(Li et al., 2005, Yano et al., 2014)	Y	3,4,B
1007	360	G-A	(Elstner et al., 2008)	N	2,B
1008	361	A-G	(Konings et al., 2008)	N	2,B
1018	371	G-A	(Elstner et al., 2008, Konings et al., 2008)	Y	S
1027	380	A-G	(Lu et al., 2010)	N	U
1041	394	A-G	(Konings et al., 2008)	Y	S
1047	400	A-G	(Rydzanicz et al., 2010)	N	4,M,P
1048	401	C-T	(Konings et al., 2008)	Y	4,M,P
1095	448	T-C	(Yano et al., 2014, Tessa et al., 2001, Zhao et al., 2004b, Muyderman et al., 2012)	N	2,B,I
1106	459	C-T	(Konings et al., 2008)	N	4,I,M,P
1107	460	T-C	(Li et al., 2005)	Y	S,M,P
1116	469	A-G	(Lu et al., 2010)	Y	3,4,P
1118	471	A-G	(Guaran et al., 2013)	N	4,P
1119	472	T-C	(Guaran et al., 2013, Li et al., 2005)	Y	S
1147	500	G-A	(Farhadi et al., 2016)	N	S
1154	507	A-C	(Konings et al., 2008)	N	S
1180	533	T-G	(Li et al., 2004)	N	2,L
1189	542	T-C	(Guaran et al., 2013, Konings et al., 2008, Rydzanicz et al., 2010, Lu et al., 2010, Meza et al., 2011)	Y	3,D,I
1192	545	C-A	(Lu et al., 2010)	N	2,D,I
1192	545	C-T	(Lu et al., 2010)	N	2,D,I
1193	546	T-C	(Guaran et al., 2013, Padma and Ramchander., 2008)	Y	S
1226	579	C-G	(Li et al., 2004)	N	2,3,L
1243	596	T-C	(Ballana et al., 2006b, Guaran et al., 2013, Konings et al., 2008, Rydzanicz et al., 2010,)	Y	2,4,P
1291	644	T-C	(Ballana et al., 2006b, Ballana et al., 2006a, Abreu-Silva et al., 2006)	N	S
1310	663	C-T	(Yano et al., 2014)	N	2,4,P
1331	684	A-G	(Lu et al., 2010)	N	3,L
1374	727	A-G	(Lu et al., 2010)	N	S
1382	735	A-C	(Li et al., 2005)	Y	2,4,P
1405	758	C-T	(Guaran et al., 2013)	N	S
1406	759	T-C	(Rydzanicz et al., 2010, Konings et al., 2008, Guaran et al., 2013)	Y	S
1420	773	T-C	(Li et al., 2005)	N	S

1438	791	G-A	(Guaran et al., 2013, Konings et al., 2008, Li et al., 2005, Rydzanicz et al., 2010)	Y	**S**
1443	796	T-C	(Li et al., 2005)	N	**3,4,P**
1452	805	T-C	(Lu et al., 2010)	Y	**S**
1453	806	A-G	(Rydzanicz et al., 2010, Padma et al., 2012)	N	**2,P**
1462	815	G-A	(Elstner et al., 2008, Konings et al., 2008, Lu et al., 2010, Padma et al., 2012)	Y	**2,D**
1473	826	C-T	(Chen et al., 2018)	N	**2,L**
1503	856	G-A	(Rydzanicz et al., 2010, Chen et al., 2011)	Y	**B**
1508	861	C-T	(Padma et al., 2012)	N	**2,B**
1517	870	A-C	(Mkaouar-Rebai et al., 2008)	N	**S**
1525	878	C-G	(Smith et al., 2014)	N	**4,P**
1530	883	A-G	(Konings et al., 2008)	N	**S**
1537	890	C-T	(Leveque et al., 2007, Konings et al., 2008, Estivill et al., 1998a)	N	**2,3,B**
1557	910	A-C	(Tazetdinov et al., 2007)		**L**
1598	951	G-A	(Li et al., 2005)	Y	**3,4,L,I**
**Large Subunit**					
**Genomic**	**Gene**	**Base change**		**Haplogrpoup marker**	**Codes $**
1700	30	T-C	(Guaran et al., 2013)	Y	**S**
1708	38	A-T	(Guaran et al., 2013)	N	**S**
1719	49	G-A	(Guaran et al., 2013)	Y	**S**
1811	141	A-G	(Guaran et al., 2013)	Y	**S**
2069	399	U-C	(Yano et al., 2014)	N	**S**
2238	568	A-G	(Zhao et al., 2004b)	N	**S**
2285	615	T-C	(Yano et al., 2014)	N	**S**
2634	964	T-C	(Yano et al., 2014)	N	**S**
3213	1543	A-G	(Guaran et al., 2013)	N	**S**

Table S1. List of variants studied in this work. $ Codes used in Table: **2**, variant potentially affecting secondary structure; **3**, variant potentially affecting tertiary structure; **4**, variant potentially affecting quaternary structure; **S**, silent variant; **B**, variant potentially affecting bridge function; **L**, variant potentially affecting ligand binding; **D**, variant potentially affecting a conformationally dynamic region; **I**, variant potentially affecting initiation; **M**, variant in the neighborhood of MRPS12; **P**, variant potentially affecting interaction with early binding mt-r-prot. or with mt-r-prot. known to harbor pathogenic mutations; **U**, unclear variant.

Supplementary information

Silent variants

SSU mt-rRNA

-74U>C (721U>C) and 105C>U (m.752C>U):

The **74U>C(721U>C)** base change has been recurrently found associated with hearing impairment (Guaran et al., 2013, Elstner et al., 2008, 2004, Rydzanicz et al., 2010, Konings et al., 2008). This base change is moderately abundant with 167 GenBank appearances to date (Ruiz-Pesini et al., 2007). Position **74U (721U)** is unpaired and stacks onto position **105C>U (m.752C>U)**, where a C>U base change has been found in a patient with sensorineural hearing loss (Li et al., 2005). The fact that neither position uses its base to make contacts to other mito-ribosomal residues argues against their potential pathogenicity.

-88A>G (m.735A>G):

This variant was detected by Mkaouar-Rebai et al. in two unrelated probands with sensorineural hearing loss (Mkaouar-Rebai et al., 2010). In the secondary structure map of 12S mt-rRNA, position **88A (m.735A)** lies in the capping loop of h7. The loop was not resolved in the original structure of the human ribosome (3J9M) but could be modeled in the 2.2-Å structure of the mito-ribosome, albeit at a lower resolution, likely indicating high flexibility in this region. Despite the lack of resolution in the region, the unpaired **88A (m.735A)** has been modeled sandwiched between Arg 68 of MRPS26/mS26 (pink in the structure) and the adjacent 89C (m.736C) is also in the neighborhood of the closing loop of h7 but far from the base of **88A (m.735A)**. All these observations, together with the lack of hydrogen bonding interactions involving the base of **88A (m.735A)**, strongly suggest that the G>A base change at **88A (m.735A)** is phenotypically silent.

-154A>G (m.801A>G):

The **154A>G (m.801A>G)** highly rare variant was found by Lu et al. (Lu et al., 2010) in a pediatric patient with hearing loss. Position **154A (m.801A)** forms a non-canonical A●A base pair with 67A (m.714A) at the junction between h6a and h14 (not shown). The base pair, together with the preceding triple base interaction involving the 153C:68G (m.800C:m.715G) base pair in h6a and position 169A (m.816A), located in the rRNA segment linking h14 and h5, likely aids in maintaining the higher order structure at the junction of these three rRNA helices. The A●A base pair is replaced in *S. scrofa* by a G●A mismatch (RCSB ID: 5AJ4), suggesting the existence of enough flexibility in this helical region to tolerate slight deviations in the relative orientation of the bases forming this base pair. This view would be in agreement with the idea that the **154A>G (m.801A>G)** replacement, resulting in an A●G mismatch, could be easily tolerated.

166A>G (m.813A>G):

Identified in two studies (Guaran et al., 2013, Rydzanicz et al., 2010). Adjacent to bridge B5, which involves residues 164 (m.811) and 165A (m.812A) in 12S mt-rRNA and MRPL14/uL14m in the LSU (Amunts et al., 2015). Position **166A (m.813A)**, stacks onto the adjacent base of **166A (m.813A)** and is too far to interact with other mito-ribosomal residues or ligands. Despite its location near a functionally important mito-ribosomal element, there is no evidence that the base change might induce a structural clash.

-192A>G (m.839A>G):

The **192A>G (m.839A>G)** variant was found in a patient with non-syndromic hearing loss (Lu et al., 2010). Position **192A>G (m.839A>G)** is base paired to 203U (m.850U) within h15. Two hydrogen bonds from the RNA backbone at position **192A (m.839A)** to positions 21 and 84 of MRPS16/bS16 are visible in the structure. The base pair is substituted with a G•U wobble in the *S. scrofa* (RCSB ID: 5AJ4), a fact that argues in favor of a non-deleterious effect associated to the **192A>G (m.839A>G)** variant.

-309delC (m.956delC), 312C>U (m.959C>U), 313delC (m.960delC), 313Ins (m.960ins), 314delT+insC(n) (m.961delT+insC(n)), 314insC (m.961insC), 314U>G (m.961U>G), 314U>C (m.961U>C), 314U>A n(m.961U>A), 315delC (m.962delC), and 394A>G (m.1041A>G):

Several deafness-associated variants mapping to the single-stranded segments linking helices h20 and h22 have been identified. The involved residues are **309C (m.956C)**, **312C (m.959C)**, **313C (m.960C)**, **314U (m.961U)**, **315C (m.962C)**, and **394A (m.1041A)** (Guaran et al., 2013, Elstner et al., 2008, Rydzanicz et al., 2010, Li et al., 2005, Lu et al., 2010, 2010, Human et al., 2010, Yano et al., 2014, Rydzanicz et al., 2009, Mkaouar-Rebai et al., 2008, Igumnova et al., 2019). The fact that all these residues map to the back of the subunit, strongly suggests a lack of involvement in mito-ribosomal function. In agreement with this idea, the lack of density surrounding all of these positions, except for **309C (m.956C)** and **394A (m.1041A)**, indicates that the structure of this RNA segment is highly disordered and flexible. Hence, according to the available structural data, there is no reason to think that any of the base changes reported at positions **312C (m.959C)**, **313C (m.960C)**, **314U (m.961U)**, and **315C (m.962C)** could not be silently accommodated in the structure. Indeed, two of the reported variants, namely **313Ins (m.960ins)** and **314U>G (m.961U>G)** are known haplotype markers. The **312C>U (m.959C>U)** variant was found together with the LSU **49G>A (m.1719G>A)** base change and the 35delG allele of the connexin26 gene GJB2 in heteroplasmy (Guaran et al., 2013). Since both **312C>U (m.959C>U)** and the LSU variant **49G>A (m.1719G>A)** are likely polymorphisms (see below), the possibility that the GJB2 35delG mutation may be the only cause of deafness in this patient appears as the most probable explanation for the observed symptoms (Guaran et al., 2013, Estivill et al., 1998).

Even the variants identified at the two ordered bases residues above, namely **309C (m.956C)** and **394A (m.1041A)** are expected to be polymorphisms. Position **309C (m.956C)** (Konings et

al., 2008) is part of a stretch of five consecutive Cs, only two of which are clearly ordered. As for the **394A>G (m.1041A>G)** variant (Konings et al., 2008), lying on the RNA strand opposite to the run of residues 309-15 (m.956-62), its base is stacked onto the guanidinium group of Arg 123 of MRPS15/uS15. Without any other involvement in structural stabilization, an A>G base change should, in principle, be well tolerated at this position. Despite all the presented evidence, the concentration of putatively pathogenic mutations in this region of 12S mt-rRNA is intriguing and perhaps reflects the existence of low selective pressure against mutations in this region.

-371G>A (m.1018G>A):

Two variants map to the neighborhood of helix h23a. The haplotype marker **371G>A (m.1018G>A)** variant was identified by several authors in a hearing impaired patient (Elstner et al., 2008, Konings et al., 2008). In the secondary structure map of 12S mt-rRNA, **371G (m.1018G)** maps to the single-stranded stretch joining h23 and h23a. The base of **371G (m.1018G)**, together with the adjacent residues (positions 369-70 (m.1016-7)), is involved in a network of water-mediated hydrogen bonds that stabilizes the highly contorted conformation of the single-stranded stretch. This unusual conformation creates sites of recognition for proteins MRPS18C/bS18c (orange in structure), MRPS21/bS21 (light green in structure), and MRPS37/mS37 (color cornflower blue in structure). The equivalent position in the *S. scrofa* mito-ribosome is an A (RCSB ID: 5AJ4), strongly suggesting that the G>A variant at **371 (m.1018)** is silent.

-500G>A (m.1147G>A):

The **500G>A (m.1147G>A)** variant was found in an Iranian individual with hearing loss and directly submitted to MITOMAP (Farhadi 2016, Ruiz-Pesini et al., 2007). Position **500G (m.1147G)** forms a G•U wobble with position 511U (m.1158U), located at the distal helical portion of h27. A hydrogen bond between MRPS38/mS38 Arg 161 (orange in the structure) and the RNA backbone at the adjacent 499C (m.1146C) is observed. Protein MRPS38/mS38 is part of the functionally important bridge mB4 (see below). The **500G>A (m.1147G>A)** variant would result in the substitution of a G•U wobble with a Watson-Crick A:U at the end of a helical segment, which in principle should be well tolerated (Ananth et al., 2013). Support for this idea comes from the fact that even a geometrically divergent U●U base pair with two hydrogen bonds is present in the *S. scrofa* mito-ribosome in place of the human Watson: Crick configuration of the **500G:**511U **(m.1147G:**1158U**)** base pair (Greber et al., 2015). Hence, the **500G>A (m.1147G>A)** variant is regarded as silent, in agreement with a previous report (Haumann et al., 2020).

-507A>C (m.1154A>C):

The **507A>C (m.1154A>C)** variant was identified in the 12S rRNA of a patient with paternally inherited hearing loss (Konings et al., 2008) and analyzed by HIA by our group before the arrival of high-resolution structures of the mammalian mito-ribosome (Smith et al., 2014). The *E. coli* equivalent to **507A (m.1154A)** is A901 (Smith et al., 2014). Position **507A (m.1154A)** is located in intersubunit bridge mB3, involving helices h27 and H67 of 12S and 16S mt-rRNA,

respectively (Amunts et al., 2015). Bridge mB3 is the pivot of inter-subunit movement and remains intact during rotation (Amunts et al., 2015). Two clear hydrogen bonds are observed from Arg 144 and Arg 155 of MRPS38/mS38 (orange in the structure) to the RNA backbone at position **507A (m.1154A)**, whereas the base of this residue is stacked under Arg 151. Protein MRPS38/mS38 is part of the neighboring bridge mB4, a mitochondrial-specific bridge that buries the largest amount of surface area of all mito-ribosomal bridges (Amunts et al., 2015). Perhaps the presence of MRPS38/mS38 in the mitochondrial ribosome explains the structural differences between the bacterial and mitochondrial distal end of h27. For example, the bacterial heterologous equivalent to **507A (m.1154A)**, position A901, is involved in tertiary contacts within helix b-h27 (Smith et al., 2014). No such contacts involving the base of **507A (m.1154A)** are observed in the human mito-ribosome (Khawaja et al., 2020, Itoh et al., 2021, Itoh et al., 2022). In light of this evidence, the bacterial mutagenesis data used to support the "likely disruptive" assignment for this mutation in our previous report has to be reconsidered (Smith et al., 2014). In particular, the fact that the *S. scrofa* mito-ribosome possesses a U at the equivalent position (505U in structure 5AJ4), clearly supports the idea that the **507A>C (m.1154A>C)** variant is likely silent, in agreement with the paternal inheritance of hearing impairment in this subject.

-644U>C (m.1291U>C):

644U>C (m.1291U>C) (Ballana et al., 2006a, Ballana et al., 2006b, Abreu-Silva et al., 2006). Position **644U (m.1291U)** is located in the single-stranded stretch connecting helices h35 and h36. The **644U (m.1291U)** base is unpaired and far away from any potential hydrogen-bonding partner. This, together with the location of this residue, internal but far away from any functional center, strongly suggests that the **644U>C (m.1291U>C)** variant is silent. The pathogenic nature of this variant has been contested (Abreu-Silva et al., 2006).

-727A>G (m.1374A>G):

The **727A>G (m.1374A>G)** variant was identified by Lu et al. (Lu et al., 2010). Position **727A (m.1374A)** is unpaired within the loop capping h41. Contact from MRPS29/mS29 to the O2' of **727A (m.1374A)** are visible. Despite this, the base change should not have any effect.

-758C>U (m.1405C>U) and 759U>C (m.1406U>C):

The base C>U base change at position **758C (m.1405C)** was identified together with 35delG/35delG in GJB2 (Guaran et al., 2013). The **759U>C (m.1406U>C)** variant was identified in association to deafness in three studies (Guaran et al., 2013, Konings et al., 2008, Rydzanicz et al., 2010). Both **758C (m.1405C)** and **759U (m.1406U)** are unpaired in the loop capping h42. No hydrogen bonds involving either base are observed, strongly suggesting that both base changes can be tolerated with no effect.

-773U>C (m.1420 U>C):

The variant **773U>C (m.1420 U>C)** was identified by Li et al. in a Chinese pediatric

subject with aminoglycoside-induced and non-syndromic hearing loss (Li et al., 2005). Position **773U (m.1420 U)** is an unpaired residue located on the single-stranded strand connecting helices h29 and h42. Positions 775G (m.1422G) and 776A (m.1423A) establish Type II and Type I minor interactions, respectively, with P-site tRNA (Lancaster and Noller., 2005). Not surprisingly, almost all mutations at the bacterial equivalents of these positions, the nearly universally conserved G1338 and A1339 blocked translation (Abdi and Fredrick., 2005). The fact that the base of **773U (m.1420 U)** is too far from all neighboring residues to establish hydrogen bonds and that an A and a C are the heterologous equivalents of this residue in the *S. scrofa* mito-ribosome and the *E. coli* ribosome, respectively strongly suggest that the **773U>C (m.1420 U>C)** variant is silent.

-870A>C (m.1517A>C) 883A>G (m.1530A>G):

Two variants map to the distal half of helix h44, specifically at positions **870A>C (m.1517A>C)** and **883A>G (m.1530A>G)** (Konings et al., 2008, 1766 Mkaouar-Rebai,E. 2008). Instead of a helical element, this region of h44 has been modeled as two stretches of unpaired RNA running in an antiparallel fashion. Both **870A (m.1517A)** and **883A (m.1530A)** are unpaired and with their bases not involved in any hydrogen bond, strongly arguing for the consideration of the two variants as silent.

LSU mt-rRNA

-30U>C (m.1700U>C), 38A>U (m.1708A>U), and 49G>A (m.1719G>A):

The **30U>C (m.1700U>C)** haplotype marker was found in association to deafness by Guaran et al. (Guaran et al., 2013). In the secondary structure map of 16S mt-rRNA, **30U (m.1700U)** maps to the long single-stranded region connecting helices H4 and H11. Two hydrogen bonds are visible from Arg 196 of MRPL47/uL29 to the RNA backbone at position **30U (m.1700U)**. However, the base of **30U (m.1700U)** does not participate in any hydrogen bonds. Replaced by a C at the equivalent position in the *S. scrofa* mito-ribosome (position 32 in RCSB ID: 7NSH). In the pig structure 32C establishes two hydrogen bonds with adenine 36A (equivalent to 34U (m.1704U) in the human mito-ribosome). Consistent with a polymorphism.

Guaran et al. found a second variant in the same single-stranded stretch, namely **38A>U (m.1708A>U)** (Guaran et al., 2013). This residue establishes two hydrogen bonds with protein MRPL37/mL37, one involving the base via its amino group and the other involving the ribose. In the *S. scrofa* mito-ribosome, **38A (m.1708A)** is replaced with a C (position 40 in RCSB ID: 7NSH), which also participates in a hydrogen bond with MRPL37/mL37. Given the distal location of the **38A>U (m.1708A>U)** variant, away from all functional centers, its pathogenic character remains unclear.

The **49G>A (m.1719G>A)** variant, mapping to the end of the single-stranded stretch connecting helices h4 and h11 was also found by Guaran et al. (Guaran et al., 2013), together with the 12S mt-rRNA C>U variant at position **312 (m.959)** and the 35delG allele of GJB2 in heteroplasmy. Residue **49G (m.1719G)** makes a sheared base pair with 339G (m.2009G). Several hydrogen bonds are observed from protein MRPL51/mL43 to the RNA backbone at

positions 339-40 (m.2009-10). A hydrogen bond from is also formed between Arg 75 of MRPL9 to the RNA backbone at position 48A (m.1718A). Position **49G (m.1719G)** is replaced by an A at position 51 of 16S mt-rRNA in the *S. scrofa* mito-ribosome, without disrupting the sheared base pair, hence arguing for the fact that **49G>A (m.1719G>A)** is a polymorphism. As mentioned above for the **312C>U (m.959C>U)** variant, the 35delG allele in GJB2 (or other unknown factor/s) is the most probable cause for the deafness observed in this patient (Guaran et al., 2013).

-(m.1811A>G) 141A>G:

The haplotype marker **(m.1811A>G) 141A>G** was identified in association with deafness by Guaran et al. (Guaran et al., 2013). The base near the back of the subunit and not involved in any hydrogen bonding interaction, underscoring its unimportant role in mito-ribosomal structure.

-399U>C (m.2069U>C):

The **399U>C (m.2069U>C)** variant was found in a Japanese patient with hearing loss (Yano et al., 2014). In the 2.2-Å structure of the human mito-ribosome, position **399U (m.2069U)** was modeled with its base pointing away from any potential hydrogen bond interaction, despite the lack of clear density around it. Nevertheless, this configuration is consistent with that observed in the structure of the *S. scrofa* mitochondrial LSU for its heterologous equivalent residue, namely position 404C (RCBS ID: 7NSH) (Kummer et al., 2021).

-568A>G (m.2238A>G):

The variant **568A>G (m.2238A>G)** was found together with the SSU **448U>C (m.1095U>C)** variant (Zhao et al., 2004b). In the secondary structure map of 16S mt-rRNA, position **568A (m.2238A)** is located in the distal single-stranded stretch connecting H41 to H43 and H44. Within this unpaired stretch, the base of **568A (m.2238A)** does not play any particular structural role besides stacking within the single RNA strand. Hence, the A>G base change at this position is not expected to have any structural effects.

-615U>C/G (m.2285U>C/G):

615U>C/G (m.2285U>C/G) (Yano et al., 2014). Position **615U (m.2285U)** forms a non-canonical base pair with 610C (m.2280C) within the loop capping H46, located at the back of the subunit and far from the tunnel and other functional sites. The structure of the loop is recognized by several mito-ribosomal proteins. Hydrogen bonds between MRPL43/mL43 and the RNA backbone at positions 612C (m.2282C), 614C (m.2284C), and **615U (m.2285U)**. A contact from MRPL44/mL44 to the base of 613C (m.2283C) is also modeled in the structure. Additional contact from proteins to the RNA backbone are visible involving MRPL57 and position 611A (m.2281A) and MRPL4/uL4 and MRPL51 and position 609U (m.2279U). MRPL15/uL15 is also very close but it does not establish direct contacts with the loop. All of this suggests that the structure of this loop is important for the recognition of these proteins

during assembly. In agreement with this, it has been shown that missense mutations in MRPL44 seriously affect the assembly of the large ribosomal subunit and the stability of 16S rRNA, resulting in the lack of complex IV (Wang et al., 2021, Carroll et al., 2013, Distelmaier et al., 2015). This protein:RNA arrangement is conserved in the mammalian mito-ribosome but completely absent in its bacterial counterpart. In *S. scrofa* (het. Equiv. 617C in 5AJ4 or 619C in the newer structures, 7NSH) the U●C base pair has been replaced by a C●U with the same geometry (density supported in 7NSH, 3.20-Å resolution). Taking all this evidence together, a picture emerges in which the loop atop H46 functions as a hub for the binding of a number of mito-ribosomal proteins during the assembly of the subunit. In this scenario, the **615U>C/G (m.2285U>C/G)** variants could introduce enough distortion in the structure of the loop to cause assembly defects.

-964U>C (m.2634U>C):

The rare variant **964U>C (m.2634U>C)** was found in a patient with non-syndromic hearing loss
(Yano et al., 2014). Position **964U (m.2634U)** is located to the RNA stretch linking H67 and H68 where it positions its base away from any potential hydrogen-bonding interaction. A U is also found at the heterologous equivalent position in the *S. scrofa* mito-ribosome, the human and plasmodium falciparum 80S, and in the bacterial ribosome. In some of these structures, the base is within hydrogen bonding distance to the backbone of H68. However, the equivalent distances in the human mito-ribosome are too long for such an interaction.

-1543A>G (m.3213A>G):

The **1543A>G (m.3213A>G)** variant was identified by Guaran et al. together with A3348G, G3591A, A3714G, G7642A, and G7805A (Guaran et al., 2013). Position **1543A (m.3213A)** base pairs with 1557U (m.3227U) in H100, adjacent to the 3'end of 16S mt-rRNA. Position 1540C (m.3210C) establishes a single hydrogen bond with 1557U (m.3227U), thus creating a triple base interaction. Numerous proteins contact H100. Several MRPL3/uL3m residues contact the RNA backbone of H100 at positions (m.3228U). Additionally, MRPL3/uL3m Arg 156 contacts the Hoogsteen face of the 1542C:1550G (m.3212C:3230G) base pair. MRPL32/bL32 contacts the ribose of 1542C (m.3212C) and establishes additional contacts to MRPL3/uL3m. MRPL17/uL17 also establishes contacts to the RNA backbone at position 1544C (m.3214C). Protein MRPL39/mL39 establishes contacts to all three mito-ribosomal proteins that contact H100, namely MRPL3/uL3m, MRPL17/uL17, and MRPL32/bL32, and to the mito-ribosomal tunnel protein MRPL22/ul22, further away. Mutations in MRPL3/uL3m have been identified in association with cardiomyopathy, neonatal lactic acidosis, sensorineural hearing loss, cirrhosis, and interstitial nephritis(Bursle et al., 2017, Galmiche et al., 2011). In addition, proper maturation of MRPL32/bL32 has been shown to be crucial for mitochondrial translation in yeast and in mammals (Nolden et al., 2005). Interactions are well supported by density. All this evidence indicates an important structural role for H100 as a hub for the proper folding of the mito-ribosomal LSU. While the **1543A>G (m.3213A>G)** variant would replace an A:U Watson-Crick base pair with a disfavored G•U wobble at the beginning of a helical segment (Ananth et al., 2013). Hence, it is possible that the substitution could slightly alter the structure of the region, thus affecting the assembly of the subunit. In this light, the potential pathogenicity of the **1543A>G (m.3213A>G)** variant cannot be ruled out.

Unclear variants

-220C>U (m.867C>U):

The **220C>U (m.867C>U)** variant was found in a patient with sensorineural hearing loss (Guaran et al., 2013). In the secondary structure of 12S mt-rRNA, position **220C (m.867G)** lies in the single-stranded stretch connecting helices h4 and h18. The O2 of **220C (m.867G)** has been modeled sharing a hydrogen bond with the amino group of 223C (m.870C), also within the same rRNA stretch (not shown). However, the quality of the density in this region cannot be used to confirm the accuracy of this interaction. This region is recognized by the protein MRPS18B/bS18b. No conclusions can be drawn from the data.

-380A>G (m.1027A>G):

The **380A>G (m.1027A>G)** variant was identified by Lu et al. (Lu et al., 2010). Position **380A (m.1027A)** is located in the GNRA tetraloop capping h23a. A bifurcated sheared base pair between 377G (m.1024G) and **380A (m.1027A)** closes the GNRA tetraloop (Correll Carl C. et al., 1998). While the structure of this base pair should not be disrupted by the A>G substitution, the C2 of **380A (m.1027A)** lies only 3.7 Å away from the RNA backbone at position 406A (m.1053A), suggesting that the presence of a bulky 2-amino group, as a result of the A>G base change, could be disruptive. Since the GNRA tetraloop docks into the minor groove of h20 via a conserved double A-minor interaction mediated by positions 378-9A (m.1025-6A) (Noller., 2005), a small disruption of the local structure could have ampler effects.

Supplementary Figures

All sites of variation discussed in the main text are shown here in the context of the secondary structure of 12S mt-rRNA, as well as in the higher-order structure context of the mito-ribosome. All Suppl. Figures were created with Chimera X (Pettersen et al., 2021). Most of the structural data was obtained with the 2.2-Å cryo-EM human mito-ribosomal structure (RCSB Protein Data Bank ID: 8ANY)(Itoh et al., 2021, Itoh et al., 2022). All other structures are mentioned in the legends. At least one panel is used to show the agreement between the structural model and the experimental electron density. Contour levels were adjusted to display the shape of RNA residues. In cases in which the chosen contour level did not cover certain features of interest in the model, a second panel at a lower contour level might be included in the Suppl. Figure.

Supplementary Figure S1

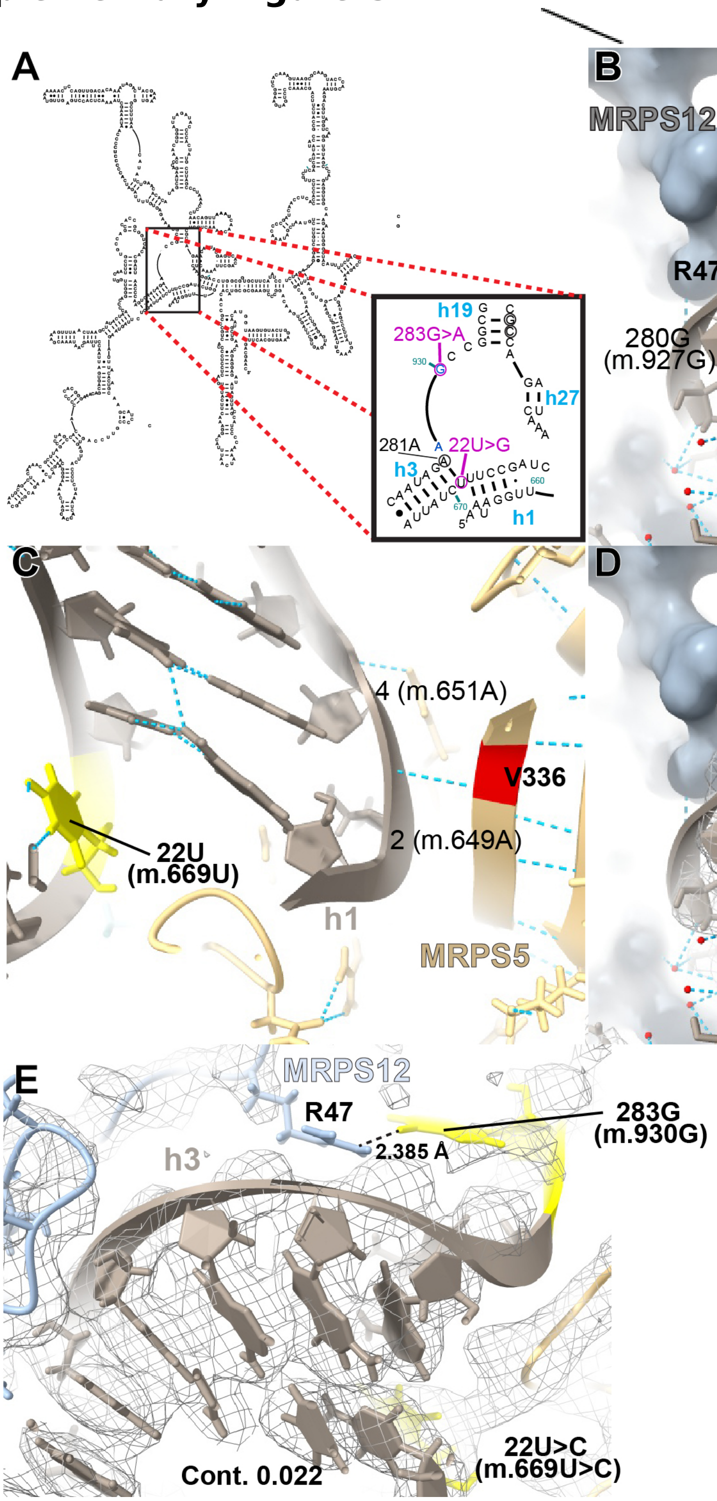

Supplementary Figure S1. Positions 22U (m.669U) and 283G (m.930G) in the human mito-ribosome. A. Localization of the variant-containing region in the secondary structure map of 12S mt-rRNA. Sites of variation are labeled in magenta. Additional sites are labeled in black. Helix numbers are shown in light blue. Symbols: "-", canonical base pair; "•", wobble base pair; "●", non-canonical base pair, thick, black line, physical connection and continuity between adjacent bases that are drawn distantly in the secondary-structure map. **B** and **C.** Annotated views of the region containing the **22U>C (m.669U>C)** and **283G>A (m.930G>A)** variants (yellow and labeled in bold, black font). Other rRNA residues are labeled in regular font. 12S mt-rRNA is shown in grey with helix numbers indicated. MRPS12/uS12m is shown as a grey surface. Other components of the mito-ribosome are labeled and color coded to their molecular model. The positions of R47 of MRPS12/uS12m and V336 of MRPS5/uS5m are indicated in **B-D**. Hydrogen bonds are indicated by blue, broken lines. Water

molecules are shown as red spheres. **D-E.** Chimera X-rendered electron density (black mesh) at a contour level of 0.032 (**D**) and 0.022 (**E**) (Pettersen et al., 2021). MRPS12/uS12m is shown in light blue in **E**. The molecular model and electron density map from the 2.2-Å cryo-EM human mito ribosomal structure (RCSB ID: 8ANY) (Itoh et al., 2021, Itoh et al., 2022) were used to create panels **B-E.**

Supplementary Figure S2

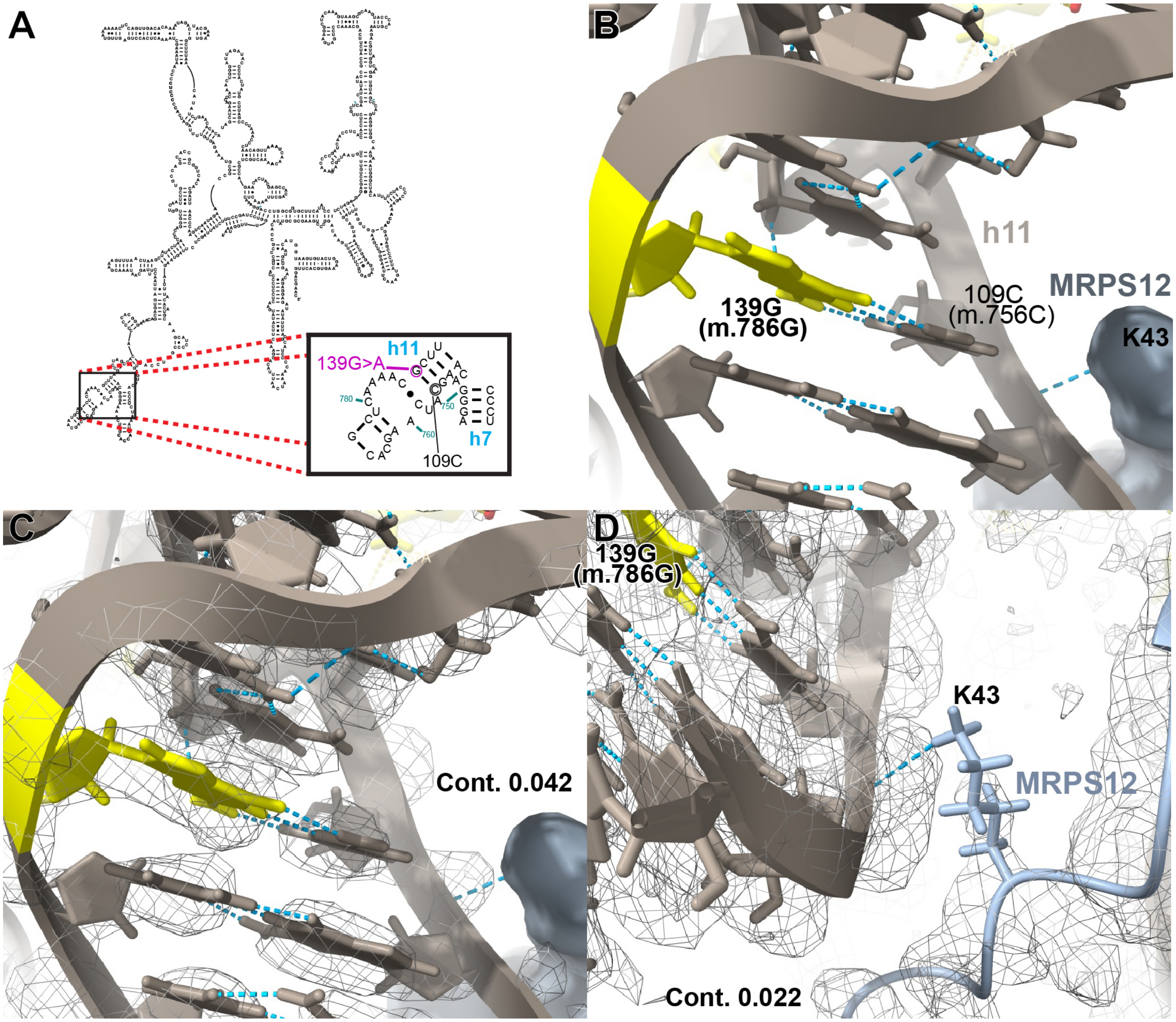

Supplementary Figure S2. Positions 139G (m.786G) in the human mito-ribosome. A. Localization of the variant-containing region in the secondary structure map of 12S mt-rRNA. Sites of variation are labeled in magenta. Additional sites are labeled in black. Helix numbers are shown in light blue. Symbols: "-", canonical base pair; "•", wobble base pair; "●", non-canonical base pair, thick, black line, physical connection and continuity between adjacent bases that are drawn distantly in the secondary-structure map. **B.** Annotated view of the region containing the **139G>A (m.786G>A)** variant (yellow and labeled in bold, black font). Other rRNA residues labeled in regular font. 12S mt-rRNA is shown in grey with helix numbers indicated. Other components of the mito-ribosome are labeled, and color coded to their molecular model. The position of K43 of MRPS12/uS12m is indicated in **B** and **D**. Hydrogen bonds are indicated by blue, broken lines. MRPS12/uS12m is shown as a grey surface. **C** and **D.** Chimera X-rendered electron density (black mesh) at contour level of 0.042 (**C**) and 0.022 (**D**) (Pettersen et al., 2021). MRPS12/uS12m is shown in light blue in **D**. The molecular model and electron density map from the 2.2-Å cryo-EM human mito-ribosomal structure (RCSB ID: 8ANY) (Itoh et al., 2021, Itoh et al., 2022) were used to create panels **B-D**.

Supplementary Figure S3

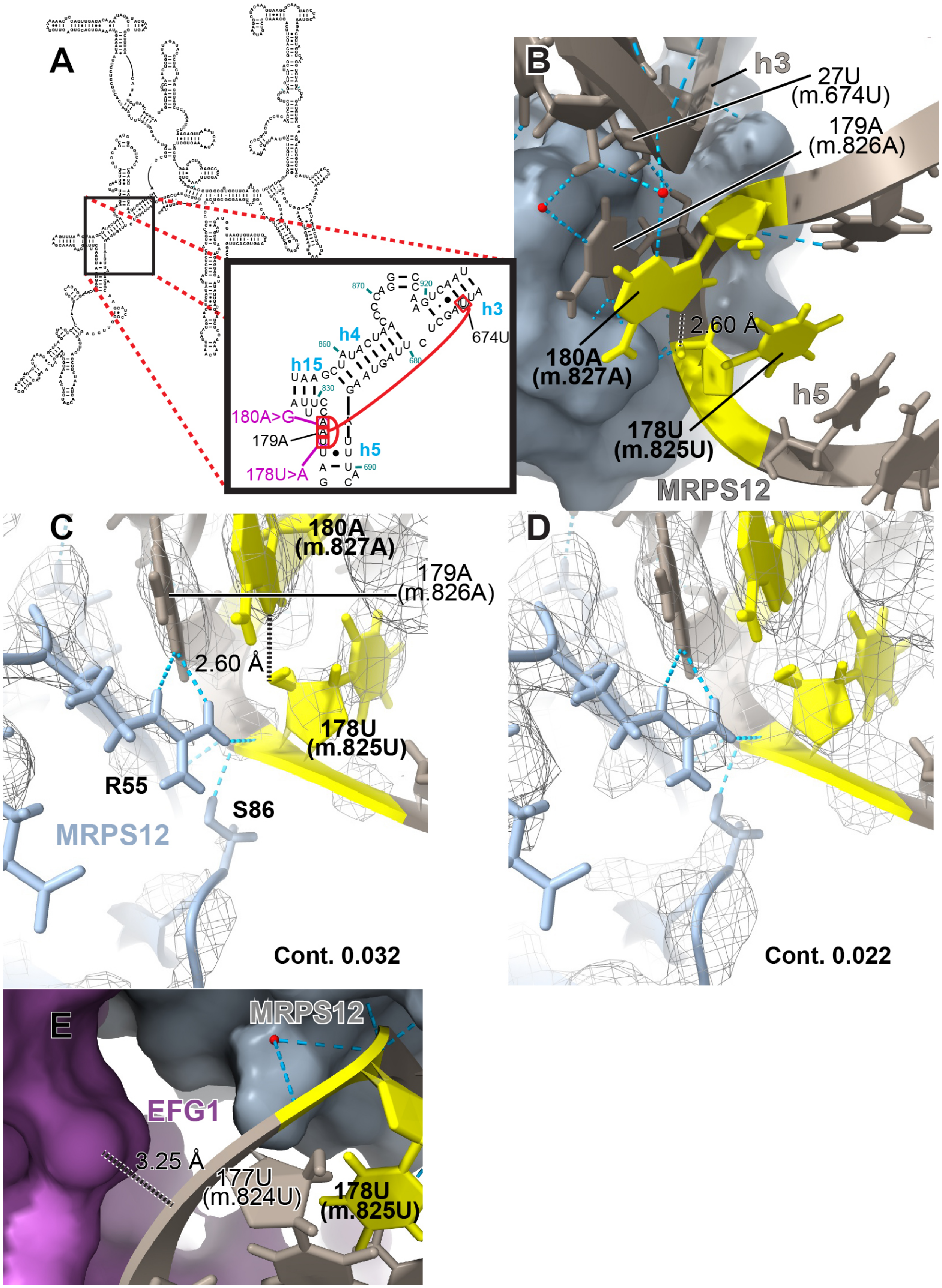

Supplementary Figure S3. Positions 178U (m.825U) and 180A (m.827A) in the human mito-ribosome. A. Localization of the variant-containing region in the secondary structure map of 12S mt-rRNA. Sites of variation are labeled in magenta. Additional sites are labeled in black. Helix numbers are shown in light blue. Symbols: "-" canonical base pair; "•", wobble base pair; "●", non-canonical base pair; thick, black line, physical connection and continuity between adjacent bases that are drawn distantly in the secondary-structure map; red squares connected by red, thick lines, tertiary interactions. **B.** Annotated view of the region containing the **178U>A (m.825U>A)** and **180A>G (m.827A>G)** variants (yellow and labeled in bold, black font). Other rRNA residues labeled in regular font. 12S mt-rRNA is shown in grey with helix numbers indicated. Other components of the mito-ribosome are labeled, and color coded to their molecular model. Hydrogen bonds are indicated by blue, broken lines. MRPS12/uS12m is shown as a grey surface. **C** and **D**. Chimera X-rendered electron density (black mesh) at contour level of 0.032 (**C**) and 0.022 (**D**), the latter used to demonstrate the existence of clear electron density around MRPS12/uS12m residues (Pettersen et al., 2021). MRPS12/uS12m is shown in light blue in **C** and **D**. The positions of R55 and S86 of MRPS12/uS12m are indicated in **C**. **E.** Position of EFG1 (magenta surface), relative to 12S rRNA. Distances are denoted with black, broken lines. The molecular model and electron density map from the 2.2-Å cryo-EM human mito-ribosomal structure (Itoh et al., 2021, Itoh et al., 2022) were used to create panels **B-D**. The EFG1 structure was obtained from RCSB: 6VMI (Koripella et al., 2020) and superimposed onto the 2.2-Å cryo-EM human mito-ribosomal structure (RCSB ID: 8ANY) (Itoh et al., 2021, Itoh et al., 2022) with the ChimeraX Matchmaker utility, using MRPS12 as the reference chain (Pettersen et al., 2021).

Supplementary Figure S4

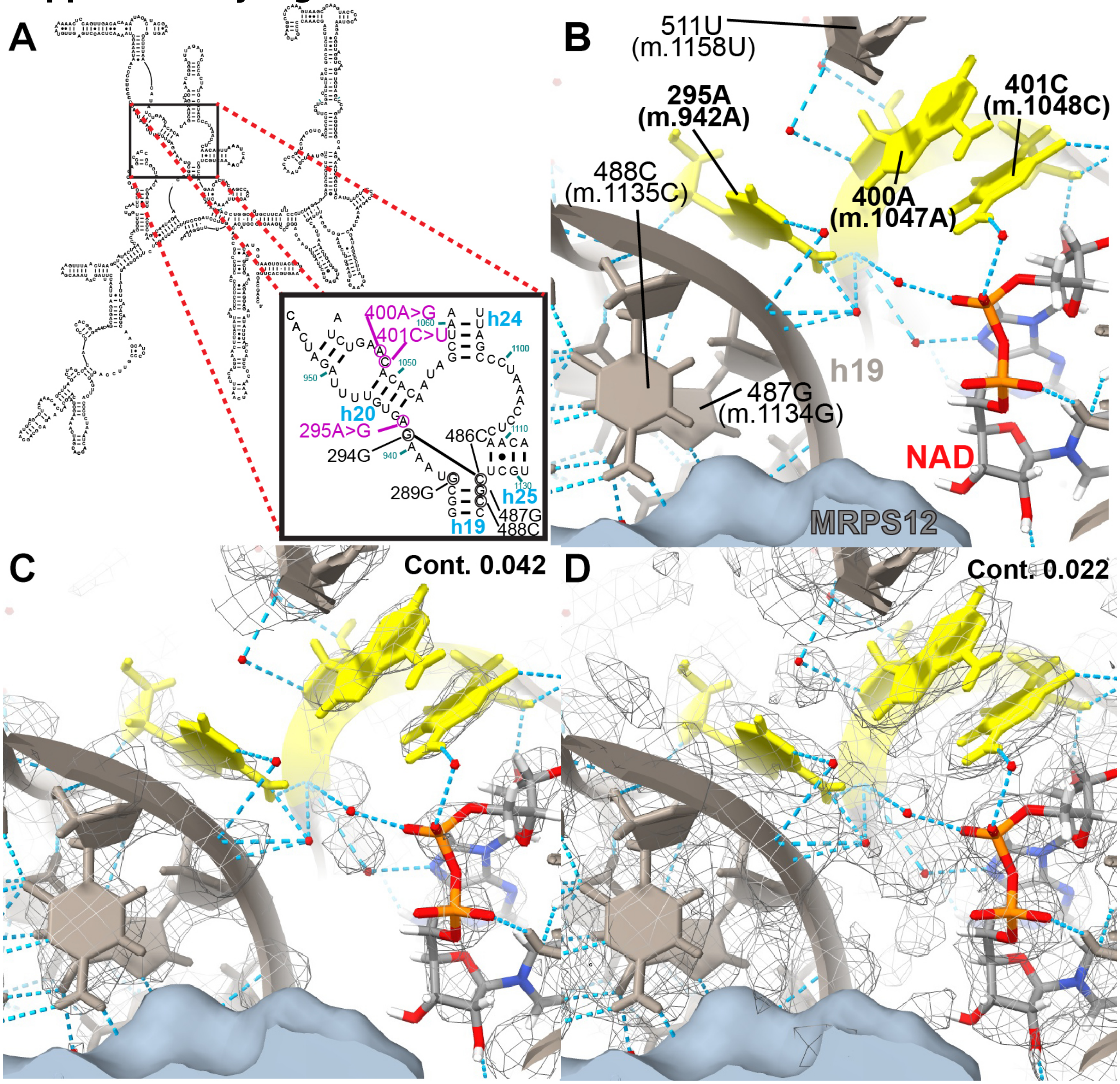

Supplementary Figure S4. Position 295A (m.942A), 400A (m.1047A), and **401C (m.1048C) in the human mito-ribosome. A.** Localization of the variant-containing region in the secondary structure map of 12S mt-rRNA. Sites of variation are labeled in magenta. Additional sites are labeled in black. Helix numbers are shown in light blue. Symbols: "-", canonical base pair; "•", wobble base pair; "●", non-canonical base pair; thick, black line, physical connection and continuity between adjacent bases that are drawn distantly in the secondary-structure map; red squares connected by red, thick lines, tertiary interactions. **B.** Annotated view of the region containing the **295A>G (m.942A>G)**, **400A>G (m.1047A>G)**, and **401C>U (m. 1048C>U)** variants (yellow and labeled in bold, black font). Other rRNA residues labeled in regular font. 12S mt-rRNA is shown in grey with helix numbers indicated. Other components of the mito-ribosome are labeled and color coded to their molecular model. The position of NAD (heteroatom coloring) is indicated. Hydrogen bonds are indicated by blue, broken lines. Distances are denoted with white, broken lines. MRPS12/uS12m is shown as a grey surface. Water molecules are shown as red spheres. **C** and **D.** Chimera X-rendered electron density (black mesh) at contour level of 0.042 (**C**) and 0.022 (**D**), the latter used to demonstrate the existence of clear electron density around the water molecules mentioned in the text (Pettersen et al., 2021). The molecular model and electron density map from the 2.2-Å cryo-EM human mito-ribosomal structure (RCSB ID: 8ANY) (Itoh et al., 2021, Itoh et al., 2022) were used to create panels **B-D**.

Supplementary Figure S5

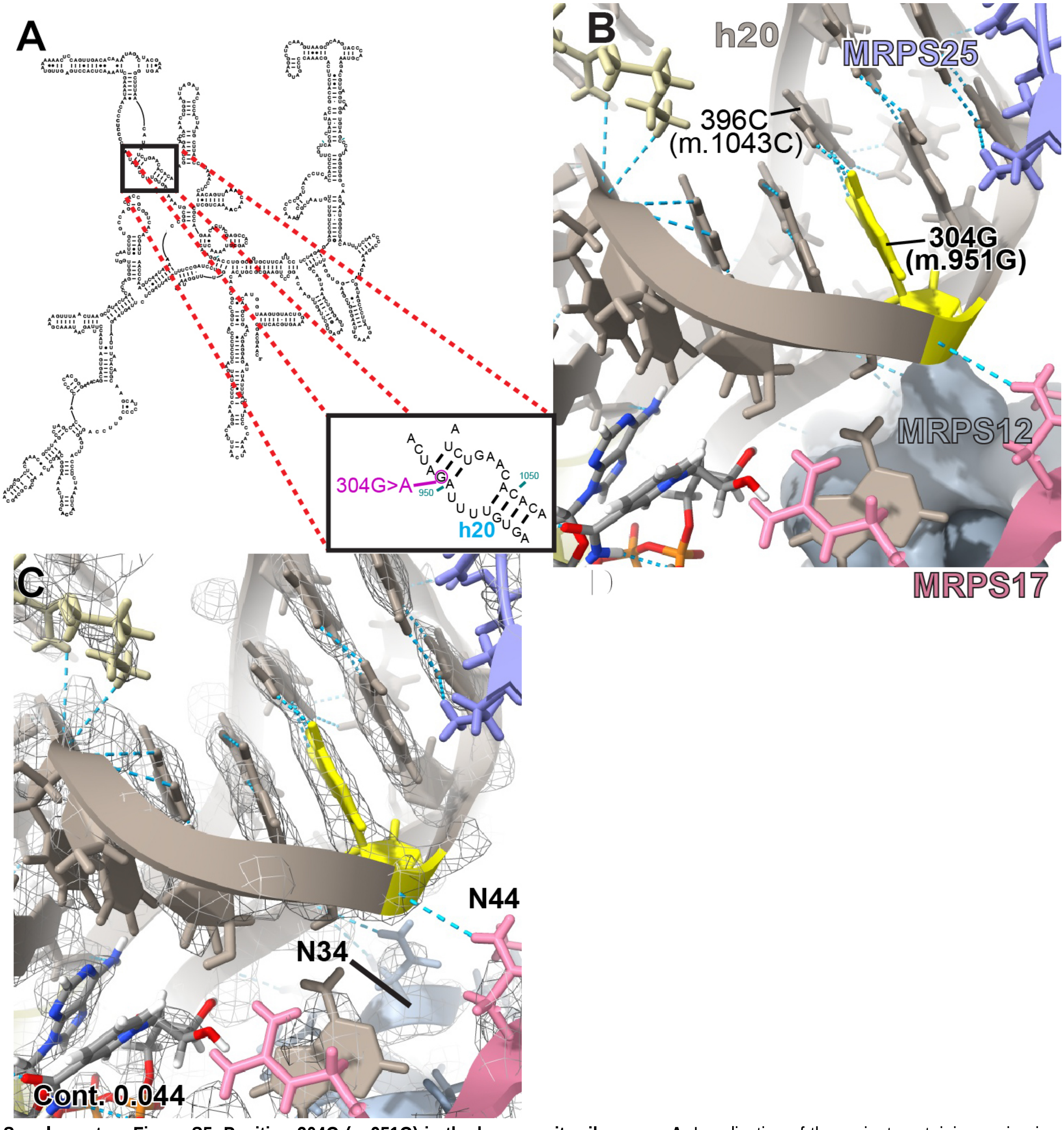

Supplementary Figure S5. Position 304G (m.951G) in the human mito-ribosome. A. Localization of the variant-containing region in the secondary structure map of 12S mt-rRNA. Sites of variation are labeled in magenta. Additional sites are labeled in black. Helix numbers are shown in light blue. Symbols: "-", canonical base pair; "•", wobble base pair; "●", non-canonical base pair; thick, black line, physical connection and continuity between adjacent bases that are drawn distantly in the secondary-structure map; red squares connected by red, thick lines, tertiary interactions. **B.** Annotated view of the region containing the **304G>A (m.951G>A)** variant (yellow and labeled in bold, black font). Other rRNA residues are labeled in regular font. 12S mt-rRNA is shown in grey with helix numbers indicated. Other components of the mito-ribosome are labeled, and color coded to their molecular model. Hydrogen bonds are indicated by blue, broken lines. Distances are denoted with white, broken lines. MRPS12/uS12m is shown as a grey surface. **C.** Chimera X-rendered electron density (black mesh) at contour level of 0.044 (Pettersen et al., 2021). The positions of N34 of MRPS12/uS12m and

N44 of MRPS17/uS17 are indicated. The molecular model and electron density map from the 2.2-Å cryo-EM human mito-ribosomal structure (RCSB ID: 8ANY) (Itoh et al., 2021, Itoh et al., 2022) were used to create panels **B** and **C**.

Supplementary Figure S6

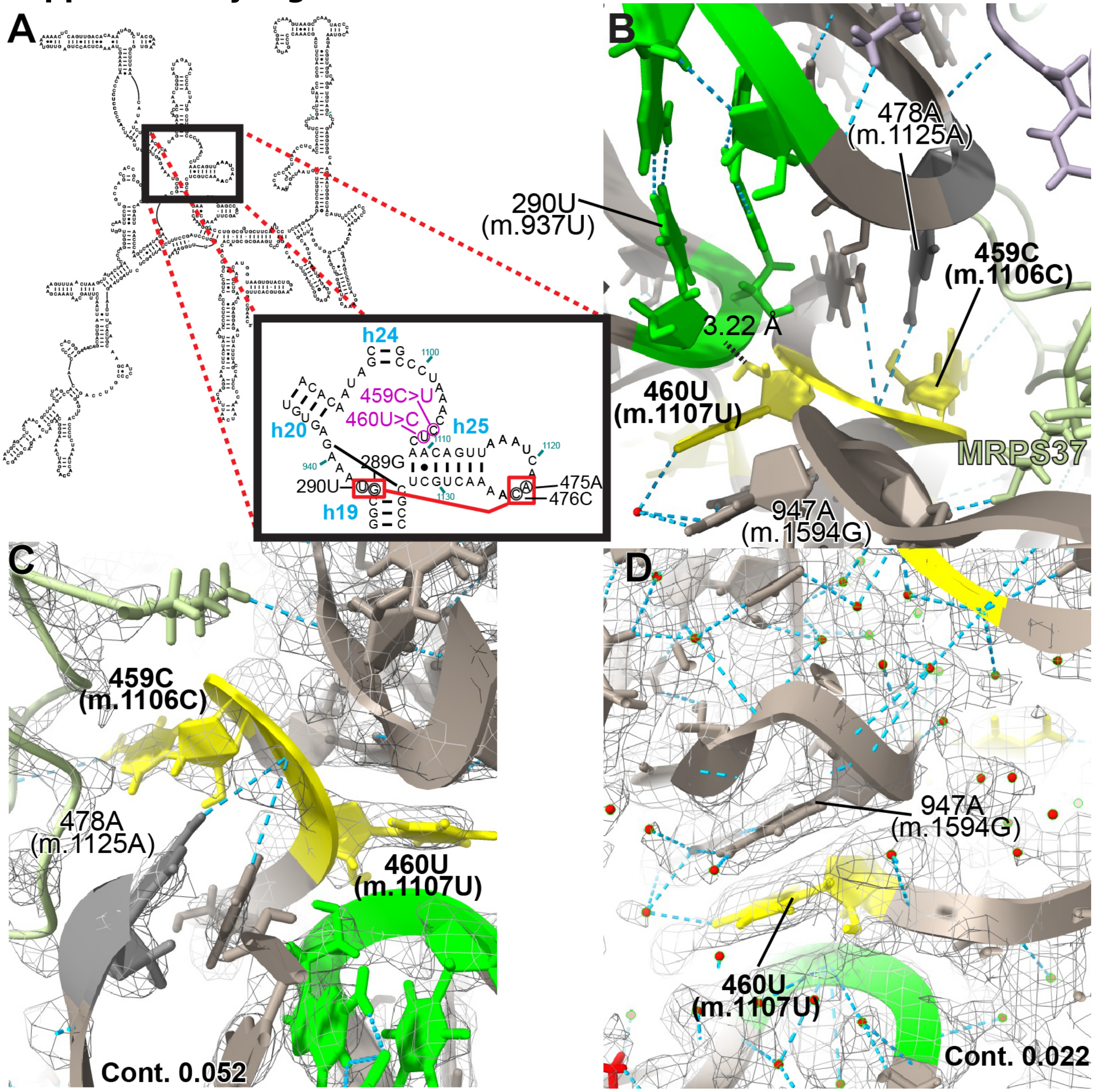

Supplementary Figure S6. Positions 459C (m.1106C) and 460U (m.1107U) in the human mito-ribosome. A. Localization of the variant-containing region in the secondary structure map of 12S mt-rRNA. Sites of variation are labeled in magenta. Additional sites are labeled in black. Helix numbers are shown in light blue. Symbols: "-", canonical base pair; "•", wobble base pair; "●", non-canonical base pair; thick, black line, physical connection and continuity between adjacent bases that are drawn distantly in the secondary-structure map; red squares connected by red, thick lines, tertiary interactions (central domain pseudoknot). **B.** Annotated view of the region containing the **459C>U (m.1106C>U)** and **460U>C (m.1107U>C)** variants (yellow and labeled in bold, black font). Other rRNA residues are labeled in regular font. 12S mt-rRNA is shown in grey with helix numbers indicated. Other components of the mito-ribosome are labeled and color coded to their molecular model. Distances are denoted with black, broken lines. Hydrogen bonds are indicated by blue, broken lines. Distances are denoted with white, broken lines. Water molecules are shown as red spheres. **C** and **D.** Chimera X-rendered electron density (black mesh) at contour level of 0.052 (**C**) and 0.022 (**D**) (Pettersen et al., 2021). The molecular model and electron density map

from the 2.2-Å cryo-EM human mito-ribosomal structure (RCSB ID: 8ANY) (Itoh et al., 2021, Itoh et al., 2022) were used to create panels **B-D**.

Supplementary Figure S7

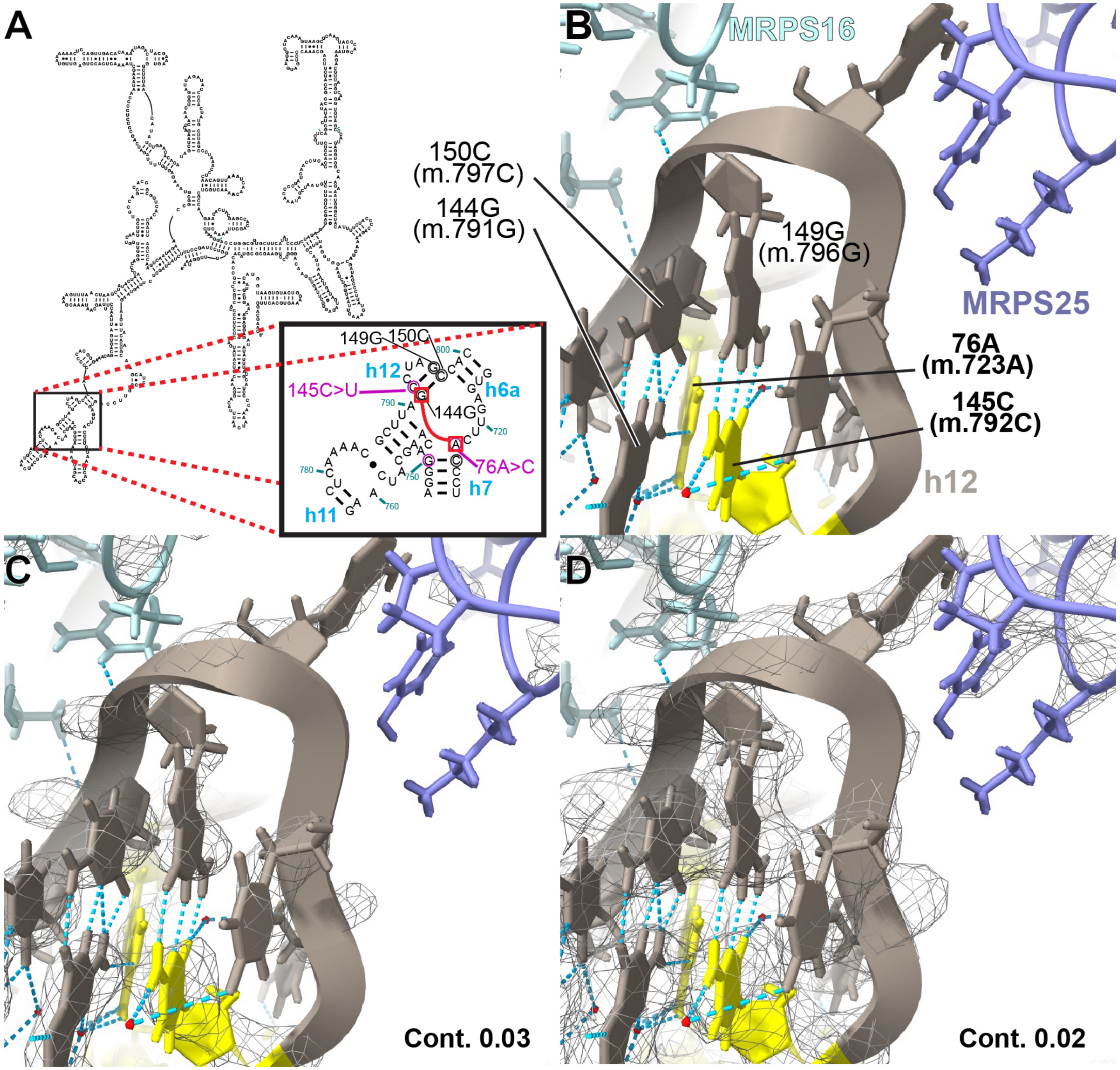

Supplementary Figure S7. Positions 76A (m.723A) and 145C (m.792C) in the human mito-ribosome. A. Localization of the variant-containing region in the secondary structure map of 12S mt-rRNA. Sites of variation are labeled in magenta. Additional sites are labeled in black. Helix numbers are shown in light blue. Symbols: "-", canonical base pair; "•", wobble base pair; "●", non-canonical base pair; thick, black line, physical connection and continuity between adjacent bases that are drawn distantly in the secondary-structure map; red squares connected by red, thick lines, tertiary interactions. **B.** Annotated view of the region containing the **76A>C (m.723A>C)** and **145C>U (m.792C>U)** variants (yellow and labeled in bold, black font). Other rRNA residues are labeled in regular font. 12S mt-rRNA is shown in grey with helix numbers indicated. Other components of the mito-ribosome are labeled, and color coded to their molecular model. Hydrogen bonds are indicated by blue, broken lines. Distances are denoted with white, broken lines. Water molecules are shown as red spheres. **C** and **D.** Chimera X-rendered electron density (black mesh) at contour level of 0.03 (**C**) and 0.02 (**D**), the latter used to better resolve the electron density around MRPS25/mS25 (Pettersen et al., 2021). The molecular model and electron density map from the 2.2-Å cryo-EM human mito-ribosomal structure (RCSB ID: 8ANY) (Itoh et al., 2021, Itoh et al., 2022) were used to create panels **B-D**.

Supplementary Figure S8

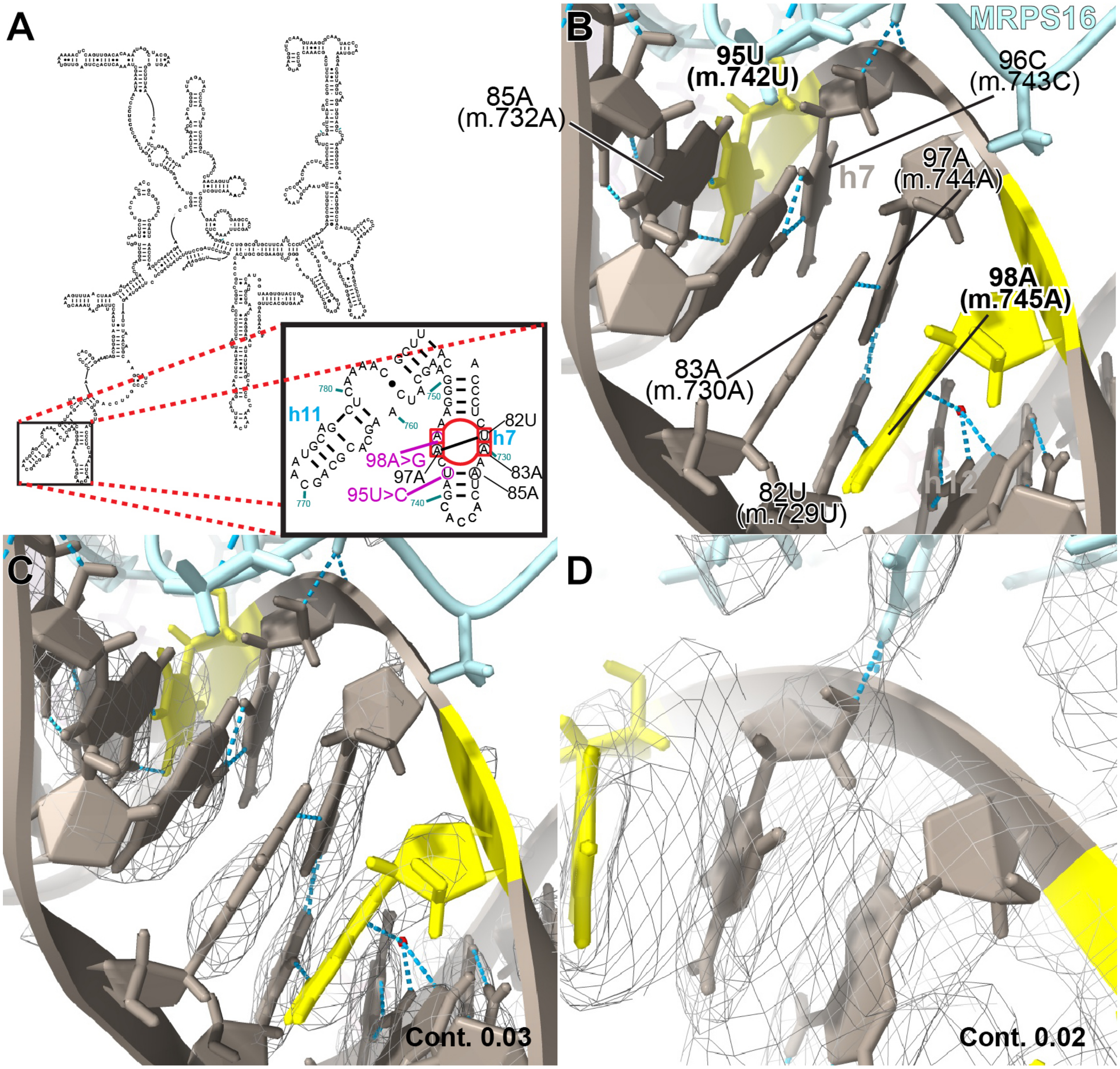

Supplementary Figure S8. Positions 95U (m.742U) and 98A (m.745A) in the human mito-ribosome. A. Localization of the variant-containing region in the secondary structure map of 12S mt-rRNA. Sites of variation are labeled in magenta. Additional sites are labeled in black. Helix numbers are shown in light blue. Symbols: "-", canonical base pair; "•", wobble base pair; "●", non-canonical base pair; thick, black line, physical connection and continuity between adjacent bases that are drawn distantly in the secondary-structure map; red squares connected by red, thick lines, tertiary interactions. **B.** Annotated view of the region containing the **95U>C (m.742U>C)** and **98A>G (m.745A>G)** variants (yellow and labeled in bold, black font). Other rRNA residues labeled in regular font. 12S mt-rRNA is shown in grey with helix numbers indicated. Other components of the mito-ribosome are labeled, and color coded to their molecular model. Hydrogen bonds are indicated by blue, broken lines. Distances are denoted with white, broken lines. Water molecules are shown as red spheres. **C** and **D.** Chimera X-rendered electron density (black mesh) at contour level of 0.03 (**C**) and 0.02 (**D**), the latter panel focused on the interaction between h7 and MRPS16/bS16 (Pettersen et al., 2021). The molecular model and electron density map from the 2.2-Å cryo-EM human mito-ribosomal structure (RCSB ID: 8ANY) (Itoh et al., 2021, Itoh et al., 2022) were used to create panels **B-D**.

Supplementary Figure S9

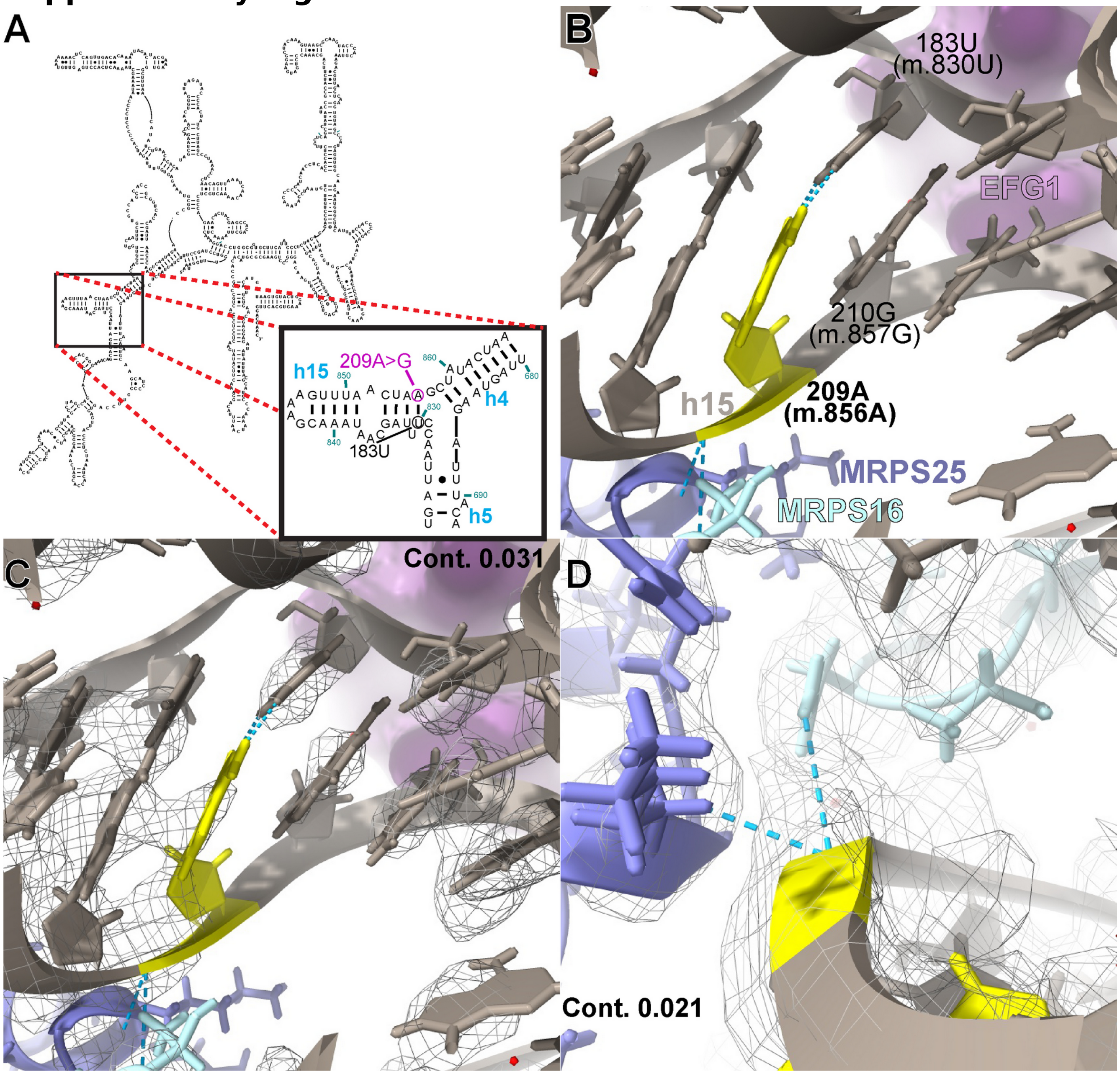

Supplementary Figure S9. Position 209A (m.856A) in the human mito-ribosome. A. Localization of the variant-containing region in the secondary structure map of 12S mt-rRNA. Sites of variation are labeled in magenta. Additional sites are labeled in black. Helix numbers are shown in light blue. Symbols: "-", canonical base pair; "•", wobble base pair; "●", non-canonical base pair; thick, black line, physical connection and continuity between adjacent bases that are drawn distantly in the secondary-structure map; red squares connected by red, thick lines, tertiary interactions. **B.** Annotated view of the region containing the **209A>G (m.856A>G)** variant (yellow and labeled in bold, black font). Other rRNA residues labeled in regular font. 12S mt-rRNA is shown in grey with helix numbers indicated. Other components of the mito-ribosome are labeled, and color coded to their molecular model. Hydrogen bonds are indicated by blue, broken lines. Distances are denoted with white, broken lines. Water molecules are shown as red spheres. **C** and **D.** Chimera X-rendered electron density (black mesh) at contour level of 0.031 (**C**) and 0.021 (**D**) (Pettersen et al., 2021). The molecular model and electron density map from the 2.2-Å cryo-EM human mito-ribosomal structure (Itoh et al., 2021, Itoh et al., 2022) were used to create panels **B-D**. EFG1 is shown as a magenta surface. The EFG1 structure was obtained from RCSB: 6VMI (Koripella et al., 2020) and superimposed onto the 2.2-Å cryo-EM human mito-ribosomal structure (RCSB ID: 8ANY) (Itoh et al., 2021, Itoh et al., 2022) with the ChimeraX Matchmaker utility, using MRPS12 as the reference chain (Pettersen et al., 2021).

Supplementary Figure S10

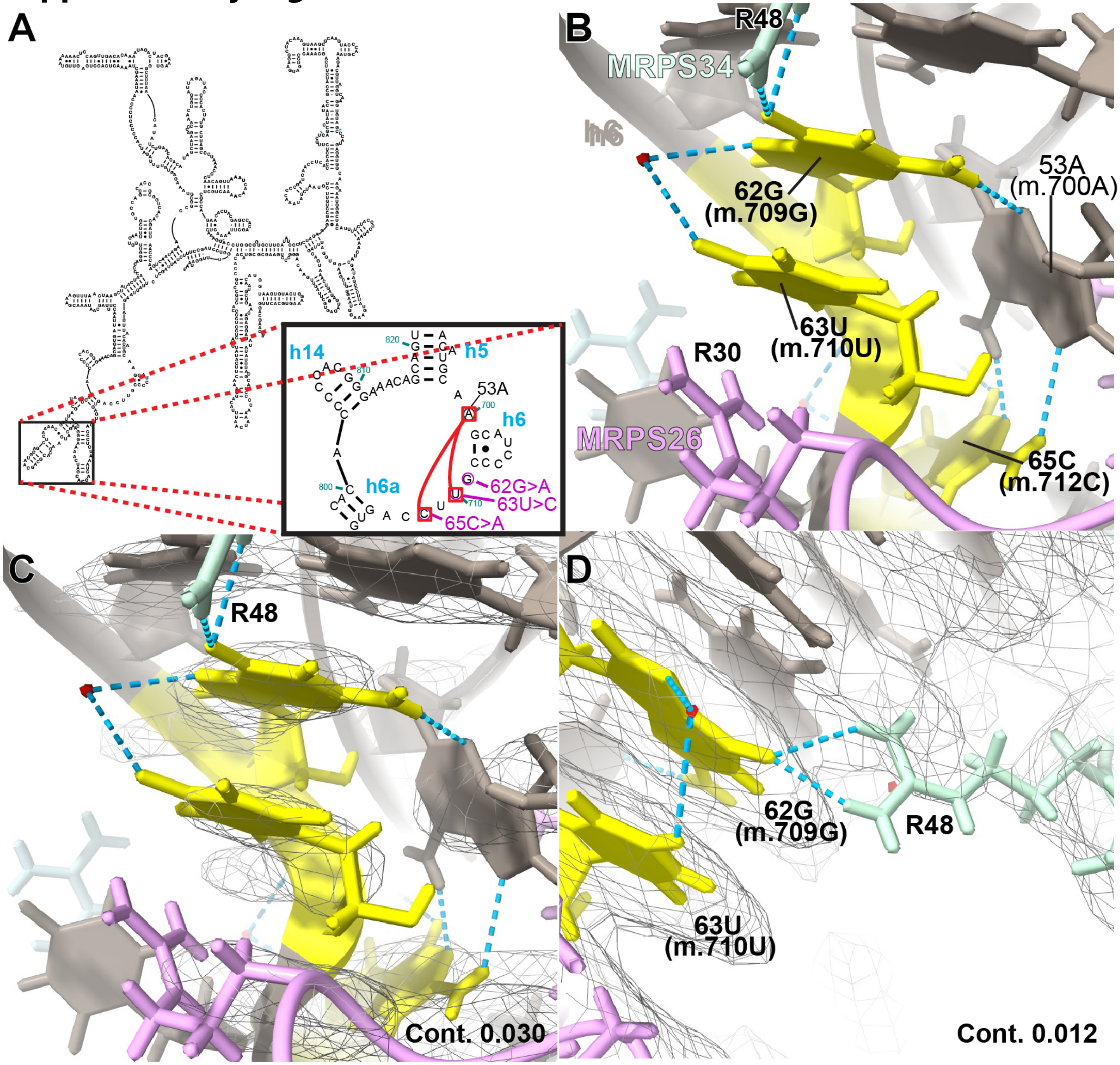

Supplementary Figure S10. Positions 62G (m.709G), 63U (m.710U) and 65C (m.712C) in the human mito-ribosome. A. Localization of the variant-containing region in the secondary structure map of 12S mt-rRNA. Sites of variation are labeled in magenta. Additional sites are labeled in black. Helix numbers are shown in light blue. Symbols: "-", canonical base pair; "•", wobble base pair; "●", non-canonical base pair; thick, black line, physical connection and continuity between adjacent bases that are drawn distantly in the secondary-structure map; red squares connected by red, thick lines, tertiary interactions. **B.** Annotated view of the region containing the **62G>A (m.709G>A), 63U>C (m.710U>C)** and **65C>A (m.712C>A)** variants (yellow and labeled in bold, black font). Other rRNA residues are labeled in regular font. 12S mt-rRNA is shown in grey with helix numbers indicated. Other components of the mito-ribosome are labeled, and color coded to their molecular model. The positions of R48 of MRPS34/mS34 and R30 of MRPS26/mS26 are indicated in panels **B-D**. Hydrogen bonds are indicated by blue, broken lines. Distances are denoted with white, broken lines. Water molecules are shown as red spheres. **C** and **D.** Chimera X-rendered electron density (black mesh) at contour level of 0.030 (**C**) and 0.012 (**D**), the latter panel focused on the interaction between **62G (m.709G)** and R48 of MRPS34/mS34 (Pettersen et al., 2021). The molecular model and electron density map from the 2.2-Å cryo-EM human mito-ribosomal structure (RCSB ID: 8ANY) (Itoh et al., 2021, Itoh et al., 2022) were used to create panels **B-D**.

Supplementary Figure S11

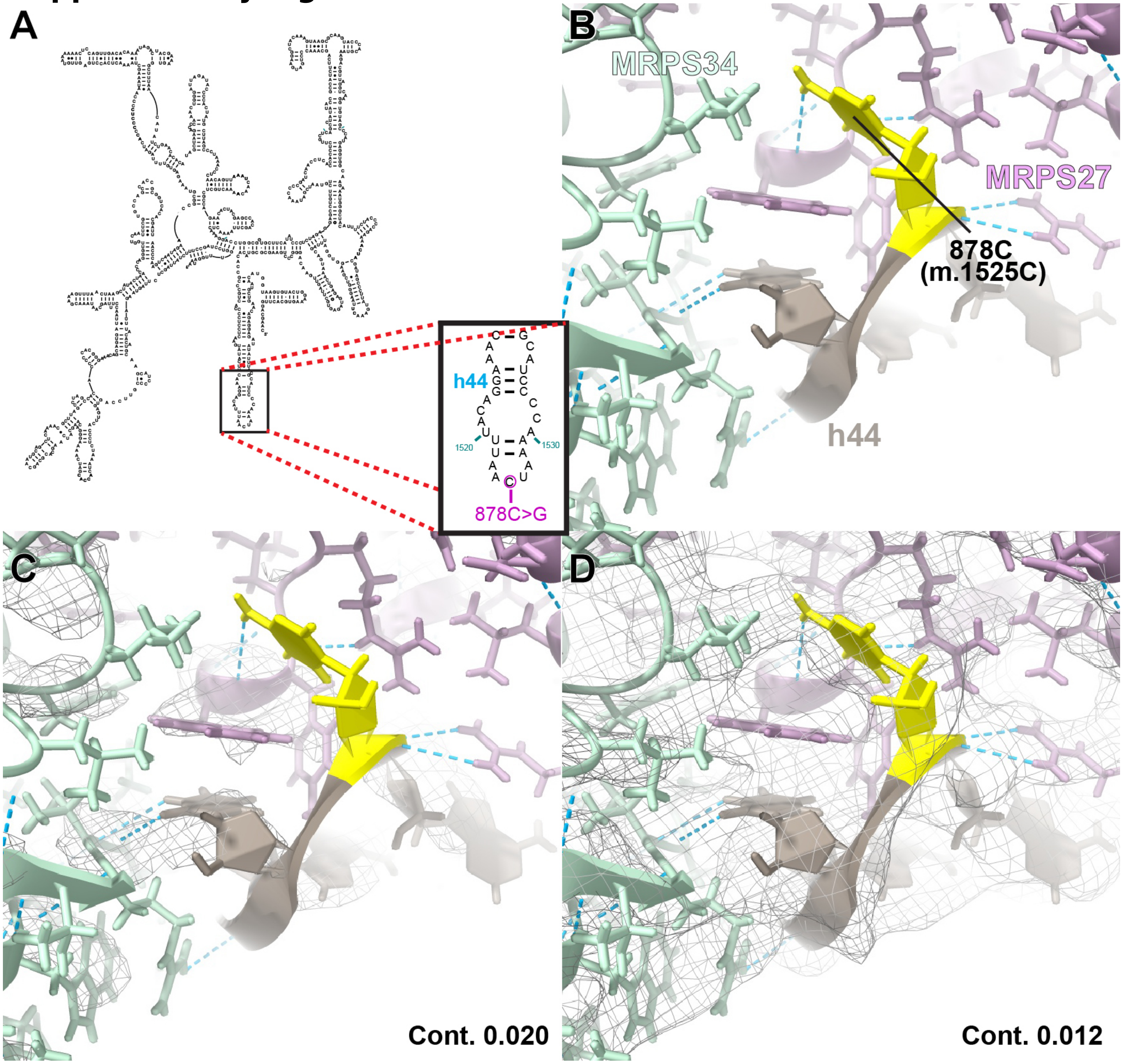

Supplementary Figure S11. Position 878C (m.1525C) in the human mito-ribosome. A. Localization of the variant-containing region in the secondary structure map of 12S mt-rRNA. Sites of variation are labeled in magenta. Additional sites are labeled in black. Helix numbers are shown in light blue. Symbols: "-", canonical base pair; "•", wobble base pair; "●", non-canonical base pair; thick, black line, physical connection and continuity between adjacent bases that are drawn distantly in the secondary-structure map; red squares connected by red, thick lines, tertiary interactions. **B.** Annotated view of the region containing the **878C>G (m.1525C>G)** variant (yellow and labeled in bold, black font). Other rRNA residues labeled in regular font. 12S mt-rRNA is shown in grey with helix numbers indicated. Other components of the mito-ribosome are labeled, and color coded to their molecular model. Hydrogen bonds are indicated by blue, broken lines. Distances are denoted with white, broken lines. Water molecules are shown as red spheres. **C** and **D.** Chimera X-rendered electron density (black mesh) at contour level of 0.020 (**C**) and 0.012 (**D**), the latter used to demonstrate the existence of clear electron density around the variant position (Pettersen et al., 2021). The molecular model and electron density map from the 2.2-Å cryo-EM human mito-ribosomal structure (RCSB ID: 8ANY) (Itoh et al., 2021) were used to create panels **B-D**.

Supplementary Figure S12

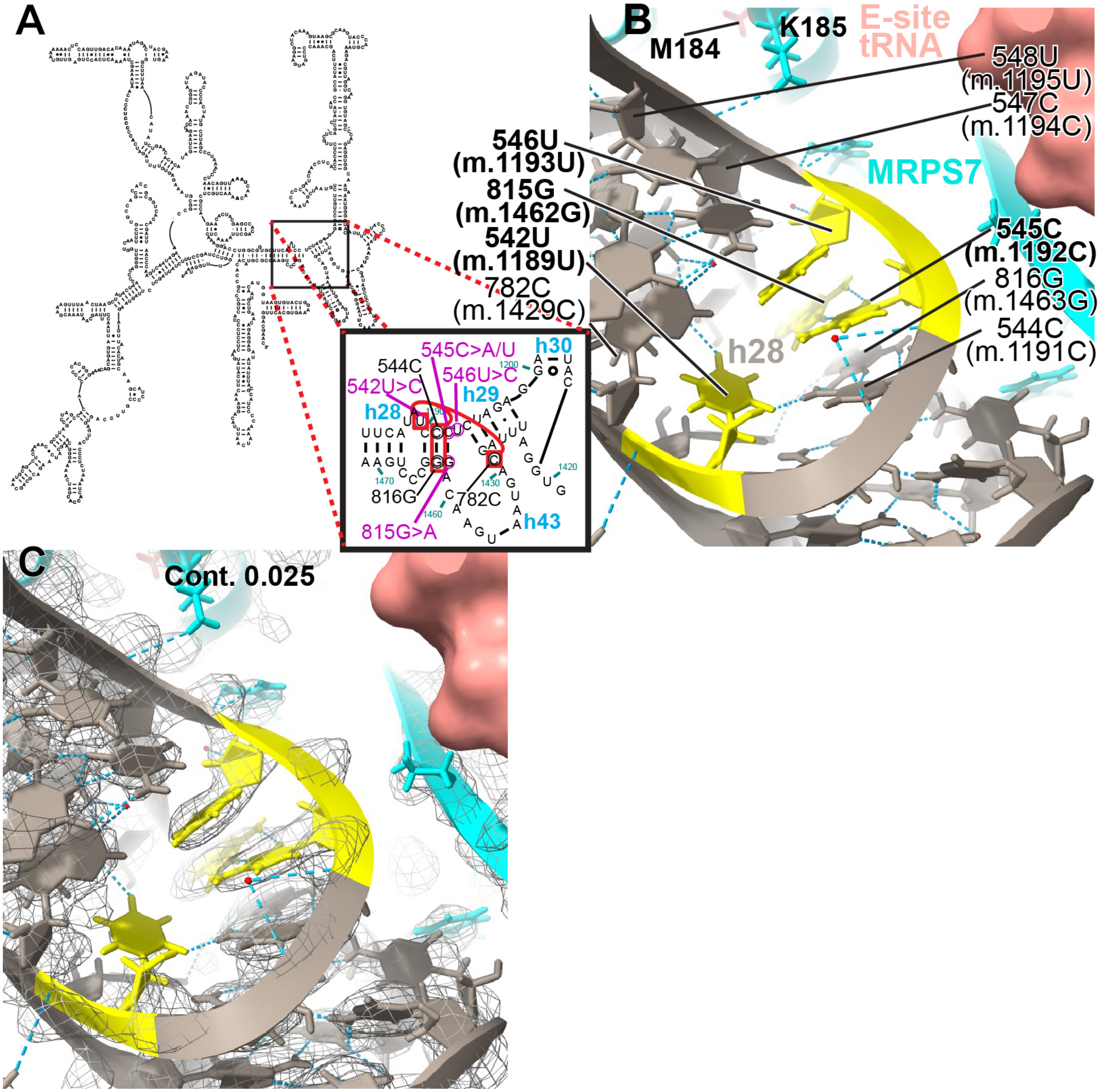

Supplementary Figure S12. Positions 542U (m.1189U), 545C (m.1192C), 546U (m.1193U), and 815G (m.1462G) in the human mito-ribosome. A. Localization of the variant-containing region in the secondary structure map of 12S mt-rRNA. Sites of variation are labeled in magenta. Additional sites are labeled in black. Helix numbers are shown in light blue. Symbols: "-", canonical base pair; "•", wobble base pair; "●", non-canonical base pair; thick, black line, physical connection and continuity between adjacent bases that are drawn distantly in the secondary-structure map; red squares connected by red, thick lines, tertiary interactions. **B.** Annotated view of the region containing the **542U>C (m.1189U>C)**, **545C>A/U (m.1192C>A/U)**, **546U>C (m.1193U>C)**, and **815G>A (m.1462G>A)** variants (yellow and labeled in bold, black font). Other rRNA residues are labeled in regular font. 12S mt-rRNA is shown in grey with helix numbers indicated. E-site tRNA is shown as a salmon surface. Other components of the mito-ribosome are labeled, and color coded to their molecular model. The positions of M184 and K185 of MRPS7/uS7m are indicated. Hydrogen bonds are indicated by blue, broken lines. Distances are denoted with white, broken lines. Water molecules are shown as red spheres. **C.** Chimera X-rendered electron density

(black mesh) at contour level of 0.025 (Pettersen et al., 2021). The molecular model and electron density map from the 2.2-Å cryo-EM human mito-ribosomal structure (RCSB ID: 8ANY) (Itoh et al., 2021, Itoh et al., 2022) were used to create panels **B**-**D**.

Supplementary Figure S13

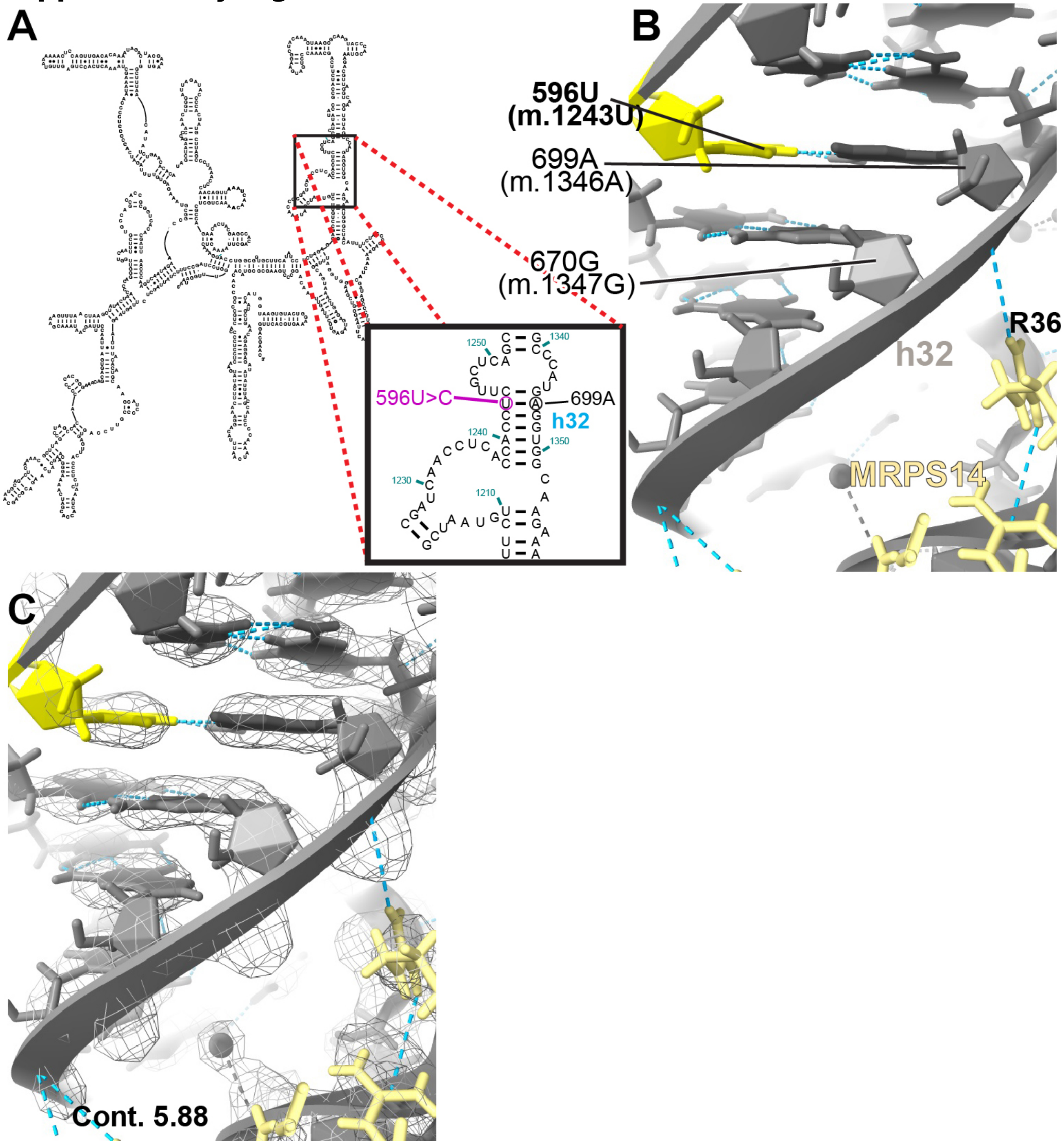

Supplementary Figure S13. Position 596U (m.1243U) in the human mito-ribosome. A. Localization of the variant-containing region in the secondary structure map of 12S mt-rRNA. Sites of variation are labeled in magenta. Additional sites are labeled in black. Helix numbers are shown in light blue. Symbols: "-", canonical base pair; "•", wobble base pair; "●", non-canonical base pair; thick, black line, physical connection and continuity between adjacent bases that are drawn distantly in the secondary-structure map; red squares connected by red, thick lines, tertiary interactions. **B.** Annotated view of the region containing the **596U>C (m.1243U>C)** variant (yellow and labeled in bold, black font). Other rRNA residues are labeled in regular font. 12S mt-rRNA is shown in grey with helix numbers indicated. Other components of the mito-ribosome are labeled, and color coded to their molecular model. The position of R36 of MRPS14/uS14m is indicated. Hydrogen bonds are indicated by blue, broken lines. Distances are denoted with white, broken lines. Water molecules are shown as red spheres. **C.** Chimera X-rendered electron density (black mesh) at contour level of 5.88 (Pettersen et al.,

2021). The molecular model and electron density map from the 2.59-Å cryo-EM human mito-ribosomal structure (RCSB ID: 6ZM6) (Itoh et al., 2021) were used to create panels **B-D**.

Supplementary Figure S14

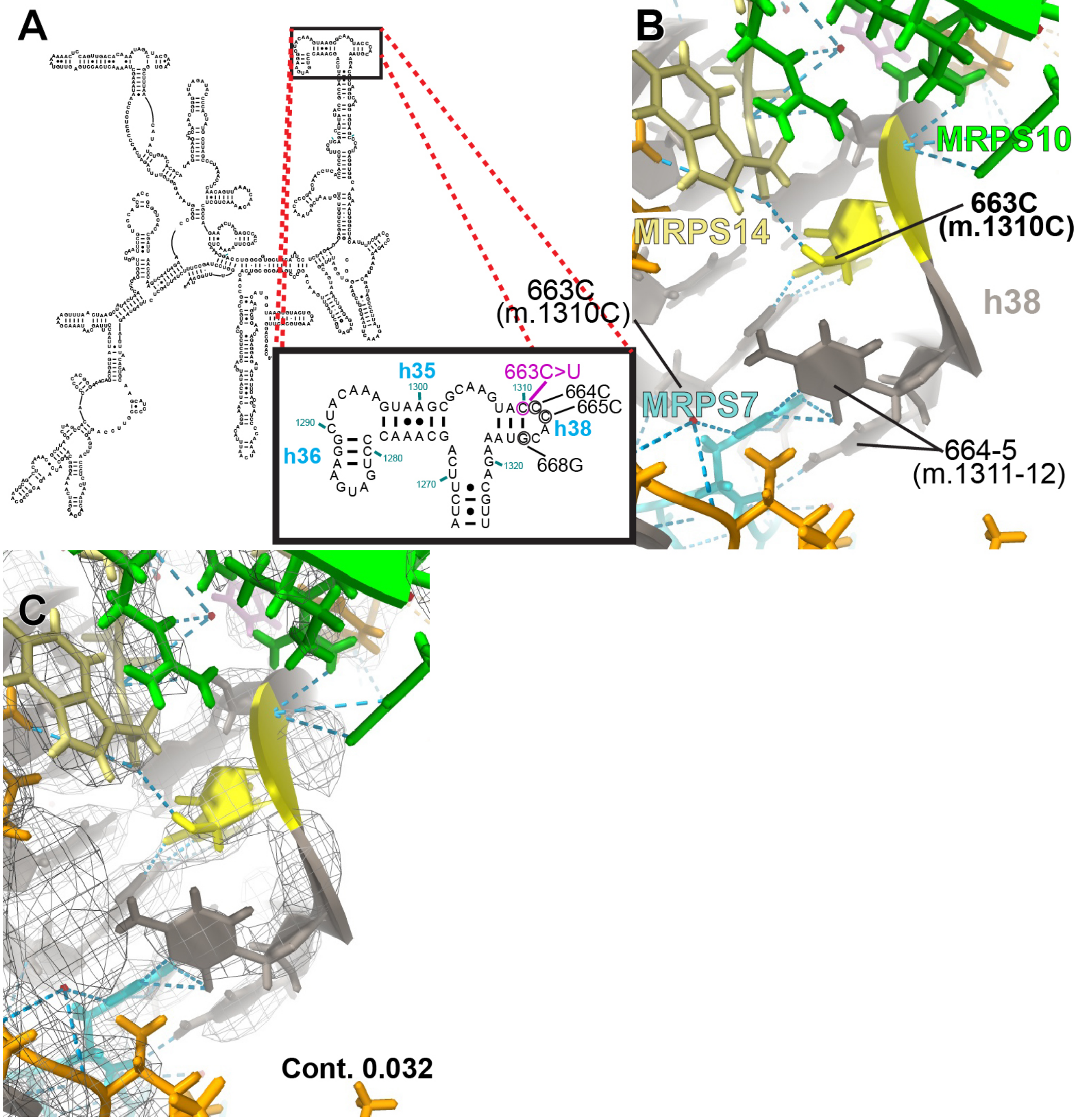

Supplementary Figure S14. Position 663C (m.1310C) in the human mito-ribosome. A. Localization of the variant-containing region in the secondary structure map of 12S mt-rRNA. Sites of variation are labeled in magenta. Additional sites are labeled in black. Helix numbers are shown in light blue. Symbols: "-", canonical base pair; "•", wobble base pair; "●", non-canonical base pair; thick, black line, physical connection and continuity between adjacent bases that are drawn distantly in the secondary-structure map; red squares connected by red, thick lines, tertiary interactions. **B.** Annotated view of the region containing the **663C>U (m.1310C>U)** variant (yellow and labeled in bold, black font). Other rRNA residues labeled in regular font. 12S mt-rRNA is shown in grey with helix numbers indicated. Other components of the mito-ribosome are labeled, and color coded to their molecular model. Hydrogen bonds are indicated by blue, broken lines. Distances are denoted with white, broken lines. Water molecules are shown as red spheres. **C.** Chimera X-rendered electron density (black mesh) at contour level of 0.032 (**C**) and 0.022 (**D**) (Pettersen et al., 2021). The molecular model and electron

density map from the 2.2-Å cryo-EM human mito-ribosomal structure (RCSB ID: 8ANY) (Itoh et al., 2021, Itoh et al., 2022) were used to create panels **B**-**D**.

Supplementary Figure S15

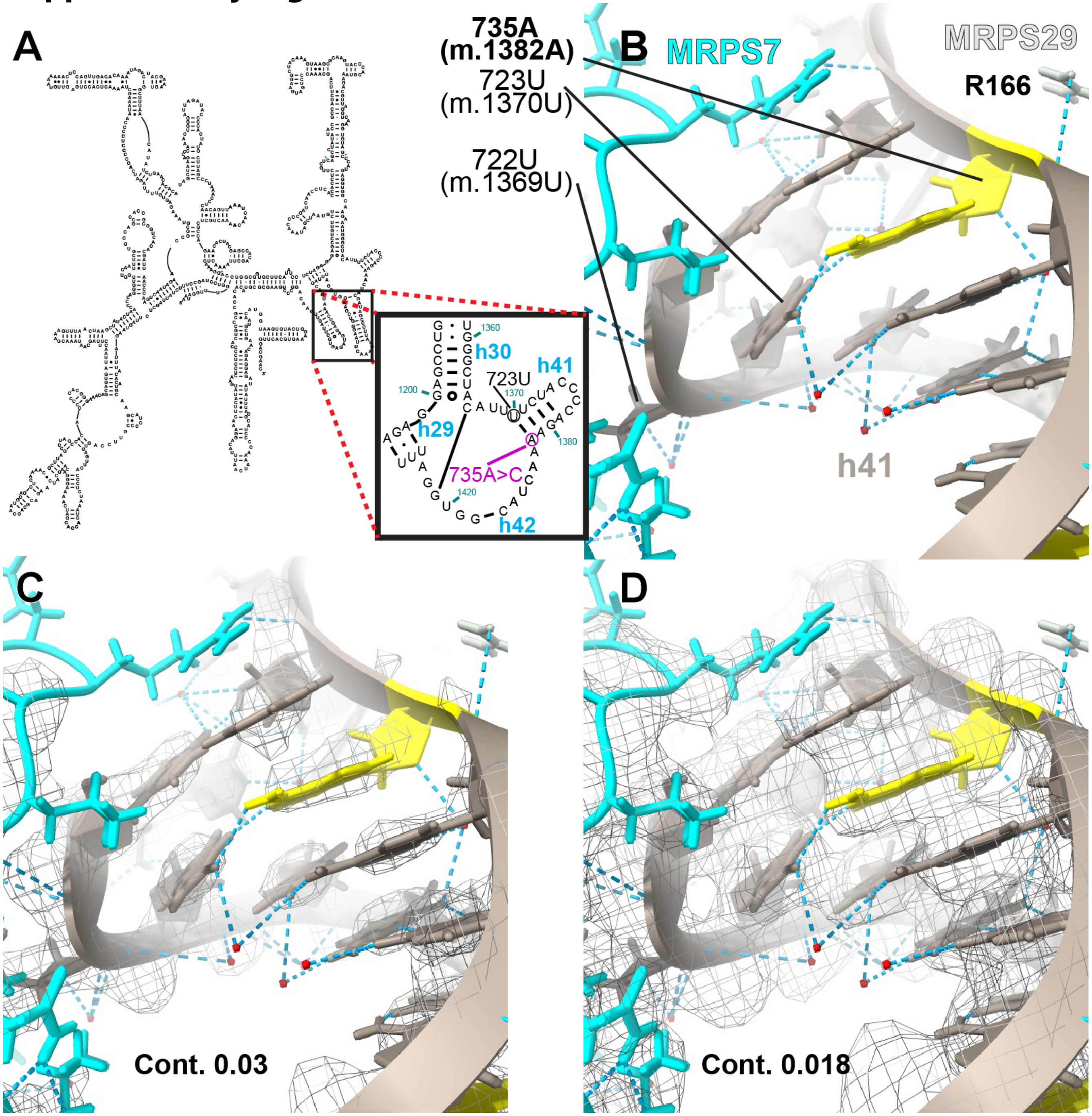

Supplementary Figure S15. Position 735A (m.1382A) in the human mito-ribosome. A. Localization of the variant-containing region in the secondary structure map of 12S mt-rRNA. Sites of variation are labeled in magenta. Additional sites are labeled in black. Helix numbers are shown in light blue. Symbols: "-", canonical base pair; "•", wobble base pair; "●", non-canonical base pair; thick, black line, physical connection and continuity between adjacent bases that are drawn distantly in the secondary-structure map; red squares connected by red, thick lines, tertiary interactions. **B.** Annotated view of the region containing the **735A>C (m.1382A>C)** variant (yellow and labeled in bold, black font). Other rRNA residues labeled in regular font. 12S mt-rRNA is shown in grey with helix numbers indicated. Other components of the mito-ribosome are labeled and color coded to their molecular model. The position of R166 of MRPS29/mS29 is indicated. Hydrogen bonds are indicated by blue, broken lines. Distances are denoted with white, broken lines. Water molecules are shown as red spheres. **C.** Chimera X-rendered electron density (black mesh) at contour level of 0.03 (**C**) and 0.018 (**D**), the latter used to demonstrate the existence of clear electron density around protein MRPS7/uS7m (Pettersen et al., 2021). The molecular model and

electron density map from the 2.2-Å cryo-EM human mito-ribosomal structure (RCSB ID: 8ANY) (Itoh et al., 2021, Itoh et al., 2022) were used to create panels **B**-**D**.

Supplementary Figure S16

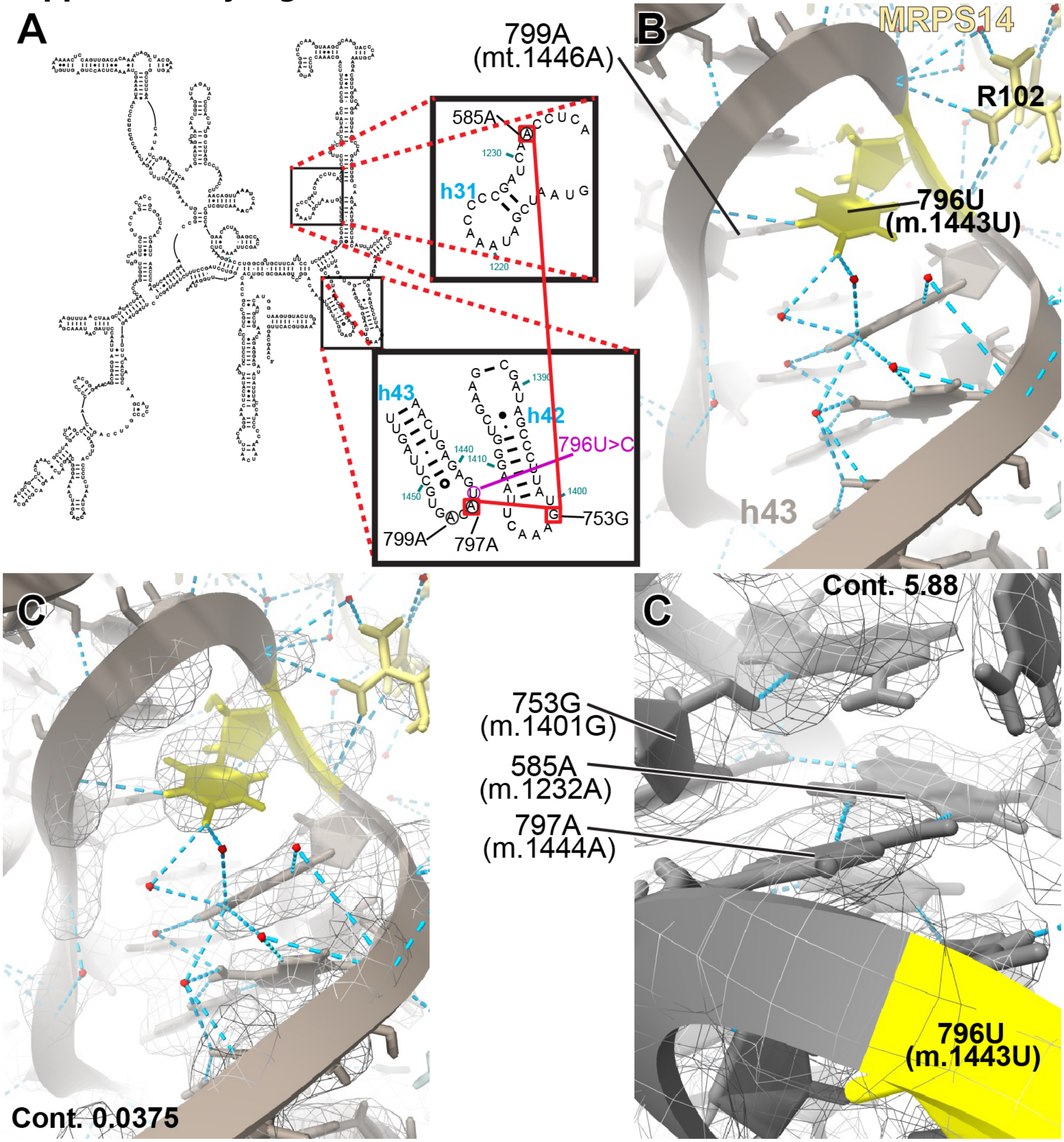

Supplementary Figure S16. Position 796U (m.1443U) in the human mito-ribosome. A. Localization of the variant-containing region in the secondary structure map of 12S mt-rRNA. Sites of variation are labeled in magenta. Additional sites are labeled in black. Helix numbers are shown in light blue. Symbols: "-", canonical base pair; "•", wobble base pair; "●", non-canonical base pair; thick, black line, physical connection and continuity between adjacent bases that are drawn distantly in the secondary-structure map; red squares connected by red, thick lines, tertiary interactions. **B.** Annotated view of the region containing the **796U>C (m.1443U>C)** variant (yellow and labeled in bold, black font). Other rRNA residues labeled in regular font. 12S mt-rRNA is shown in grey with helix numbers indicated. Other components of the mito-ribosome are labeled, and color coded to their molecular model. The position of R102 of MRPS14/uS14m is indicated. Hydrogen bonds are indicated by blue, broken lines. Distances are denoted with white, broken lines. Water molecules are shown as red spheres. **C.** Chimera X-rendered electron density (black mesh) at contour level of 0.0375 (Pettersen et al., 2021). **D.**

Chimera X-rendered electron density (black mesh) at contour level of 5.88 (Pettersen et al., 2021). The molecular model and electron density map from the 2.2-Å cryo-EM human mito-ribosomal structure (Itoh et al., 2021, Itoh et al., 2022) were used to create panels **B** and **C**. The molecular model and electron density map from the 2.59-Å cryo-EM human mito-ribosomal structure (RCSB ID: 6ZM6) (Itoh et al., 2021) were used to create panel **D**.

Supplementary Figure S17

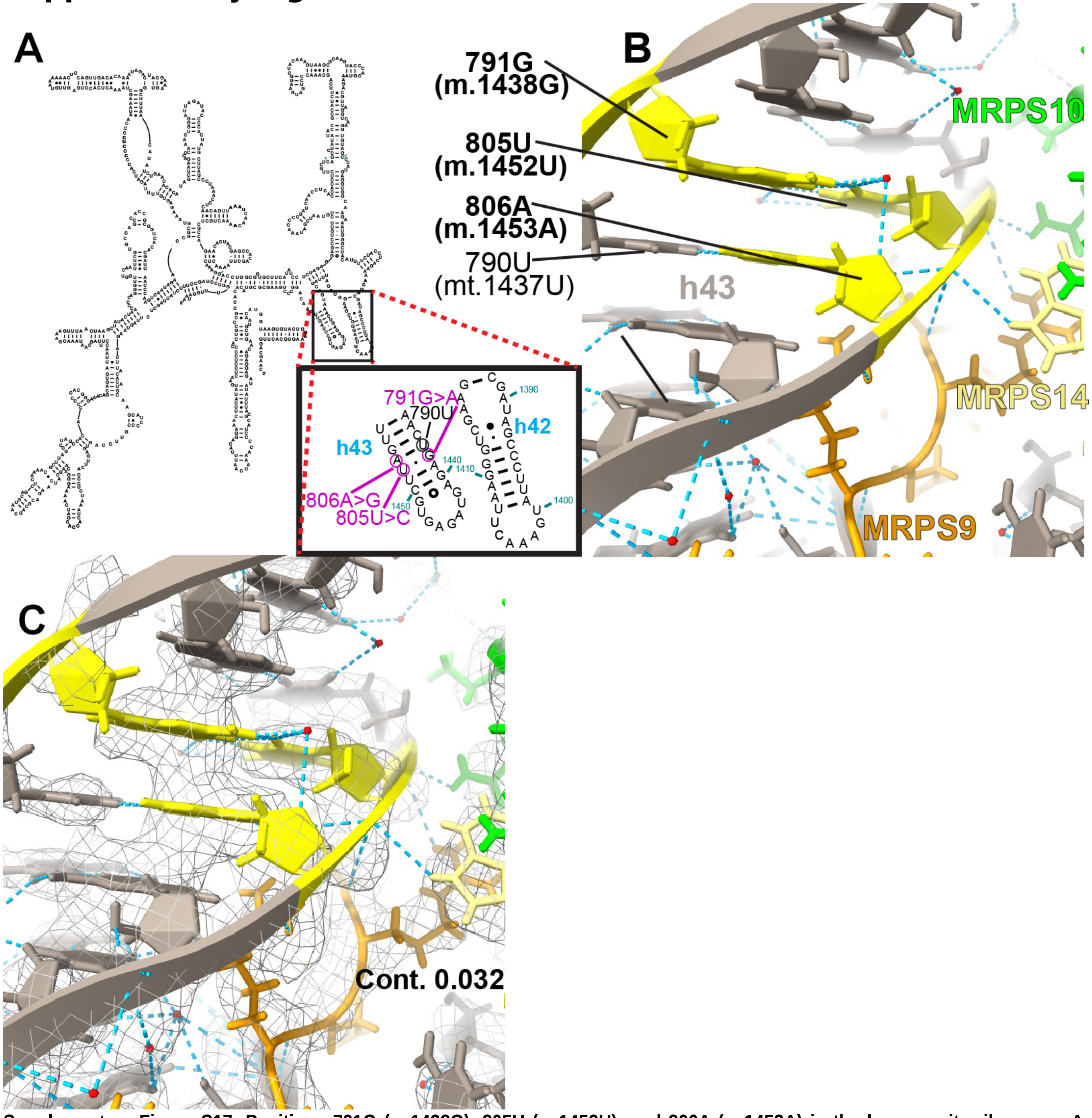

Supplementary Figure S17. Positions 791G (m.1438G), 805U (m.1452U), and 806A (m.1453A) in the human mito-ribosome. A. Localization of the variant-containing region in the secondary structure map of 12S mt-rRNA. Sites of variation are labeled in magenta. Additional sites are labeled in black. Helix numbers are shown in light blue. Symbols: "-", canonical base pair; "•", wobble base pair; "●", non-canonical base pair; thick, black line, physical connection and continuity between adjacent bases that are drawn distantly in the secondary-structure map; red squares connected by red, thick lines, tertiary interactions. **B.** Annotated view of the region containing the **791G>A (m.1438G>A)**, **805U>C (m.1452U>C)**, and **806A>G (m.1453A>G)** variants (yellow and labeled in bold, black font). Other rRNA residues are labeled in regular font. 12S mt-rRNA is shown in grey with helix numbers indicated. Other components of the mito-ribosome are labeled, and color coded to their molecular model. Hydrogen bonds are indicated by blue, broken lines. Distances are denoted with white, broken lines. Water molecules are shown as red spheres. **C.** Chimera X-rendered electron density (black mesh) at contour level of 0.032 (Pettersen et al., 2021). The molecular model and electron density map from the 2.2-Å cryo-EM human mito-ribosomal structure (RCSB ID: 8ANY) (Itoh et al., 2021, Itoh et al., 2022) were used to create panels **B-D**.

Supplementary Figure S18

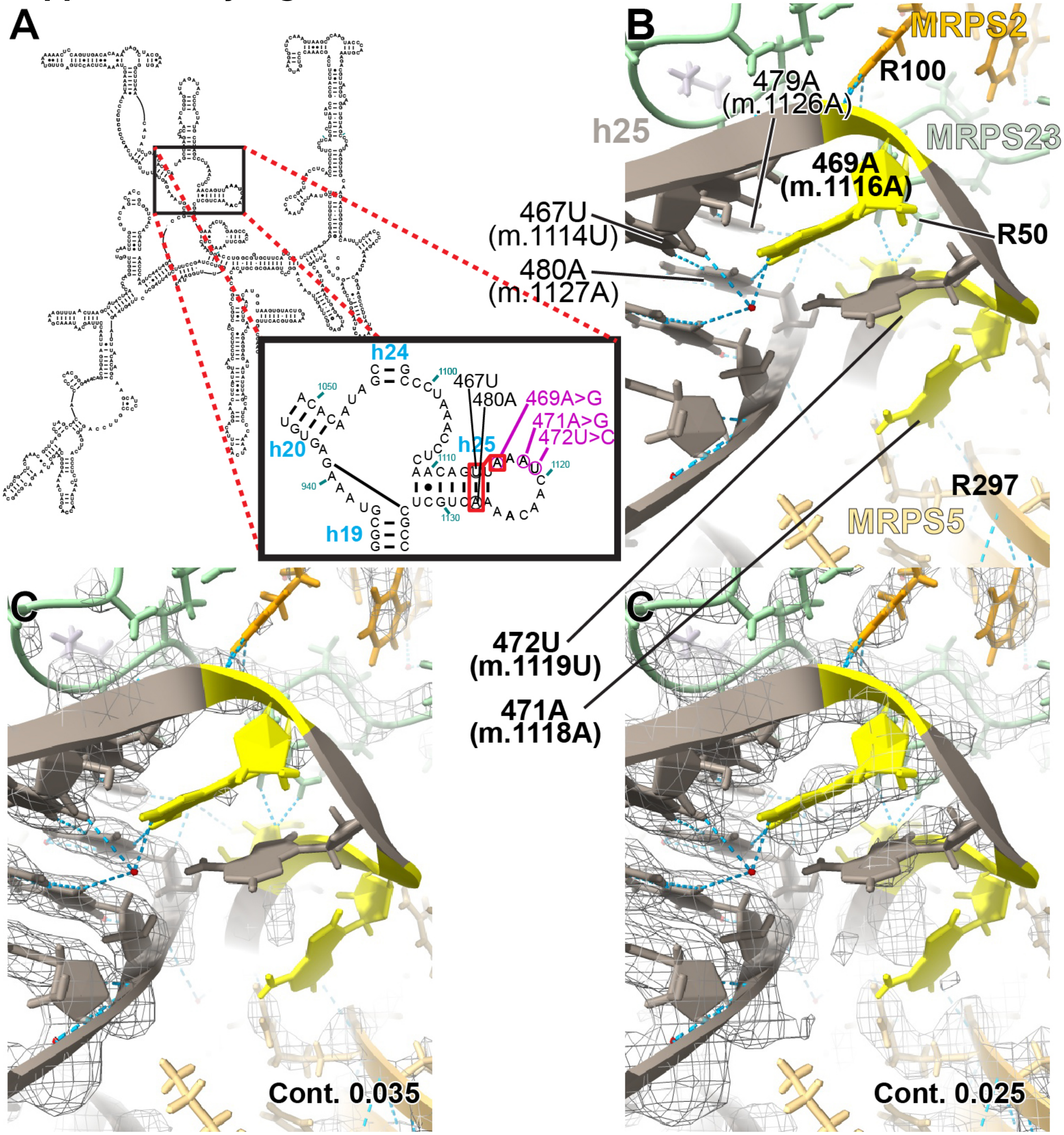

Supplementary Figure S18. Position 469A (m.1116A), 471A (m.1118A), and 472U (m.1119U) in the human mito-ribosome. A. Localization of the variant-containing region in the secondary structure map of 12S mt-rRNA. Sites of variation are labeled in magenta. Additional sites are labeled in black. Helix numbers are shown in light blue. Symbols: "-", canonical base pair; "•", wobble base pair; "●", non-canonical base pair; thick, black line, physical connection and continuity between adjacent bases that are drawn distantly in the secondary-structure map; red squares connected by red, thick lines, tertiary interactions. **B.** Annotated view of the region containing the **469A>G (m.1116A>G), 471A>G (m.1118A>G),** and **472U>C (m.1119U>C)** variant (yellow and labeled in bold, black font). Other rRNA residues labeled in regular font. 12S mt-rRNA is shown in grey with helix numbers indicated. Other components of the mito-ribosome are labeled, and color coded to their molecular model. The positions of R100 of MRPS2/uS2m, R297 of MRPS5/uS5m, and R50 of MRPS23/uS23 are indicated. Hydrogen bonds are indicated by blue, broken lines. Distances are denoted with white, broken lines. Water

molecules are shown as red spheres. **C** and **D.** Chimera X-rendered electron density (black mesh) at contour level of 0.035 (**C**) and 0.025 (**D**), the latter used to demonstrate the existence of clear electron density around position **469A (m.1116A)** (Pettersen et al., 2021). The molecular model and electron density map from the 2.2-Å cryo-EM human mito-ribosomal structure (RCSB ID: 8ANY) (Itoh et al., 2021, Itoh et al., 2022) were used to create panels **B-D**.

Supplementary Figure S19

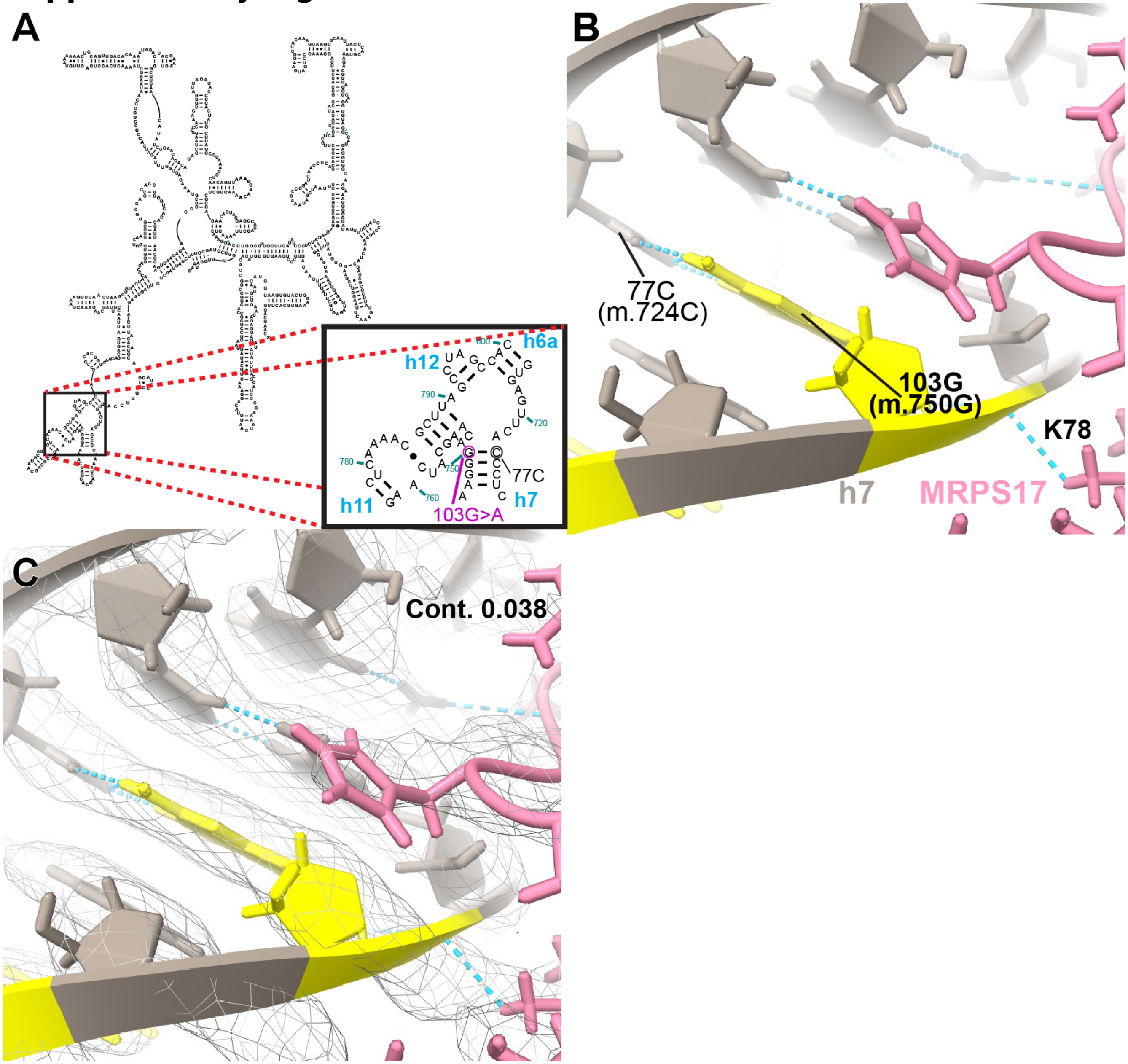

Supplementary Figure S19. Position 103G (m.750G) in the human mito-ribosome. A. Localization of the variant-containing region in the secondary structure map of 12S mt-rRNA. Sites of variation are labeled in magenta. Additional sites are labeled in black. Helix numbers are shown in light blue. Symbols: "-", canonical base pair; "•", wobble base pair; "●", non-canonical base pair; thick, black line, physical connection and continuity between adjacent bases that are drawn distantly in the secondary-structure map; red squares connected by red, thick lines, tertiary interactions. **B.** Annotated view of the region containing the **103G>A (m.750G>A)** variant (yellow and labeled in bold, black font). Other rRNA residues labeled in regular font. 12S mt-rRNA is shown in grey with helix numbers indicated. Other components of the mito-ribosome are labeled, and color coded to their molecular model. The position of K78 of MRPS17/uS17m is indicated. Hydrogen bonds are indicated by blue, broken lines. Distances are denoted with white, broken lines. Water molecules are shown as red spheres. **C.** Chimera X-rendered electron density (black mesh) at contour level of 0.038 (Pettersen et al., 2021). The molecular model and electron density map from the 2.2-Å cryo-EM human mito-ribosomal structure (RCSB ID: 8ANY) (Itoh et al., 2021, Itoh et al., 2022) were used to create panels **B-C**.

Supplementary Figure S20

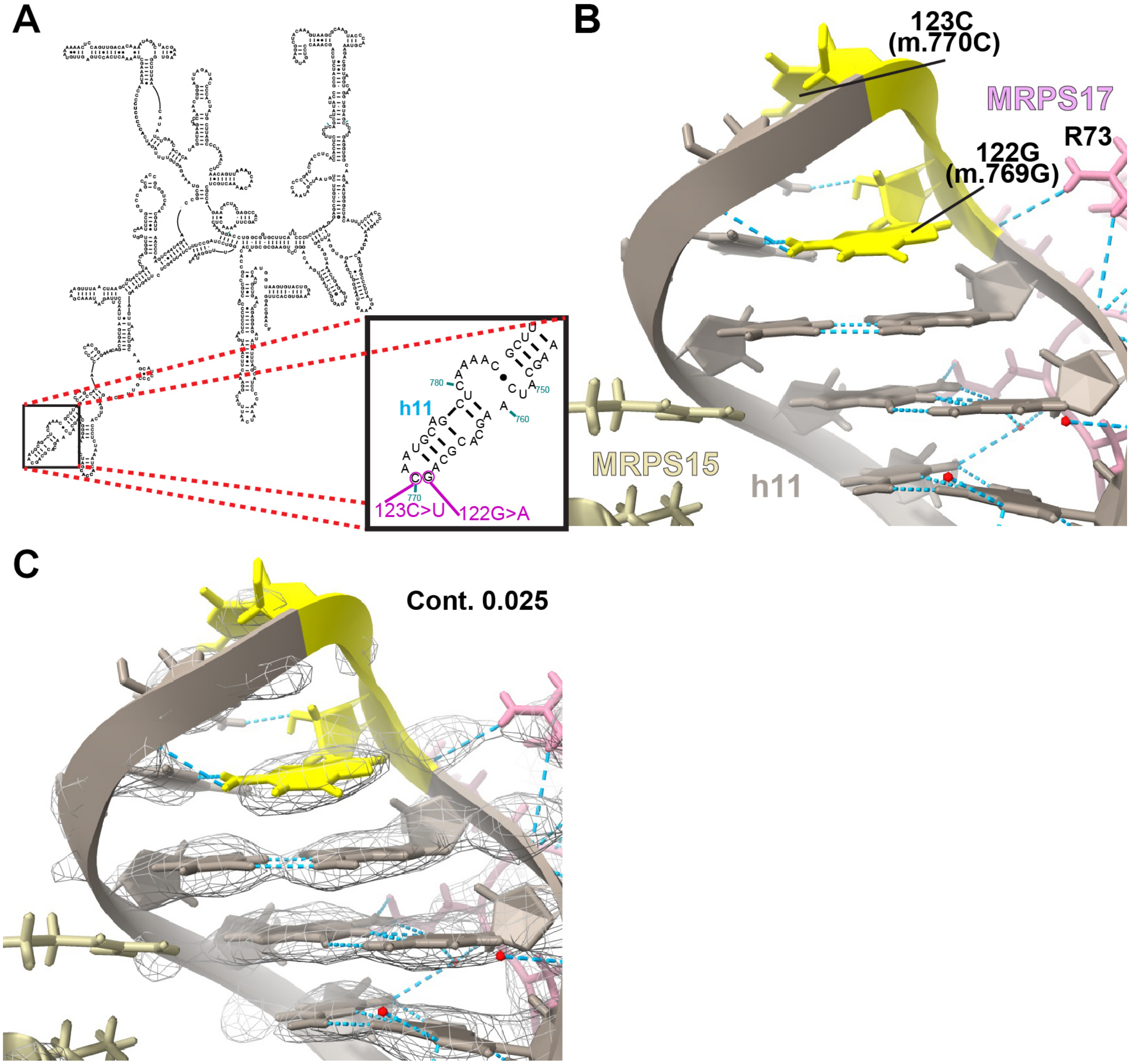

Supplementary Figure S20. Positions 122G (m.769G) and 123C (m.770C) in the human mito-ribosome. A. Localization of the variant-containing region in the secondary structure map of 12S mt-rRNA. Sites of variation are labeled in magenta. Additional sites are labeled in black. Helix numbers are shown in light blue. Symbols: "-", canonical base pair; "•", wobble base pair; "●", non-canonical base pair; thick, black line, physical connection and continuity between adjacent bases that are drawn distantly in the secondary-structure map; red squares connected by red, thick lines, tertiary interactions. **B.** Annotated view of the region containing the **122G>A (m.769G>A)** and **123C>U (m.770C>U)** variants (yellow and labeled in bold, black font). Other rRNA residues labeled in regular font. 12S mt-rRNA is shown in grey with helix numbers indicated. Other components of the mito-ribosome are labeled, and color coded to their molecular model. The position of R73 of MRPS17/uS17m is indicated. Hydrogen bonds are indicated by blue, broken lines. Distances are denoted with white, broken lines. Water molecules are shown as red spheres. **C.** Chimera X-rendered electron density (black mesh) at contour level of 0.025 (Pettersen et al., 2021). The molecular model and electron density map from the 2.2-Å cryo-EM human mito-ribosomal structure (RCSB ID: 8ANY) (Itoh et al., 2021, Itoh et al., 2022) were used to create panels **B-C**.

Supplementary Figure S21

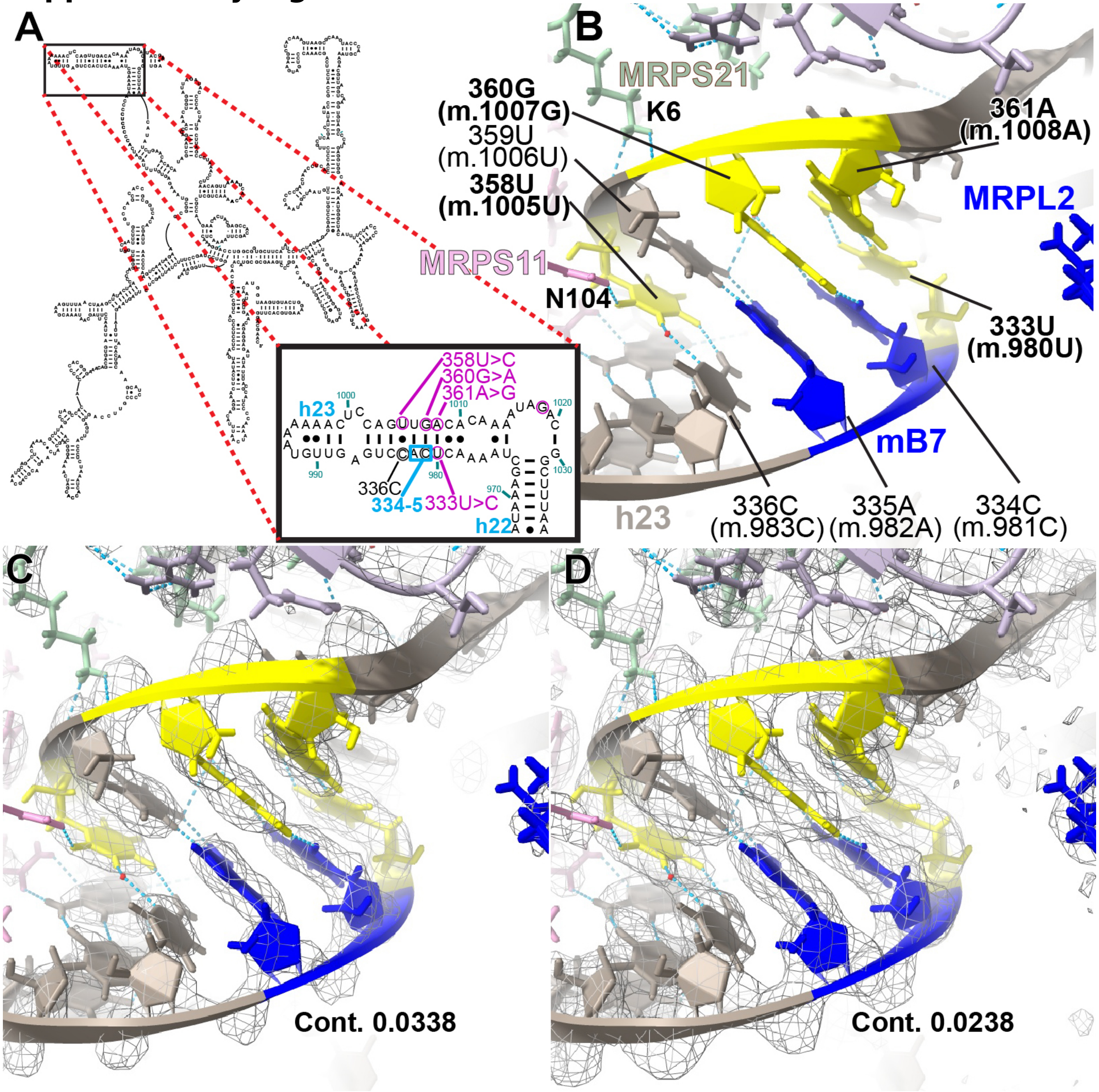

Supplementary Figure S21. Positions 333U (m.980U), 358U (m.1005U), 360G>A (m.1007G), and 361A (m.1008A) in the human mito-ribosome. A. Localization of the variant-containing region in the secondary structure map of 12S mt-rRNA. Sites of variation are labeled in magenta. Additional sites are labeled in black. Helix numbers are shown in light blue. Symbols: "-", canonical base pair; "•", wobble base pair; "●", non-canonical base pair; thick, black line, physical connection and continuity between adjacent bases that are drawn distantly in the secondary-structure map; red squares connected by red, thick lines, tertiary interactions; cyan squares, bridge residues. **B.** Annotated view of the region containing the **333U>C (m.980U>C)**, **358U>C (m.1005U>C)**, **360G>A (m.1007G>A)**, and **361A>G (m.1008A>G)** variants (yellow and labeled in bold, black font). Bridge residues are shown in blue. Other rRNA residues are labeled in regular font. 12S mt-rRNA is shown in grey with helix numbers indicated. Other components of the mito-ribosome are labeled and color coded to their molecular model. The positions of N104 of MRPS11/uS11 and K6 of MRPS21/bS21 are indicated. Hydrogen bonds are indicated by blue, broken lines. Distances are denoted with white, broken lines. Water molecules are shown as red spheres. **C.** Chimera X-rendered electron density (black mesh) at contour level of 0.0338 (**C**) and 0.0238 (**D**), the latter used to demonstrate the

existence of clear electron density around MRPS21/bS21 (Pettersen et al., 2021). The molecular model and electron density map from the 2.2-Å cryo-EM human mito-ribosomal structure (RCSB ID: 8ANY) (Itoh et al., 2021, Itoh et al., 2022) were used to create panels **B**-**D**.

Supplementary Figure S22

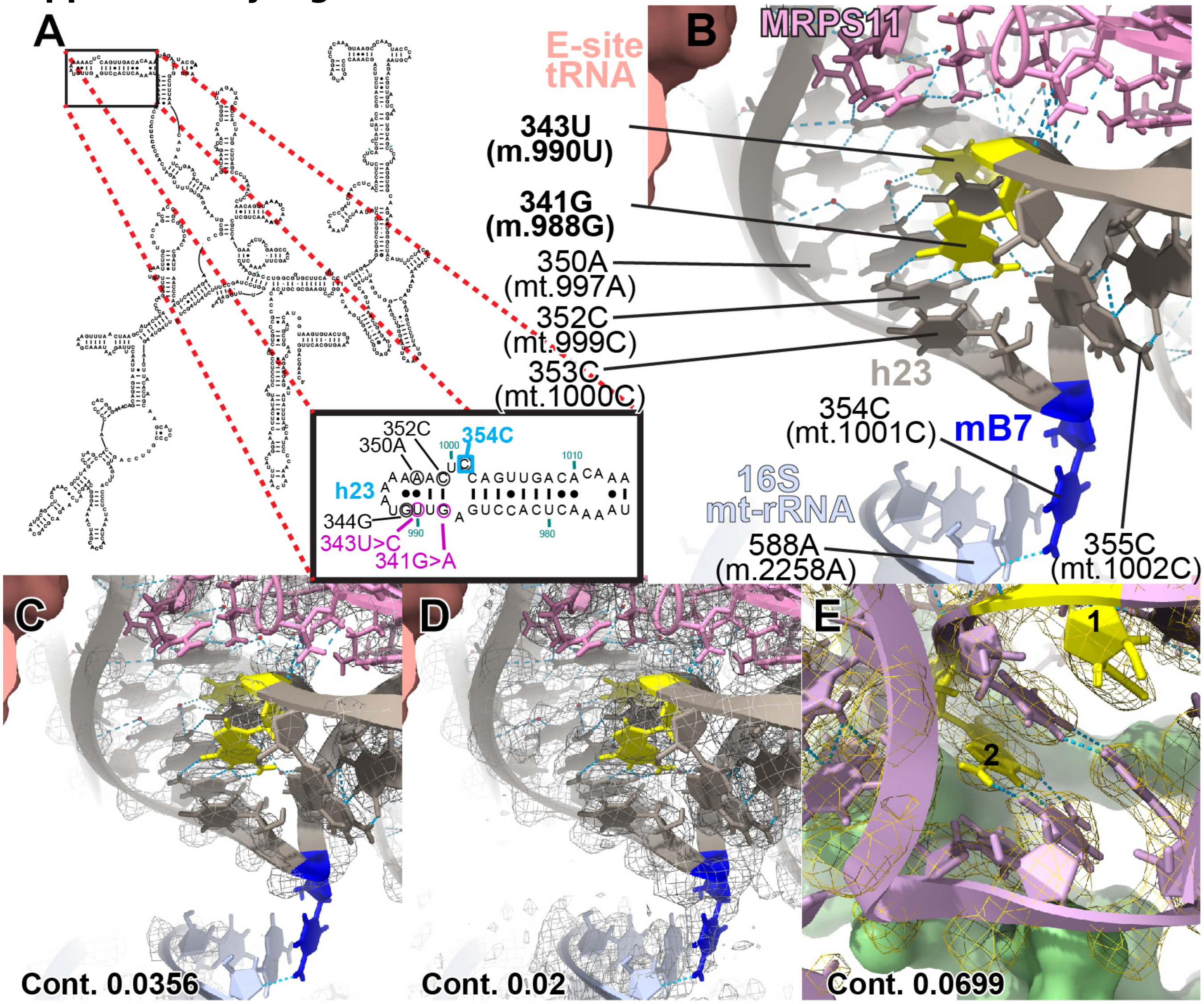

Supplementary Figure S22. Positions 341G (m.988G) and 343U (m.990U) in the human mito-ribosome. A. Localization of the variant-containing region in the secondary structure map of 12S mt-rRNA. Sites of variation are labeled in magenta. Additional sites are labeled in black. Helix numbers are shown in light blue. Symbols: "-", canonical base pair; "•", wobble base pair; "●", non-canonical base pair; thick, black line, physical connection and continuity between adjacent bases that are drawn distantly in the secondary-structure map; red squares connected by red, thick lines, tertiary interactions; cyan squares, bridge residues. **B.** Annotated view of the region containing the **341G>A (m.988G>A)** and **343U>C (m.990U>C)** variants (yellow and labeled in bold, black font). Bridge residues are shown in blue. Other rRNA residues labeled in regular font. 12S mt-rRNA is shown in grey with helix numbers indicated. Other components of the mito-ribosome are labeled, and color coded to their molecular model. Hydrogen bonds are indicated by blue, broken lines. Distances are denoted with white, broken lines. Water molecules are shown as red spheres. **C-D.** Chimera X-rendered electron density (black mesh) at contour level of 0.0356 (**C**), 0.0238 (**D**), the latter used to demonstrate the existence of clear electron density around bridge residues (Pettersen et al., 2021). **E.** The molecular model and electron density map from the 2.2-Å cryo-EM human mito-ribosomal structure (Itoh et al., 2021, Itoh et al., 2022) were used to create panels **B**-**D**. **E.** Structure of the **341G-343U (m.988G-990U)** region in the 2.97-Å structure of the human mitochondrial 28S ribosome in complex with mitochondrial IF3 (RCSB ID: 6WR5) (Khawaja et al., 2020). Variant residues shown in yellow: **1**, **343U (m.990U)**; **2**, **341G (m.988G)**. 12S mt-rRNA, pink; MTIF3, light green surface; electron density contour level, 0.0699.

Supplementary Figure S23

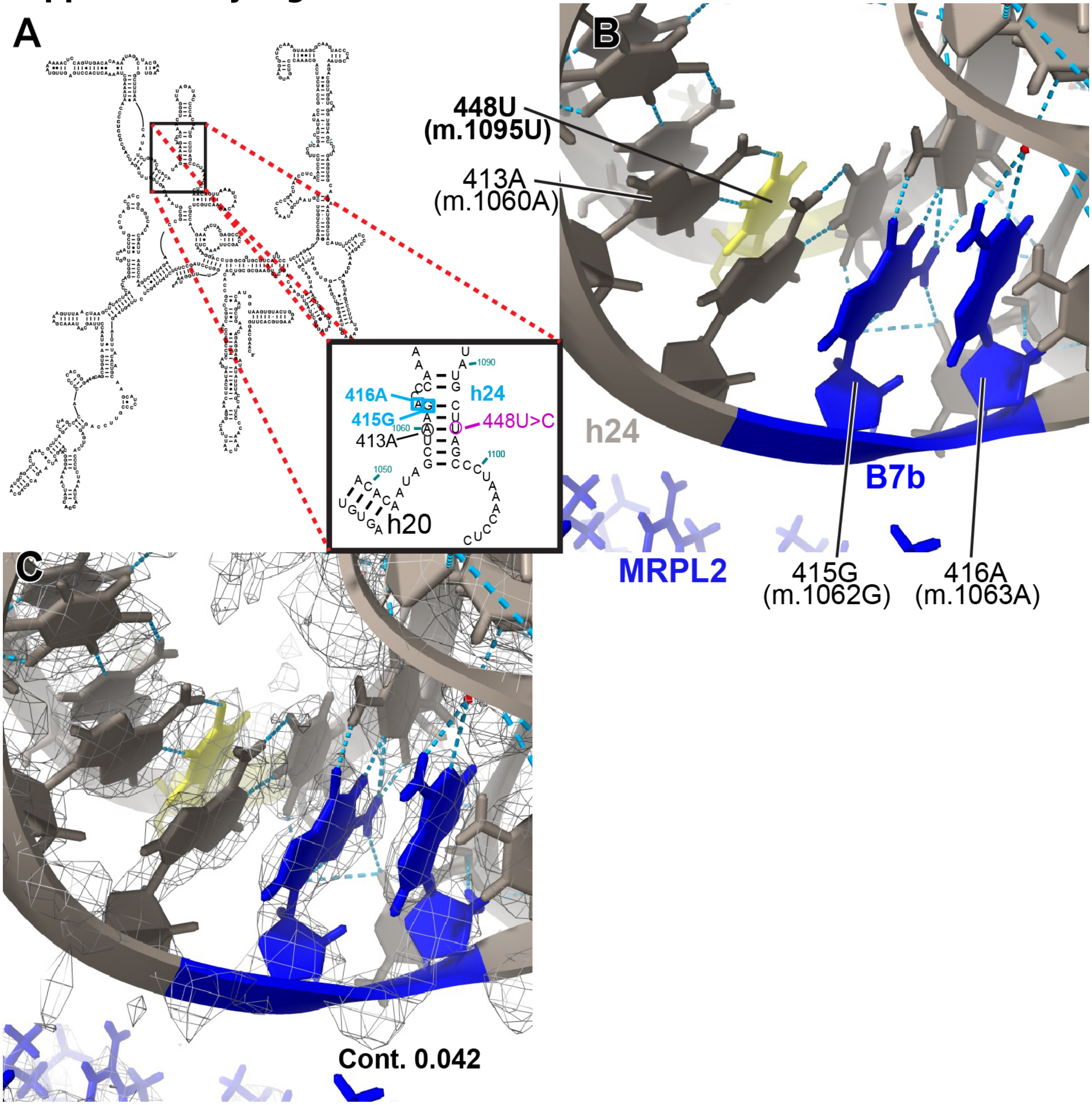

Supplementary Figure S23. Position 448U (m.1095U) in the human mito-ribosome. A. Localization of the variant-containing region in the secondary structure map of 12S mt-rRNA. Sites of variation are labeled in magenta. Additional sites are labeled in black. Helix numbers are shown in light blue. Symbols: "-", canonical base pair; "•", wobble base pair; "●", non-canonical base pair; thick, black line, physical connection and continuity between adjacent bases that are drawn distantly in the secondary-structure map; red squares connected by red, thick lines, tertiary interactions; cyan squares, bridge residues. **B.** Annotated view of the region containing the **448U>C (m.1095U>C)** variant (yellow and labeled in bold, black font). Bridge residues are shown in blue. Other rRNA residues labeled in regular font. 12S mt-rRNA is shown in grey with helix numbers indicated. Other components of the mito-ribosome are labeled, and color coded to their molecular model. Hydrogen bonds are indicated by blue, broken lines. Distances are denoted with white, broken lines. Water molecules are shown as red spheres. **C.** Chimera X-rendered electron density (black mesh) at contour level of 0.042 (Pettersen et al., 2021). The molecular model and electron density map from the 2.2-Å cryo-EM human mito-ribosomal structure (RCSB ID: 8ANY) (Itoh et al., 2021, Itoh et al., 2022) were used to create panels **B**-**D**.

Supplementary Figure S24

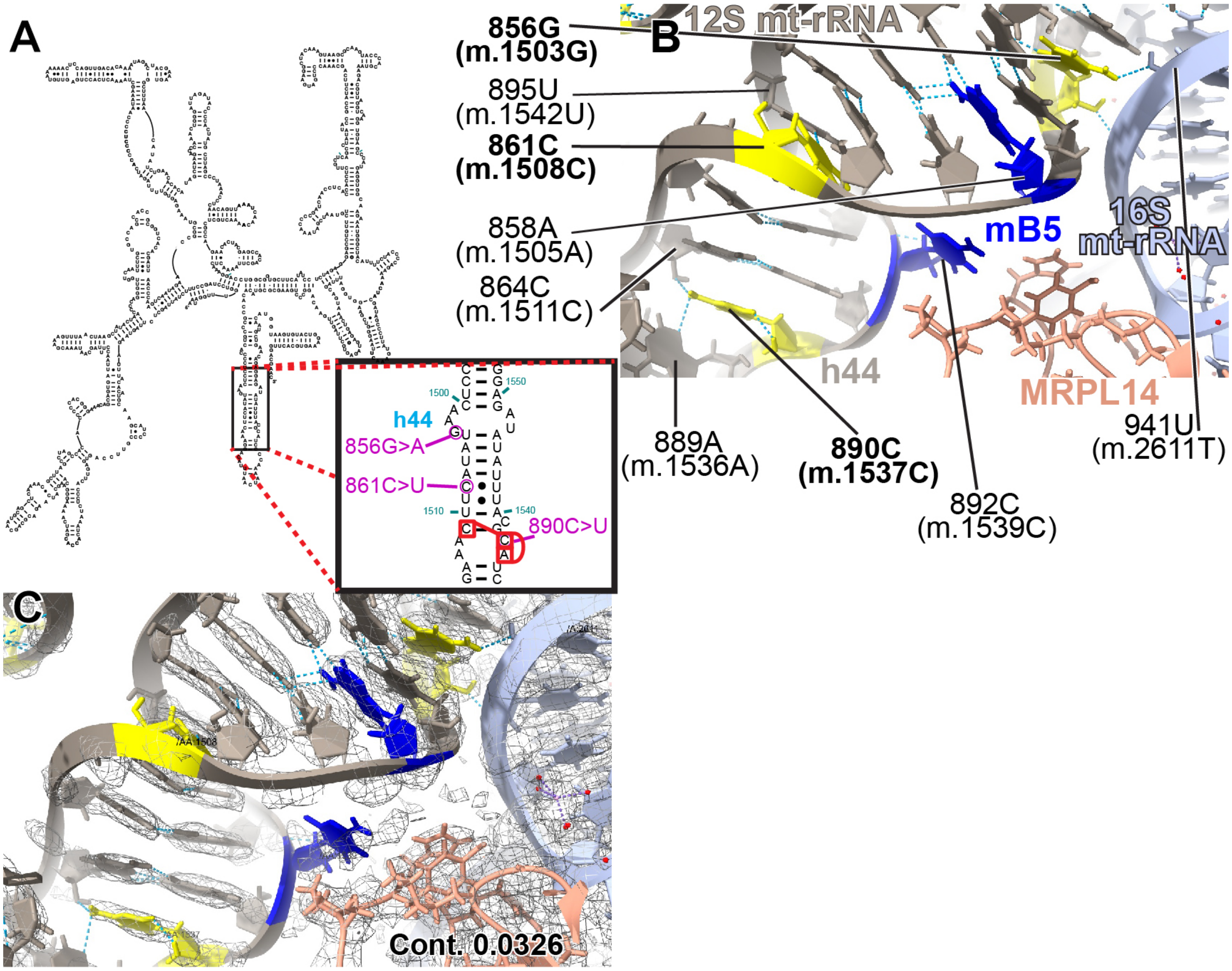

Supplementary Figure S24. Positions 856G (m.1503G), 861C (m.1508C) and 890C (m.1537C) in the human mito-ribosome. A. Localization of the variant-containing region in the secondary structure map of 12S mt-rRNA. Sites of variation are labeled in magenta. Additional sites are labeled in black. Helix numbers are shown in light blue. Symbols: "-", canonical base pair; "•", wobble base pair; "●", non-canonical base pair; thick, black line, physical connection and continuity between adjacent bases that are drawn distantly in the secondary-structure map; red squares connected by red, thick lines, tertiary interactions; cyan squares, bridge residues. **B.** Annotated view of the region containing the **856G>A (m.1503G>A), 861C>U (m.1508C>U),** and **890C>U (m.1537C>U)** variants (yellow and labeled in bold, black font). Bridge residues are shown in blue. Other rRNA residues labeled in regular font. 12S mt-rRNA is shown in grey with helix numbers indicated. Other components of the mito-ribosome are labeled and color coded to their molecular model. Hydrogen bonds are indicated by blue, broken lines. Distances are denoted with white, broken lines. Water molecules are shown as red spheres. **C.** Chimera X-rendered electron density (black mesh) at contour level of 0.0326 (Pettersen et al., 2021). The molecular model and electron density map from the 2.2-Å cryo-EM human mito-ribosomal structure (RCSB ID: 8ANY) (Itoh et al., 2021, Itoh et al., 2022) were used to create panels **B**-**D**.

Supplementary Figure S25

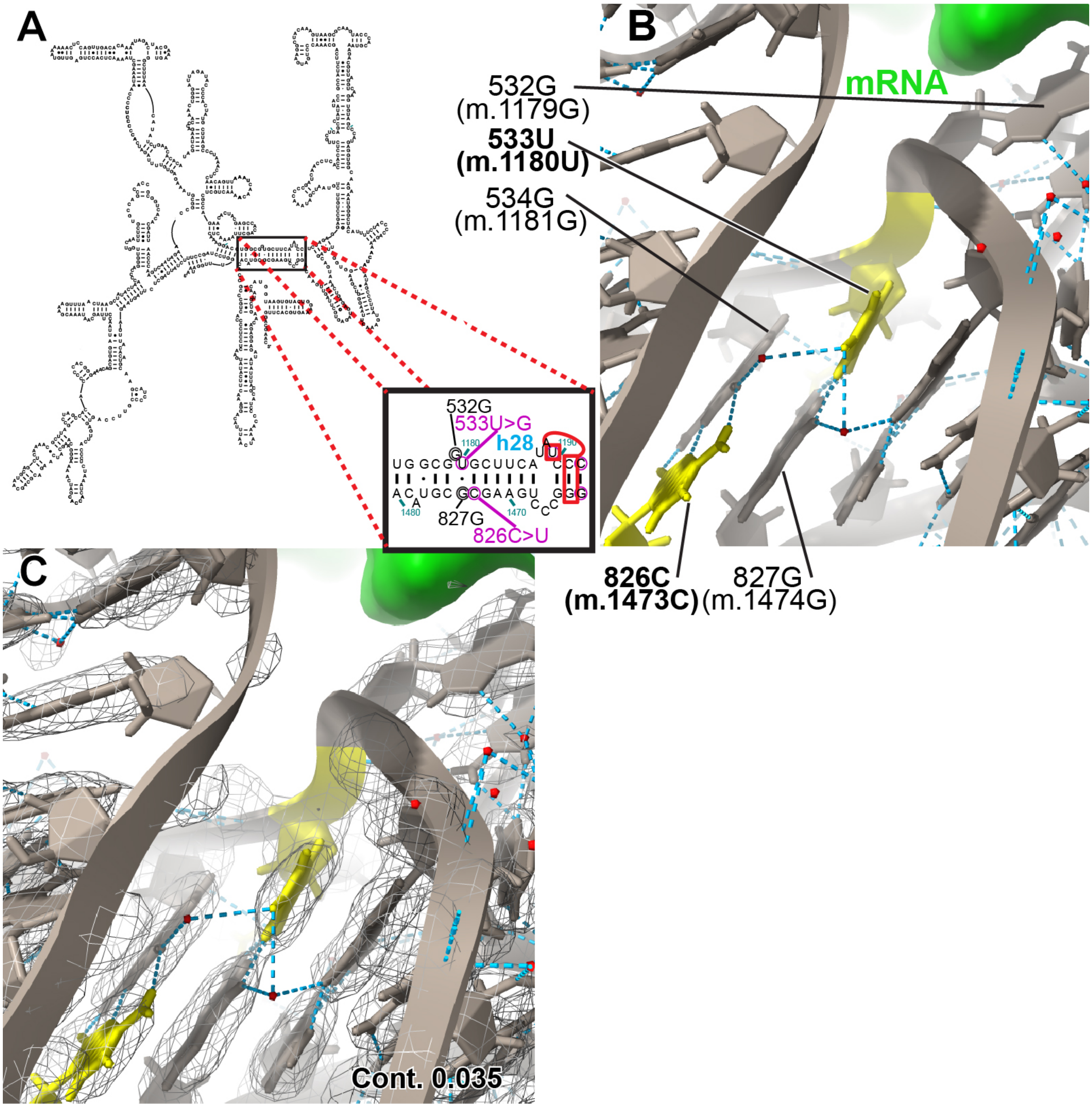

Supplementary Figure S25. Positions 533U (m.1180U) and 826C (m.1473C) in the human mito-ribosome. A. Localization of the variant-containing region in the secondary structure map of 12S mt-rRNA. Sites of variation are labeled in magenta. Additional sites are labeled in black. Helix numbers are shown in light blue. Symbols: "-", canonical base pair; "•", wobble base pair; "●", non-canonical base pair; thick, black line, physical connection and continuity between adjacent bases that are drawn distantly in the secondary-structure map; red squares connected by red, thick lines, tertiary interactions. **B.** Annotated view of the region containing the **533U>G (m.1180U>G)** and **826C>U (m.1473C>U)** variants (yellow and labeled in bold, black font). Other rRNA residues labeled in regular font. 12S mt-rRNA is shown in grey with helix numbers indicated. MRNA is shown as a lime surface. Other components of the mito-ribosome are labeled, and color coded to their molecular model. Hydrogen bonds are indicated by blue, broken lines. Distances are denoted with white, broken lines. Water molecules are shown as red spheres. **C.** Chimera X-rendered electron density (black mesh) at contour level of 0.035

(Pettersen et al., 2021). The molecular model and electron density map from the 2.2-Å cryo-EM human mito-ribosomal structure (RCSB ID: 8ANY) (Itoh et al., 2021, Itoh et al., 2022) were used to create panels **B**-**D**.

Supplementary Figure S26

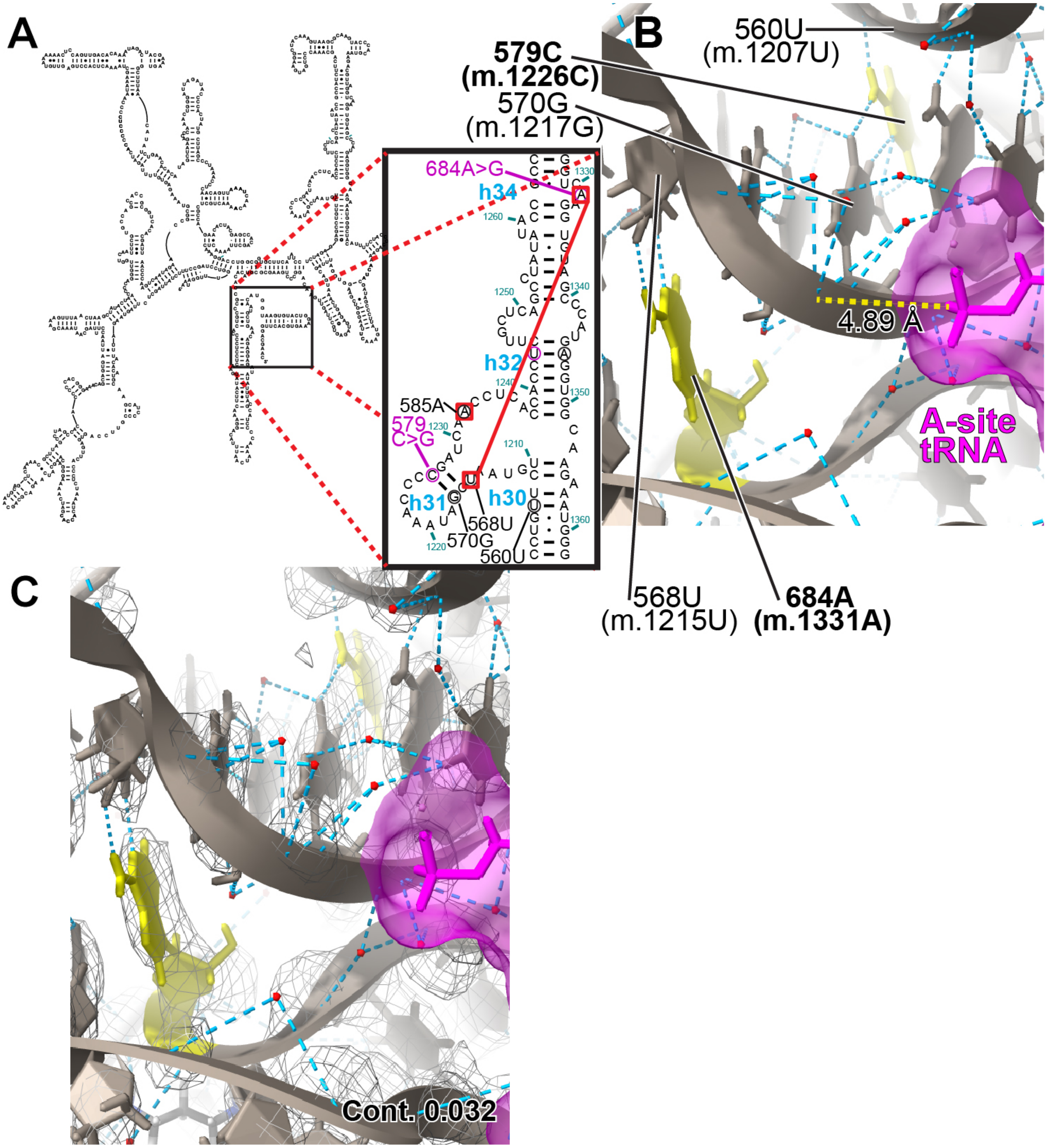

Supplementary Figure S26. Positions 579C (m.1226C) and 684A (m.1331A) in the human mito-ribosome. A. Localization of the variant-containing region in the secondary structure map of 12S mt-rRNA. Sites of variation are labeled in magenta. Additional sites are labeled in black. Helix numbers are shown in light blue. Symbols: "-", canonical base pair; "•", wobble base pair; "●", non-canonical base pair; thick, black line, physical connection and continuity between adjacent bases that are drawn distantly in the secondary-structure map; red squares connected by red, thick lines, tertiary interactions. **B.** Annotated view of the region containing the **579C>G (m.1226C>G)** and **684A>G (m.1331A>G)** variants (yellow and labeled in bold, black font). Other rRNA residues are labeled in regular font. 12S mt-rRNA is shown in grey with helix numbers indicated. A-site-tRNA is shown as a magenta semi-transparent surface containing the stick representation of its associated residues. Other components of the mitoribosome are labeled and color coded to their molecular model.

Hydrogen bonds are indicated by blue, broken lines. Distances are denoted with white, broken lines. Water molecules are shown as red spheres. **C.** Chimera X-rendered electron density (black mesh) at contour level of 0.032 (Pettersen et al., 2021). The molecular model and electron density map from the 2.2-Å cryo-EM human mito-ribosomal structure (RCSB ID: 8ANY) (Itoh et al., 2021, Itoh et al., 2022) were used to create panels **B**-**D**.

Supplementary Figure S27

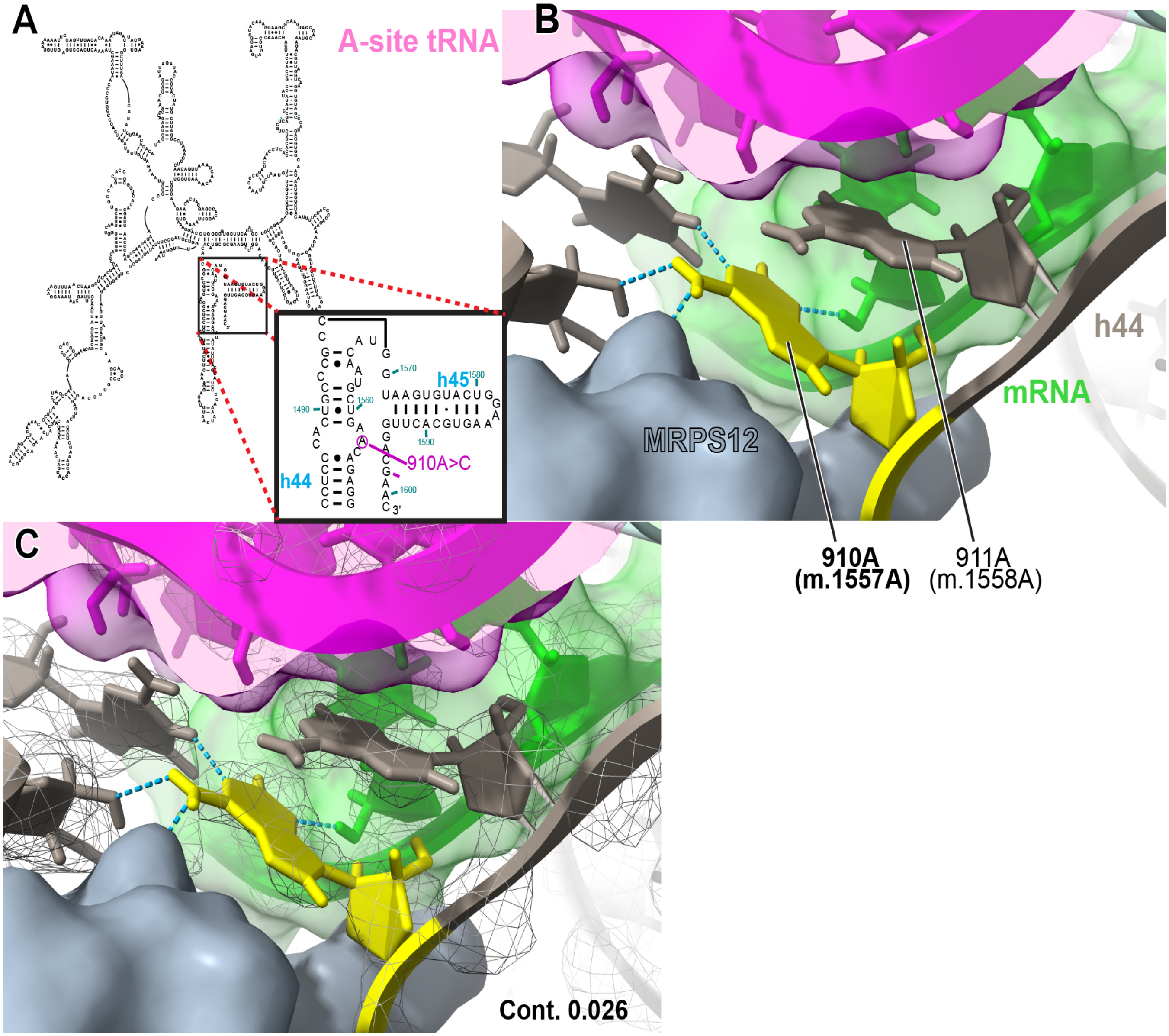

Supplementary Figure S27. Position 910A (m.1557A) in the human mito-ribosome. A. Localization of the variant-containing region in the secondary structure map of 12S mt-rRNA. Sites of variation are labeled in magenta. Additional sites are labeled in black. Helix numbers are shown in light blue. Symbols: "-", canonical base pair; "•", wobble base pair; "●", non-canonical base pair; thick, black line, physical connection and continuity between adjacent bases that are drawn distantly in the secondary-structure map; red squares connected by red, thick lines, tertiary interactions. **B.** Annotated view of the region containing the **910A>C (m.1557A>C)** variant (yellow and labeled in bold, black font). Other rRNA residues labeled in regular font. 12S mt-rRNA is shown in grey with helix numbers indicated. A-site-tRNA and mRNA are shown as semi-transparent surfaces, containing the ribbon and stick representation of their associated residues in their interior. A-site tRNA, magenta; mRNA, lime. MRPS12/uS12m is shown as a grey surface. Other components of the mito-ribosome are labeled, and color coded to their molecular model. Hydrogen bonds are indicated by blue, broken lines. Distances are denoted with white, broken lines. Water molecules are shown as red spheres. **C.** Chimera X-rendered electron density (black mesh) at contour level of 0.026 (Pettersen et al., 2021). The molecular model and electron density map from the 2.2-Å cryo-EM human mito-ribosomal structure (RCSB ID: 8ANY) (Itoh et al., 2021, Itoh et al., 2022) were used to create panels **B**-**D**.

Supplementary Figure S28

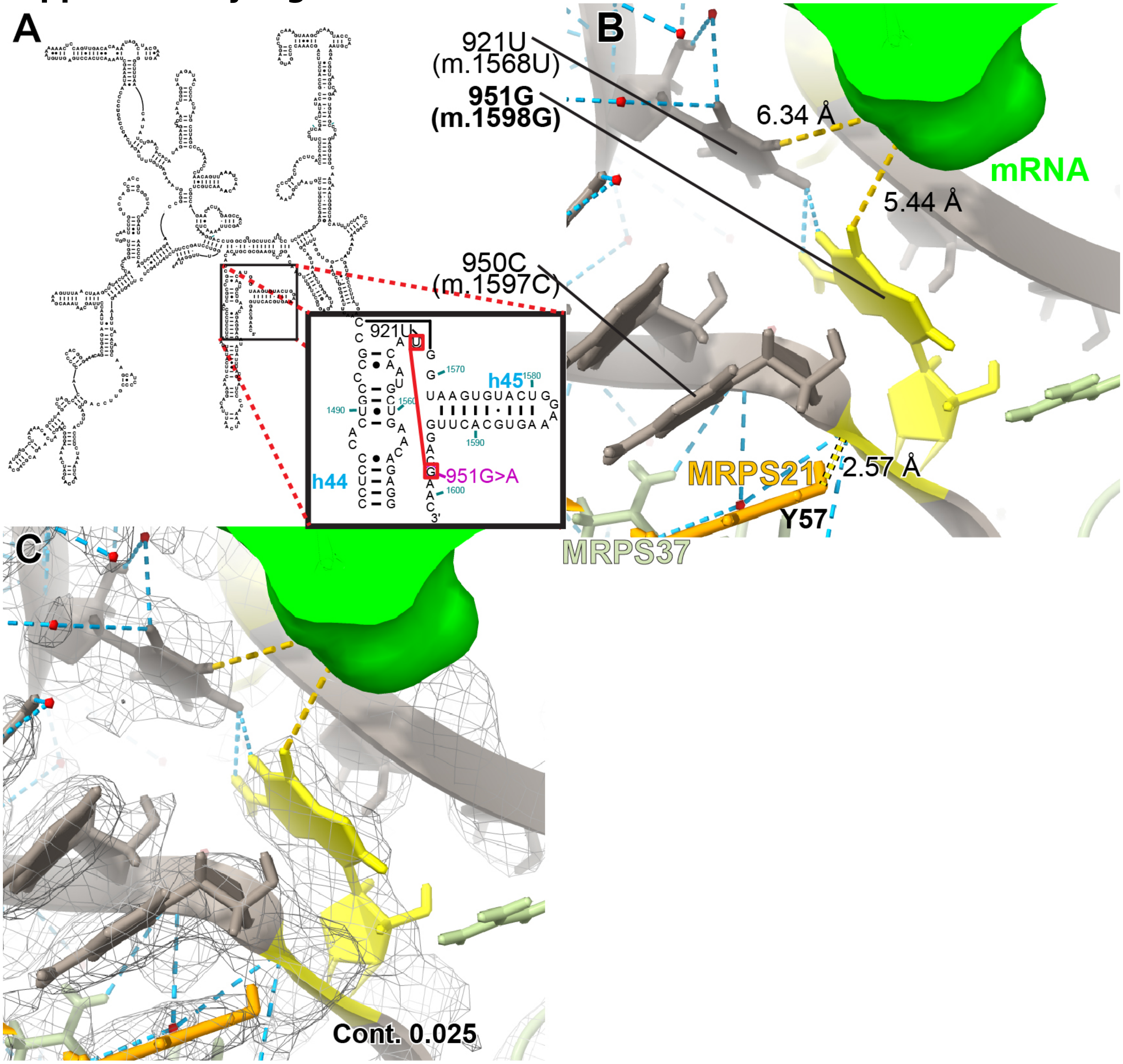

Supplementary Figure S28. Position 951G (m.1598G) in the human mito-ribosome. A. Localization of the variant-containing region in the secondary structure map of 12S mt-rRNA. Sites of variation are labeled in magenta. Additional sites are labeled in black. Helix numbers are shown in light blue. Symbols: "-", canonical base pair; "•", wobble base pair; "●", non-canonical base pair; thick, black line, physical connection and continuity between adjacent bases that are drawn distantly in the secondary-structure map; red squares connected by red, thick lines, tertiary interactions. **B.** Annotated view of the region containing the **951G>A (m.1598G>A)** variant (yellow and labeled in bold, black font). Other rRNA residues labeled in regular font. 12S mt-rRNA is shown in grey with helix numbers indicated. MRNA is shown as a lime surface. Other components of the mito-ribosome are labeled, and color coded to their molecular model. The position of Y57 of MRPS21/bS21 is indicated. Hydrogen bonds are indicated by blue, broken lines. Distances are denoted with white, broken lines. Water molecules are shown as red spheres. **C.** Chimera X-rendered electron density (black mesh) at contour level of 0.025 (Pettersen et al., 2021). The molecular model and electron density map from the 2.2-Å cryo-EM human mito-ribosomal structure (RCSB ID: 8ANY) (Itoh et al., 2021, Itoh et al., 2022) were used to create panels **B**-**D**.

Original description of putatively non-silent variants in the literature

22U>C (m.669U>C): Study performed with in 250 unrelated Polish patients with non-syndromic and aminoglycoside-induced hearing loss. Homoplasmic. The variant was putatively pathogenic according to authors. “Substitution m.669 T>C was identified only in patients with hearing impairment and episode of aminoglycoside exposure, which may suggest that such additional risk factors must appear to induce clinical phenotype.” (Rydzanicz et al., 2010)

Study performed with 29 families with a clear maternal pattern of inheritance. Probably homoplasmic. Considered putatively pathogenic by authors. (Leveque et al., 2007)

Screening of the general Polish population for deafness-associated mutations. Homoplasmic. Potentially pathogenic according to the authors. (Rydzanicz et al., 2009)

Studied 66 patients with bilateral vestibulopathy. Homoplasmic. “The conclusion of a pathogenic nature is not justified due to the limited sample size”. (Elstner et al., 2008).

62G>A (m.709G>A): Study performed with in 250 unrelated Polish patients with non-syndromic and aminoglycoside-induced hearing loss. Homoplasmic. Common 12S rRNA polymorphism, according to the authors. (Rydzanicz et al., 2010)

Studied 66 patients with bilateral vestibulopathy. Homoplasmic. Common 12S rRNA polymorphism, according to the authors. (Elstner et al., 2008)

Mutational analysis of the mitochondrial 12S rRNA gene in Chinese pediatric subjects with aminoglycoside-induced and non-syndromic hearing loss. Homoplasmic. Not considered pathogenic by the authors. (Li et al., 2005)

The authors performed a clinical and genetic analysis of 169 hearing-impaired patients and some of their relatives suffering from idiopathic sensorineural hearing loss, both familial and sporadic. Homoplasmic status unknown. Common 12S rRNA polymorphism, according to the authors. (Guaran et al., 2013)

Studied 588 independent patients with a presumed hereditary non-syndromic hearing loss. Homoplasmic. Common 12S rRNA polymorphism, according to the authors. (Konings et al., 2008)

63U>C (m.710U>C): Studied 588 independent patients with a presumed hereditary non-syndromic hearing loss. Homoplasmic. Common 12S rRNA polymorphism, according to the authors. (Konings et al., 2008)

65C>A (m.712C>A): The authors performed a clinical and genetic analysis of 169 hearing-impaired patients and some of their relatives suffering from idiopathic sensorineural hearing loss, both familial and sporadic. Homoplasmic. Potentially pathogenic according to the

authors. “The patient harbouring this variant, a 13-year-old subject with mild SNHL (mit184), harboured two additional mutations of A1811G: a polymorphism and a quite rare C>A mutation in the evolutionarily conserved position 3546 in the MT-ND1 gene.” (Guaran et al., 2013)

76A>C (m.723A>C): Study performed with in 250 unrelated Polish patients with non-syndromic and aminoglycoside-induced hearing loss. Homoplasmic. Not considered pathogenic by the authors. (Rydzanicz et al., 2010)

Studied 588 independent patients with a presumed hereditary non-syndromic hearing loss. Homoplasmic. Common 12S rRNA polymorphism, according to the authors. (Konings et al., 2008)

95U>C (m.742U>C): The authors performed a clinical and genetic analysis of 169 hearing-impaired patients and some of their relatives suffering from idiopathic sensorineural hearing loss, both familial and sporadic. Homoplasmic status unknown. Not considered pathogenic by the authors. (Guaran et al., 2013)

98A>G (m.745A>G): Study performed with 1642 Han Chinese pediatric subjects with aminoglycoside-induced and nonsyndromic hearing loss. Homoplasmic. Considered pathogenic by the authors. (Lu et al., 2010)

103G>A (m.750G>A): Study performed with in 250 unrelated Polish patients with non-syndromic and aminoglycoside-induced hearing loss. Homoplasmic. Common 12S rRNA polymorphism, according to the authors. (Rydzanicz et al., 2010)

Study performed with 1642 Han Chinese pediatric subjects with aminoglycoside-induced and nonsyndromic hearing loss. Homoplasmic. Common 12S rRNA polymorphism, according to the authors. (Lu et al., 2010)

Studied 588 independent patients with a presumed hereditary non-syndromic hearing loss. Homoplasmic. Common 12S rRNA polymorphism, according to the authors. (Konings et al., 2008)

Mutational analysis of the mitochondrial 12S rRNA gene in Chinese pediatric subjects with aminoglycoside-induced and non-syndromic hearing loss. Homoplasmic. Not considered pathogenic by the authors. (Li et al., 2005)

The authors performed a clinical and genetic analysis of 169 hearing-impaired patients and some of their relatives suffering from idiopathic sensorineural hearing loss, both familial and sporadic. Homoplasmic status unknown. Common 12S rRNA polymorphism, according to the authors. (Guaran et al., 2013)

122G>A (m.769G>A): Studied 588 independent patients with a presumed hereditary non-syndromic hearing loss. Homoplasmic. Common 12S rRNA polymorphism, according to the authors. (Konings et al., 2008)

Studied 66 patients with bilateral vestibulopathy. Homoplasmic. Common 12S rRNA polymorphism, according to the authors. (Elstner et al., 2008)

123C>U (m.770C>U): Studied 588 independent patients with a presumed hereditary non-syndromic hearing loss. Homoplasmic. Common 12S rRNA polymorphism, according to the authors. (Konings et al., 2008)

139G>A (m.786G>A): The authors performed a clinical and genetic analysis of 169 hearing-impaired patients and some of their relatives suffering from idiopathic sensorineural hearing loss, both familial and sporadic. Heteroplasmic. Considered pathogenic by the authors. “From a clinical point of view, the patient presented with sporadic progressive SNHL with post-lingual onset; her audiometry was compatible with mitochondrial-associated HL and the fact that she was treated with aminoglycosides in the past confirms our hypothesis.” (Guaran et al., 2013)

145C>U (m.792C>U): Study performed with 1642 Han Chinese pediatric subjects with aminoglycoside-induced and nonsyndromic hearing loss. Homoplasmic. Considered pathogenic by the authors. (Lu et al., 2010)

Study performed with 254 maternally inherited and 140 non-syndromic Japanese hearing loss probands with various inheritance modes. Possibly homoplasmic. Considered by the authors as a mitochondrial substitution associated with sensorineural hearing loss with ambiguous status regarding its pathogenicity. (Yano et al., 2014)

178U>A (m.825U>A): Studied 588 independent patients with a presumed hereditary non-syndromic hearing loss. Homoplasmic. Common 12S rRNA polymorphism, according to the authors. (Konings et al., 2008)

180A>G (m.827A>G): Studied 588 independent patients with a presumed hereditary non-syndromic hearing loss. Homoplasmic. Controversial pathogenic nature according to authors. (Konings et al., 2008)

Study performed without hearing loss at the beginning of study and receiving aminoglycosides Patients multi-drug resistant tuberculosis. Possibly homoplasmic. “Audiological data was not available for these mutation-positive individuals.” (Human et al., 2010)

Study performed with 254 maternally inherited and 140 non-syndromic Japanese hearing loss probands with various inheritance modes. Possibly homoplasmic. Considered by the authors as a mitochondrial substitution associated with sensorineural hearing loss with ambiguous status regarding its pathogenicity. (Yano et al., 2014)

Study performed with in 250 unrelated Polish patients with non-syndromic and aminoglycoside-induced hearing loss. Homoplasmic. The variant was putatively pathogenic according to authors. “The patient carrying the m.827 A>G substitution suffers moderate HL after aminoglycoside treatment in an early childhood, and her haplogroup was defined as H8.” (Rydzanicz et al., 2010)

Study performed with 164 Caucasian subjects, who were younger than 19 years and had been diagnosed with non-syndromic sensorineural hearing impairment. Homoplasmic. Considered potentially pathogenic by the authors. (Li et al., 2004)

Studied a four generation Chinese family with 40 members. Homoplasmic. It concluded that the mutation was pathogenic with low penetrance. (Xing et al., 2006)

Mutational analysis of the mitochondrial 12S rRNA gene in Chinese pediatric subjects with aminoglycoside-induced and non-syndromic hearing loss. Homoplasmic. Potential role in the pathogenesis of aminoglycoside ototoxicity according to the authors. (Li et al., 2005)

Studied two Argentinian sisters who developed hearing loss. Homoplasmic. It concluded that the mutation was potentially pathogenic with low penetrance. (Chaig et al., 2008)

209A>G (m.856A>G) Study performed with 1642 Han Chinese pediatric subjects with aminoglycoside-induced and nonsyndromic hearing loss. Homoplasmic. Considered pathogenic by the authors. (Lu et al., 2010)

Study performed with 254 maternally inherited and 140 non-syndromic Japanese hearing loss probands with various inheritance modes. Possibly homoplasmic. Considered by the authors as a mitochondrial substitution associated with sensorineural hearing loss with ambiguous status regarding its pathogenicity. (Yano et al., 2014)

283G>A (m.930G>A): Studied 588 independent patients with a presumed hereditary non-syndromic hearing loss. Homoplasmic. Common 12S rRNA polymorphism, according to the authors. (Konings et al., 2008)

Study performed with in 250 unrelated Polish patients with non-syndromic and aminoglycoside-induced hearing loss. Homoplasmic. Common 12S rRNA polymorphism, according to the authors. (Rydzanicz et al., 2010)

The authors performed a clinical and genetic analysis of 169 hearing-impaired patients and some of their relatives suffering from idiopathic sensorineural hearing loss, both familial and sporadic. Homoplasmic status unknown. Common 12S rRNA polymorphism, according to the authors. (Guaran et al., 2013)

295A>G (m.942A>G): The authors performed a clinical and genetic analysis of 169 hearing-impaired patients and some of their relatives suffering from idiopathic sensorineural hearing loss, both familial and sporadic. Homoplasmic status unknown. Not considered pathogenic by the authors. (Guaran et al., 2013)

Study performed with 1642 Han Chinese pediatric subjects with aminoglycoside-induced and nonsyndromic hearing loss. Homoplasmic. Common 12S rRNA polymorphism, according to the authors. (Lu et al., 2010)

304G>A (m.951G>A): Study performed with in 250 unrelated Polish patients with non-syndromic and aminoglycoside-induced hearing loss. Homoplasmic. Not considered pathogenic by the authors. (Rydzanicz et al., 2010)

Studied 66 patients with bilateral vestibulopathy. Homoplasmic. Pathogenic status inconclusive. (Elstner et al., 2008)

Study performed with 1642 Han Chinese pediatric subjects with aminoglycoside-induced and nonsyndromic hearing loss. Homoplasmic. Pathogenic status unclear. (Lu et al., 2010)

Studied 588 independent patients with a presumed hereditary non-syndromic hearing loss. Homoplasmic. Common 12S rRNA polymorphism, according to the authors. (Konings et al., 2008)

The authors performed a clinical and genetic analysis of 169 hearing-impaired patients and some of their relatives suffering from idiopathic sensorineural hearing loss, both familial and sporadic. Homoplasmic status unknown. Common 12S rRNA polymorphism, according to the authors. (Guaran et al., 2013)

333U>C (m.980U>C): The authors performed a clinical and genetic analysis of 169 hearing-impaired patients and some of their relatives suffering from idiopathic sensorineural hearing loss, both familial and sporadic. Homoplasmic status unknown. Not considered pathogenic by the authors. (Guaran et al., 2013)

341G>A (m.988G>A): Study performed with in 250 unrelated Polish patients with non-syndromic and aminoglycoside-induced hearing loss. Homoplasmic. Proposed as a new candidate for genetic risk factors of non-syndromic and aminoglycoside-induced hearing impairment. (Rydzanicz et al., 2010)

Studied 588 independent patients with a presumed hereditary non-syndromic hearing loss. Homoplasmic. Common 12S rRNA polymorphism, according to the authors. (Konings et al., 2008)

343U>C (m.990U>C): Studied 588 independent patients with a presumed hereditary non-syndromic hearing loss. Homoplasmic. Unknown pathogenic status according to the authors. (Konings et al., 2008)

358U>C (m.1005U>C): Mutational analysis of the mitochondrial 12S rRNA gene in Chinese pediatric subjects with aminoglycoside-induced and non-syndromic hearing loss. Homoplasmic. Potential role in the pathogenesis of aminoglycoside ototoxicity according to the authors. (Li et al., 2005)

Study performed with 254 maternally inherited and 140 non-syndromic Japanese hearing loss probands with various inheritance modes. Possibly homoplasmic. Considered by the authors as a mitochondrial substitution associated with sensorineural hearing loss with ambiguous-status regarding its pathogenicity.(Yano et al., 2014)

360G>A (m.1007G>A): Studied 66 patients with bilateral vestibulopathy. Homoplasmic. Not considered pathogenic by the authors. (Elstner et al., 2008)

361A>G (m.1008A>G): Studied 588 independent patients with a presumed hereditary non-syndromic hearing loss. Heteroplasmic. Common 12S rRNA polymorphism, according to the authors. (Konings et al., 2008)

400A (m.1047A): Study performed with in 250 unrelated Polish patients with non-syndromic and aminoglycoside-induced hearing loss. Homoplasmic. No comment by the authors on this variant. (Rydzanicz et al., 2010)

401C (m.1048C): Studied 588 independent patients with a presumed hereditary non-syndromic hearing loss. Homoplasmic. Common 12S rRNA polymorphism, according to the authors. (Konings et al., 2008)

448U>C (m.1095U>C): Study performed with 254 maternally inherited and 140 non-syndromic Japanese hearing loss probands with various inheritance modes. Possibly homoplasmic. Considered by the authors as a mitochondrial substitution associated with sensorineural hearing loss with ambiguous-status regarding its pathogenicity. (Yano et al., 2014)

According to authors, the variant was found in homoplasmy in a “Child who had a strong evidence for a mtDNA defect based on family history (male and female were equally affected in his family, the trait was always transmitted by females, the affected male did not transmit the disorder) and a past medical history of aminoglycoside-induced deafness (AID) in two maternal relatives (I-1 and II-1). Molecular analyses also identified the T1095C mutation in the three maternal relatives who agreed to undergo genetic testing….This mutation fulfils the suggested criteria for definition of a disease-related nucleotide variant.” (Tessa et al., 2001)

Study reporting on the “clinical and sequence analysis of the entire mitochondrial genome in three Chinese subjects with aminoglycoside-induced and non-syndromic hearing impairment.” Homoplasmic. Possibly associated with hearing impairment according to authors. (Zhao et al., 2004)

According to authors, a heteroplasmic mtDNA mutation (T1095C) in the 12SrRNA gene was found in an “Italian family with features of maternally-inherited parkinsonism, antibiotic-mediated deafness and peripheral neuropathy... A transmitochondrial cybrid line derived from the proband of this family shows selective depletion of mitochondrial glutathione and decreases in the activity of complex II/III. Moreover, when exposed to an aminoglycoside antibiotic these cells responded with a ten-fold increase in the number of apoptotic cells compared to controls. These results support a pathogenic role for the T1095C mutation and indicate that the mutation increases the risk for aminoglycoside-induced toxicity.” (Muyderman et al., 2012)

459C>U (m.1106C>U): Studied 588 independent patients with a presumed hereditary non-syndromic hearing loss. Heteroplasmic. Unknown pathogenic status according to the authors. (Konings et al., 2008)

460U>C (m.1107U>C): Studied 128 Chinese pediatric subjects with sporadic aminoglycoside-induced and non-syndromic hearing loss. Homoplasmic. Not considered pathogenic by the authors. (Li et al., 2005)

469A>G (m.1116A>G): Study performed with 1642 Han Chinese pediatric subjects with aminoglycoside-induced and nonsyndromic hearing loss. Homoplasmic. Pathogenic status unclear. (Lu et al., 2010)

471A>G (m.1118A>G): The authors performed a clinical and genetic analysis of 169 hearing-impaired patients and some of their relatives suffering from idiopathic sensorineural hearing loss, both familial and sporadic. Homoplasmic status unknown. Not considered pathogenic by the authors. (Guaran et al., 2013)

472U>C (m.1119U>C): Mutational analysis of the mitochondrial 12S rRNA gene in Chinese pediatric subjects with aminoglycoside-induced and non-syndromic hearing loss. Homoplasmic. Not considered pathogenic by the authors. (Li et al., 2005)

The authors performed a clinical and genetic analysis of 169 hearing-impaired patients and some of their relatives suffering from idiopathic sensorineural hearing loss, both familial and sporadic. Homoplasmic status unknown. “We suggest that this variant detected in 36-year-old female may be responsible for her mild progressive bilateral SNHL.” (Guaran et al., 2013)

533U>G (m.1180U>G): Study performed with 164 Caucasian subjects, who were younger than 19 years and had been diagnosed with non-syndromic sensorineural hearing impairment. Homoplasmic. Considered potentially pathogenic by the authors. (Li et al., 2004)

542U>C (m.1189U>C): Studied 588 independent patients with a presumed hereditary non-syndromic hearing loss. Homoplasmic. Common 12S rRNA polymorphism, according to the authors. (Konings et al., 2008)

Study performed with 1642 Han Chinese pediatric subjects with aminoglycoside-induced and nonsyndromic hearing loss. Homoplasmic. Common 12S rRNA polymorphism, according to the authors. (Lu et al., 2010)

“Two hearing-impaired patients that had been treated with streptomycin had the T1189C variant of the mitochondrial 12S rRNA region”. Homoplasmic. Considered potentially pathogenic by the authors. (Meza et al., 2011)

The authors performed a clinical and genetic analysis of 169 hearing-impaired patients and some of their relatives suffering from idiopathic sensorineural hearing loss, both familial and sporadic. Homoplasmic status unknown. Common 12S rRNA polymorphism, according to the authors. (Guaran et al., 2013)

Study performed with in 250 unrelated Polish patients with non-syndromic and aminoglycoside-induced hearing loss. Homoplasmic. Common 12S rRNA polymorphism, according to the authors. (Rydzanicz et al., 2010)

545C>U/A (m.1192C>U/A): Study performed with 1642 Han Chinese pediatric subjects with aminoglycoside-induced and nonsyndromic hearing loss. Homoplasmic. Both considered pathogenic by the authors. (Lu et al., 2010)

546U>C (m.1193U>C): The authors performed a clinical and genetic analysis of 169 hearing-impaired patients and some of their relatives suffering from idiopathic sensorineural hearing loss, both familial and sporadic. Homoplasmic status unknown. Not considered pathogenic by the authors. (Guaran et al., 2013)

579C>G (m.1226C>G): Study performed with 164 Caucasian subjects, who were younger than 19 years and had been diagnosed with non-syndromic sensorineural hearing impairment. Homoplasmic. Considered potentially pathogenic by the authors. (Li et al., 2004)

596U>C (m.1243U>C): The authors performed a clinical and genetic analysis of 169 hearing-impaired patients and some of their relatives suffering from idiopathic sensorineural hearing loss, both familial and sporadic. Homoplasmic status unknown. Common 12S rRNA polymorphism, according to the authors. (Guaran et al., 2013)

Study performed with in 250 unrelated Polish patients with non-syndromic and aminoglycoside-induced hearing loss. Homoplasmic. Common 12S rRNA polymorphism, according to the authors. (Rydzanicz et al., 2010)

Studied 588 independent patients with a presumed hereditary non-syndromic hearing loss. Homoplasmic. Common 12S rRNA polymorphism, according to the authors. (Konings et al., 2008)

Analyzed 443 families with hearing impairment. Homoplasmic. A pathogenic role could not be ruled out by the authors. (Ballana et al., 2006)

663C>U (m.1310C>U): Study performed with 254 maternally inherited and 140 non-syndromic Japanese hearing loss probands with various inheritance modes. Possibly homoplasmic. Considered by the authors as a mitochondrial substitution associated with sensorineural hearing loss with ambiguous-status regarding its pathogenicity. (Yano et al., 2014)

684A>G (m.1331A>G): Study performed with 1642 Han Chinese pediatric subjects with aminoglycoside-induced and nonsyndromic hearing loss. Homoplasmic. Considered pathogenic by the authors. (Lu et al., 2010)

735A>C (m.1382A>C): Mutational analysis of the mitochondrial 12S rRNA gene in Chinese pediatric subjects with aminoglycoside-induced and non-syndromic hearing loss. Homoplasmic. Not considered pathogenic by the authors. (Li et al., 2005)

791G (m.1438G): Study performed with in 250 unrelated Polish patients with non-syndromic and aminoglycoside-induced hearing loss. Homoplasmic. Common 12S rRNA polymorphism, according to the authors. (Rydzanicz et al., 2010)

Studied 588 independent patients with a presumed hereditary non-syndromic hearing loss. Homoplasmic. Common 12S rRNA polymorphism, according to the authors. (Konings et al., 2008)

Mutational analysis of the mitochondrial 12S rRNA gene in Chinese pediatric subjects with aminoglycoside-induced and non-syndromic hearing loss. Homoplasmic. Not considered pathogenic by the authors. (Li et al., 2005)

The authors performed a clinical and genetic analysis of 169 hearing-impaired patients and some of their relatives suffering from idiopathic sensorineural hearing loss, both familial and

sporadic. Homoplasmic status unknown. Common 12S rRNA polymorphism, according to the authors. (Guaran et al., 2013)

796U>C (m.1443U>C): Mutational analysis of the mitochondrial 12S rRNA gene in Chinese pediatric subjects with aminoglycoside-induced and non-syndromic hearing loss. Homoplasmic. Not considered pathogenic by the authors. (Li et al., 2005)

805U>C (m.1452U>C): Study performed with 1642 Han Chinese pediatric subjects with aminoglycoside-induced and nonsyndromic hearing loss. Homoplasmic. Considered pathogenic by the authors. (Lu et al., 2010)

806A>G (m.1453A>G): Study performed with in 250 unrelated Polish patients with non-syndromic and aminoglycoside-induced hearing loss. Homoplasmic. Proposed as a new candidate for genetic risk factors of non-syndromic and aminoglycoside-induced hearing impairment. (Rydzanicz et al., 2010)

Study performed with 303 unrelated patients (including family members of 25 probands) with nonsyndromic hearing loss. Homoplasmic. Considered a “possibly deleterious mutation” by the authors. (Padma et al., 2012)

815G>A (m.1462G>A): Studied 66 patients with bilateral vestibulopathy. Homoplasmic status unknown. Common 12S rRNA polymorphism, according to the authors. (Elstner et al., 2008)

Study performed with 1642 Han Chinese pediatric subjects with aminoglycoside-induced and nonsyndromic hearing loss. Homoplasmic. Common 12S rRNA polymorphism, according to the authors. (Lu et al., 2010)

Studied 588 independent patients with a presumed hereditary non-syndromic hearing loss. Homoplasmic. Common 12S rRNA polymorphism, according to the authors. (Konings et al., 2008)

Study performed with 303 unrelated patients (including family members of 25 probands) with nonsyndromic hearing loss. Homoplasmic. Unclear pathogenic status according to the authors. (Padma et al., 2012)

826C>U (m.1473C>U): Studied 97 hearing-impaired Chinese probands. Homoplasmic. Considered a new candidate variant for hearing loss by the authors. (Chen et al., 2018)

856G>A (m.1503G>A): Study performed with in 250 unrelated Polish patients with non-syndromic and aminoglycoside-induced hearing loss. Homoplasmic. Not considered pathogenic by the authors. (Rydzanicz et al., 2010)

Genetic Mutations in Non-syndromic Deafness Patients of Uyghur and Han Chinese Ethnicities in Xinjiang, China. Homoplasmic. Unclear pathogenic status according to the authors. (Chen et al., 2011)

861C>U (m.1508C>U): Study performed with 303 unrelated patients (including family members of 25 probands) with nonsyndromic hearing loss. Homoplasmic. Unclear status according to the authors. (Padma et al., 2012)

878C>G (m.1525C>G): Patient with profound hearing loss. Homoplasmic status unknown. This work. (Smith et al., 2014)

890C>U (m.1537C>U): Study performed with 29 families with a clear maternal pattern of inheritance. Homoplasmic status unclear. Considered putatively pathogenic by authors. (Leveque et al., 2007)

Studied 588 independent patients with a presumed hereditary non-syndromic hearing loss. Homoplasmic. Common 12S rRNA polymorphism, according to the authors. (Konings et al., 2008)

910A>C (m.1557A>C): Variant found in a deafness-suffering individual. Considered a polymorphism by the authors. No additional information available. (Tazetdinov et al., 2007)

951G>A (m.1598G>A): Mutational analysis of the mitochondrial 12S rRNA gene in Chinese pediatric subjects with aminoglycoside-induced and non-syndromic hearing loss. Homoplasmic. Not considered pathogenic by the authors. (Li et al., 2005)

References

Abdi NM, Fredrick K. Contribution of 16S rRNA nucleotides forming the 30S subunit A and P sites to translation in Escherichia coli. RNA (2005) 11:1624-32 doi: 10.1261/rna.2118105.

Abreu-Silva RS, Batissoco AC, Lezirovitz K, Romanos J, Rincon D, Auricchio MT, et al. Correspondence regarding Ballana et al., "Mitochondrial 12S rRNA gene mutations affect RNA secondary structure and lead to variable penetrance in hearing impairment". Biochem Biophys Res Commun (2006) 343:675-6 doi: 10.1016/j.bbrc.2006.03.049.

Amunts A, Brown A, Toots J, Scheres SH, Ramakrishnan V. The structure of the human mitochondrial ribosome. Science (2015) 348:95-8 doi: 10.1126/science.aaa1193.

Ananth P, Goldsmith G, Yathindra N. An innate twist between Crick's wobble and Watson-Crick base pairs. *RNA* (2013) **19**:1038-53 doi: 10.1261/rna.036905.112.

Ballana E, Morales E, Estivill X. Reply to correspondence by Abreu-Silva et al. regarding Ballana et al.: Mutation T1291C in the mitochondrial 12S rRNA gene involved in deafness in a Cuban family belongs to the macrohaplogroup L1 of African origin. Biochem Biophys Res Commun (2006a) 346:619-20 doi: 10.1016/j.bbrc.2006.05.098.

Ballana E, Morales E, Rabionet R, Montserrat B, Ventayol M, Bravo O, et al. Mitochondrial 12S rRNA gene mutations affect RNA secondary structure and lead to variable penetrance in hearing impairment. *Biochem Biophys Res Commun* (2006b) **341**:950-7 doi: 10.1016/j.bbrc.2006.01.049.

Bursle C, Narendra A, Chuk R, Cardinal J, Justo R, Lewis B, et al. COXPD9 an Evolving Multisystem Disease; Congenital Lactic Acidosis, Sensorineural Hearing Loss, Hypertrophic Cardiomyopathy, Cirrhosis and Interstitial Nephritis. JIMD Rep (2017) 34:105-9 doi: 10.1007/8904_2016_13.

Carroll CJ, Isohanni P, Poyhonen R, Euro L, Richter U, Brilhante V, et al. Whole-exome sequencing identifies a mutation in the mitochondrial ribosome protein MRPL44 to underlie mitochondrial infantile cardiomyopathy. J Med Genet (2013) 50:151-9 doi: 10.1136/jmedgenet-2012-101375.

Chaig MR, Zernotti ME, Soria NW, Romero OF, Romero MF, Gerez NM. A mutation in mitochondrial 12S rRNA, A827G, in Argentinean family with hearing loss after aminoglycoside treatment. *Biochem Biophys Res Commun* (2008) **368**:631-6 doi: 10.1016/j.bbrc.2008.01.143.

Chen X, Wang F, Maerhaba A, Li Q, Wang J, Liu X, et al. Novel mitochondrial gene variants in Northwestern Chinese probands with non-syndromic hearing loss by whole mitochondrial genome screening. *Gene* (2018) **652**:59-65 doi: 10.1016/j.gene.2018.01.098.

Chen Y, Tudi M, Sun J, He C, Lu HL, Shang Q, et al. Genetic mutations in non-syndromic deafness patients of Uyghur and Han Chinese ethnicities in Xinjiang, China: a comparative study. *J Transl Med* (2011) **9**:154 doi: 10.1186/1479-5876-9-154.

Distelmaier F, Haack TB, Catarino CB, Gallenmuller C, Rodenburg RJ, Strom TM, et al. MRPL44 mutations cause a slowly progressive multisystem disease with childhood-onset hypertrophic cardiomyopathy. Neurogenetics (2015) 16:319-23 doi: 10.1007/s10048-015-0444-2.

Elstner M, Schmidt C, Zingler VC, Prokisch H, Bettecken T, Elson JL, et al. Mitochondrial 12S rRNA susceptibility mutations in aminoglycoside-associated and idiopathic bilateral vestibulopathy. *Biochem Biophys Res Commun* (2008) **377**:379-83 doi: 10.1016/j.bbrc.2008.09.134.

Estivill X, Fortina P, Surrey S, Rabionet R, Melchionda S, D'Agruma L, et al. Connexin-26 mutations in sporadic and inherited sensorineural deafness. The Lancet (1998b) 351:394-8 doi: 10.1016/S0140-6736(97)11124-2.

Farhadi M, Houshmand M, Balahi M, Falah M. MITOMAP mtDNA Sequence Data: Submitted variant 20161022001. (2008) Available from: https://www.mitomap.org/foswiki/bin/view/MITOMAP/Submissions/20161022001.

Galmiche L, Serre V, Beinat M, Assouline Z, Lebre AS, Chretien D, et al. Exome sequencing identifies MRPL3 mutation in mitochondrial cardiomyopathy. Hum Mutat (2011) 32:1225-31 doi: 10.1002/humu.21562.
Greber BJ, Bieri P, Leibundgut M, Leitner A, Aebersold R, Boehringer D, et al. The complete structure of the 55S mammalian mitochondrial ribosome. Science (2015) 348:303-8 doi: 10.1126/science.aaa3872.

Guaran V, Astolfi L, Castiglione A, Simoni E, Olivetto E, Galasso M, et al. Association between idiopathic hearing loss and Humanmitochondrial DNA mutations: a study on 169 hearing-impaired subjects. *Int J Mol Med* (2013) **32**:785-94 doi: 10.3892/ijmm.2013.1470.

Haumann S, Boix J, Knuever J, Bieling A, Vila Sanjurjo A, Elson JL, et al. Mitochondrial DNA mutations induce mitochondrial biogenesis and increase the tumorigenic potential of Hodgkin and Reed-Sternberg cells. Carcinogenesis (2020) doi: 10.1093/carcin/bgaa032.

Human H, Hagen CM, de Jong G, Harris T, Lombard D, Christiansen M, et al. Investigation of mitochondrial sequence variants associated with aminoglycoside-induced ototoxicity in South African TB patients on aminoglycosides. *Biochem Biophys Res Commun* (2010) **393**:751-6 doi: 10.1016/j.bbrc.2010.02.075.

Igumnova V, Veidemane L, Viksna A, Capligina V, Zole E, Ranka R. The prevalence of mitochondrial mutations associated with aminoglycoside-induced deafness in ethnic Latvian population: the appraisal of the evidence. J Hum Genet (2019) 64:199-206 doi: 10.1038/s10038-018-0544-6.

Itoh Y, Singh V, Khawaja A, Naschberger A, Nguyen MD, Rorbach J, et al. Structure of the mitoribosomal small subunit with streptomycin reveals Fe-S clusters and physiological molecules. *Elife* (2022) **11**:10.7554/eLife.77460 doi: 10.7554/eLife.77460.

Itoh Y, Andrell J, Choi A, Richter U, Maiti P, Best RB, et al. Mechanism of membrane-tethered mitochondrial protein synthesis. *Science* (2021) **371**:846-9 doi: 10.1126/science.abe0763.

Khawaja A, Itoh Y, Remes C, Spahr H, Yukhnovets O, Hofig H, et al. Distinct pre-initiation steps in human mitochondrial translation. *Nat Commun* (2020) **11**(1):2932 doi: 10.1038/s41467-020-16503-2.

Konings A, Van Camp G, Goethals A, Van Eyken E, Vandevelde A, Ben Azza J, et al. Mutation analysis of mitochondrial DNA 12SrRNA and tRNASer(UCN) genes in non-syndromic hearing loss patients. *Mitochondrion* (2008) **8**:377-82 doi: 10.1016/j.mito.2008.08.001.

Koripella RK, Sharma MR, Bhargava K, Datta PP, Kaushal PS, Keshavan P, et al. Structures of the human mitochondrial ribosome bound to EF-G1 reveal distinct features of mitochondrial translation elongation. *Nat Commun* (2020) **11**(1):3830 doi: 10.1038/s41467-020-17715-2.

Kummer E, Schubert KN, Schoenhut T, Scaiola A, Ban N. Structural basis of translation termination, rescue, and recycling in mammalian mitochondria. Mol Cell (2021) 81:2566,2582.e6 doi: 10.1016/j.molcel.2021.03.042.

Lancaster L, Noller HF. Involvement of 16S rRNA nucleotides G1338 and A1339 in discrimination of initiator tRNA. Mol Cell (2005) 20:623-32 doi: S1097-2765(05)01676-X.

Leveque M, Marlin S, Jonard L, Procaccio V, Reynier P, Amati-Bonneau P, et al. Whole mitochondrial genome screening in maternally inherited non-syndromic hearing impairment using a microarray resequencing mitochondrial DNA chip. *Eur J Hum Genet* (2007) **15**:1145-55 doi: 10.1038/sj.ejhg.5201891.

Li R, Greinwald JH,Jr, Yang L, Choo DI, Wenstrup RJ, Guan MX. Molecular analysis of the mitochondrial 12S rRNA and tRNASer(UCN) genes in paediatric subjects with non-syndromic hearing loss. *J Med Genet* (2004) **41**:615-20 doi: 10.1136/jmg.2004.020230.

Li Z, Li R, Chen J, Liao Z, Zhu Y, Qian Y, et al. Mutational analysis of the mitochondrial 12S rRNA gene in Chinese pediatric subjects with aminoglycoside-induced and non-syndromic hearing loss. *Hum Genet* (2005) **117**:9-15 doi: 10.1007/s00439-005-1276-1.

Lu J, Li Z, Zhu Y, Yang A, Li R, Zheng J, et al. Mitochondrial 12S rRNA variants in 1642 Han Chinese pediatric subjects with aminoglycoside-induced and nonsyndromic hearing loss. *Mitochondrion* (2010) **10**:380-90 doi: 10.1016/j.mito.2010.01.007.

Meza G, Torres-Ruiz NM, Tirado-Gutierrez C, Aguilera P. mtDNA mutations, hearing loss and aminoglycoside treatment in Mexicans. *Braz J Otorhinolaryngol* (2011) **77**:573-6. doi: 10.1590/s1808-86942011000500006.

Mkaouar-Rebai E, Fendri-Kriaa N, Louhichi N, Tlili A, Triki C, Ghorbel A, et al. Whole mitochondrial genome screening in two families with hearing loss: detection of a novel mutation in the 12S rRNA gene. Biosci Rep (2010) 30:405-11 doi: 10.1042/BSR20090120.

Mkaouar-Rebai E, Tlili A, Masmoudi S, Charfeddine I, Fakhfakh F. New polymorphic mtDNA restriction site in the 12S rRNA gene detected in Tunisian patients with non-syndromic hearing loss. Biochem Biophys Res Commun (2008) 369:849-52 doi: 10.1016/j.bbrc.2008.02.107.

Muyderman H, Sims NR, Tanaka M, Fuku N, Raghupathi R, Thyagarajan D. The mitochondrial T1095C mutation increases gentamicin-mediated apoptosis. *Mitochondrion* (2012) **12**:465-71 doi: 10.1016/j.mito.2012.06.006.

Nolden M, Ehses S, Koppen M, Bernacchia A, Rugarli EI, Langer T. The m-AAA protease defective in hereditary spastic paraplegia controls ribosome assembly in mitochondria. Cell (2005) 123:277-89 doi: S0092-8674(05)00806-8.

Noller HF. RNA structure: reading the ribosome. Science (2005) 309:1508-14 doi: 10.1126/science.1111771.

Padma G, Ramchander PV. MITOMAP mtDNA Sequence Data: Submitted variant 20081105001. (2008) Available from: https://www.mitomap.org/foswiki/bin/view/MITOMAP/Submissions/20081105001.

Pettersen EF, Goddard TD, Huang CC, Meng EC, Couch GS, Croll TI, et al. UCSF ChimeraX: Structure visualization for researchers, educators, and developers. *Protein Sci* (2021) **30**:70-82 doi: 10.1002/pro.3943.

Ruiz-Pesini E, Lott MT, Procaccio V, Poole JC, Brandon MC, Mishmar D, et al. An enhanced MITOMAP with a global mtDNA mutational phylogeny. Nucleic Acids Res (2007) 35:D823-8 doi: 10.1093/nar/gkl927.

Rydzanicz M, Wrobel M, Pollak A, Gawecki W, Brauze D, Kostrzewska-Poczekaj M, et al. Mutation analysis of mitochondrial 12S rRNA gene in Polish patients with non-syndromic and aminoglycoside-induced hearing loss. *Biochem Biophys Res Commun* (2010) **395**:116-21 doi: 10.1016/j.bbrc.2010.03.149.

Rydzanicz M, Wrobel M, Cywinska K, Froehlich D, Gawecki W, Szyfter W, et al. Screening of the general Polish population for deafness-associated mutations in mitochondrial 12S rRNA and tRNA Ser(UCN) genes. *Genet Test Mol Biomarkers* (2009) **13**:167-72 doi: 10.1089/gtmb.2008.0098.

Smith PM, Elson JL, Greaves LC, Wortmann SB, Rodenburg RJ, Lightowlers RN, et al. The role of the mitochondrial ribosome in human disease: Searching for mutations in 12S mitochondrial rRNA with high disruptive potential. *Hum Mol Genet* (2014) **23**:949-56 doi: 10.1093/hmg/ddt490.

Tang HY, Hutcheson E, Neill S, Drummond-Borg M, Speer M, Alford RL. Genetic susceptibility to aminoglycoside ototoxicity: how many are at risk? Genet Med (2002) 4:336-45 doi: 10.1097/01.GIM.0000029035.91778.53.

Tang J, Qi Y, Bao XH, Wu XR. Mutational analysis of mitochondrial DNA of children with Rett syndrome. Pediatr Neurol (1997) 17:327-30. doi: 10.1016/s0887-8994(97)00151-3.

Tazetdinov AM, Dzhemileva, LU, Ponidelko SN, Markova TG, Khusnutdinova EK. MITOMAP mtDNA Sequence Data: Unpublished Variant 20070709002. (2007) Available from: http://www.mitomap.org/bin/view.pl/MITOMAP/Submissions/20070709002.

Tessa A, Giannotti A, Tieri L, Vilarinho L, Marotta G, Santorelli FM. Maternally inherited deafness associated with a T1095C mutation in the mDNA. *Eur J Hum Genet* (2001**)** 9:147-9 doi: 10.1038/sj.ejhg.5200601.

Wang F, Zhang D, Zhang D, Li P, Gao Y. Mitochondrial Protein Translation: Emerging Roles and Clinical Significance in Disease. Front Cell Dev Biol (2021) 9:675465 doi: 10.3389/fcell.2021.675465.

Xing G, Chen Z, Wei Q, Tian H, Li X, Zhou A, et al. Maternally inherited non-syndromic hearing loss associated with mitochondrial 12S rRNA A827G mutation in a Chinese family. *Biochem Biophys Res Commun* (2006**) 34**4:1253-7 doi: 10.1016/j.bbrc.2006.04.033.

Yano T, Nishio SY, Usami S, Deafness Gene Study Consortium. Frequency of mitochondrial mutations in non-syndromic hearing loss as well as possibly responsible variants found by whole mitochondrial genome screening. *J Hum Genet* (2014**) 5**9:100-6 doi: 10.1038/jhg.2013.128.

Zhao L, Young WY, Li R, Wang Q, Qian Y, Guan MX. Clinical evaluation and sequence analysis of the complete mitochondrial genome of three Chinese patients with hearing impairment associated with the 12S rRNA T1095C mutation. *Biochem Biophys Res Commun* (2004b**) 32**5:1503-8 doi: 10.1016/j.bbrc.2004.10.199.
